# Human iPSC-cardiomyocyte-aggregate cell therapy in non-human primates and correlation of heart recovery with contractile and electrophysiological cardiomyocyte properties

**DOI:** 10.1038/s41467-026-77103-0

**Published:** 2026-08-26

**Authors:** Ina Gruh, Andreas Martens, Serghei Cebotari, Annette Schrod, Alexandra Haase, Caroline Halloin, Wiebke Triebert, Nils Kriedemann, Tobias Goecke, Morsi Arar, Klaus Hoeffler, Paul Frank, Karen Lampe, Amir Moussavi, Anna Kuleshova, Vojtech Hradil, Veronika Fricke, Monika Szepes, Kerstin Mätz-Rensing, Jörg Eiringhaus, Anna-Lena de Vries, Stephan Hohmann, Merlin Witte, Tim Kohrn, Jana Teske, Kevin Cyrys, Sara Szadocka, Annika Franke, Christopher Werlein, Karlo Komorowski, Mark Kühnel, Danny Jonigk, Susann Boretius, Christian Veltmann, David Duncker, Jonathan Jung, Nissim Benvenisty, Jeanne de la Roche, Martin Fischer, Axel Haverich, Arjang Ruhparwar, Robert Zweigerdt, Ulrich Martin

**Affiliations:** 1 Leibniz Research Laboratories for Biotechnology and Artificial Organs, Cluster of Excellence REBIRTH, Carl-Neuberg-Straße 1, Hannover Medical School, Hannover, Germany; 2 Dept. of Cardiac, Thoracic, Transplantation and Vascular Surgery, Cluster of Excellence REBIRTH, Hannover Medical School, Hannover, Germany; 3 Dept. of Cardiac Surgery, Carl von Ossietzky University Oldenburg, Oldenburg, Germany; 4 Dept. of Cardiac Surgery, Institute for Cardiac Surgery and Interventional Cardiology, INCCI Heart-Center Foundation, Luxembourg, Luxembourg; 5 German Primate Center, Leibniz Institute for Primate Research, Göttingen, Germany; 6 Biomedical Research in Endstage and Obstructive Lung Disease (BREATH), German Center for Lung Research (DZL), Hannover Medical School, Hannover, Germany; 7 Hannover Heart Rhythm Center, Department of Cardiology and Angiology, Hannover Medical School, Hannover, Germany; 8 Department of Pathology, Hannover Medical School, Hannover, Germany; 9 Department of Radiation Protection and Medical Physics, Hannover Medical School, Hannover, Germany; 10 Institute of Pathology, University Hospital RWTH Aachen, Aachen, Germany; 11 Department of Genetics, The Hebrew University of Jerusalem, Jerusalem, Israel; 12 Institute of Neurophysiology, Hannover Medical School, Hannover, Germany

**Keywords:** Stem-cell research, Induced pluripotent stem cells, Heart stem cells, Cardiology

## Abstract

This study demonstrates the successful production and injection of human induced pluripotent stem cell cardiomyocyte aggregates into infarcted cynomolgus monkey hearts, resulting in substantial, structured human grafts three months after cell transplantation. Transient graft-induced arrhythmias decreased over time. Both the arrhythmogenicity and the substantial heart function recovery in vivo notably seemed to correlate with induced pluripotent stem cell clone-dependent contractile and electrophysiological cardiomyocyte properties in vitro. Overexpression of a red fluorescent reporter protein led to a dysregulated conduction and contraction machinery in yet engraftment competent cardiomyocytes, providing an important tool to mechanistically understand and improve induced pluripotent stem cell-based heart repair in preclinical models.

We demonstrate the logistically important, temporal uncoupling of cardiomyocyte production from transplantation. Cardiomyocyte aggregate transplantation yielded results comparable to the reported transplantation of 10-20-fold higher numbers of dissociated human embryonic stem cell- cardiomyocytes and suggests a higher degree of cell/ tissue maturation in cardiac grafts. Our study promotes reduced cell production costs, highlights the need for an in vitro potency assay, and shows a pragmatic new avenue for the clinical translation of human induced pluripotent stem cell-based heart repair.

## Abstract (web summary)

The study shows successful therapy with hiPSC-cardiomyocyte aggregates in infarcted non-human primates, including uncoupling of cell production from transplantation and correlation of heart recovery in vivo with cardiomyocyte properties in vitro.

## Introduction

Heart failure (HF) remains a major cause of morbidity and mortality worldwide. Cellular therapies based on human induced pluripotent stem cells (iPSCs, hiPSCs) for the first time offer the de novo generation of bona fide human cardiomyocytes (CMs) in vitro for replacing lost heart muscle cells in situ. Currently, iPSC-based approaches are considered innovative but complex therapeutic concepts for treating HF resulting from conditions such as ischemic cardiomyopathy or myocardial infarction (MI). While critical research on safety aspects of iPSC-based cell transplants is ongoing^1^, substantial progress on iPSC technologies was achieved, including the development of controlled, efficient bioprocesses for the expansion and lineage-directed differentiation of iPSCs into functional well-characterised cardiomyocytes (iPSC-CMs) at clinical scale^2–4^.

Preclinical studies investigating pluripotent stem cell (PSC)-derived CMs were conducted already in non-human primates (NHP), since the high heart beating rates in rodent models (with the possible exception of guinea pigs^5^) prevent the proper electromechanical coupling of human CMs^6,7^. In addition, the measurement of heart function parameters is also less accurate in small animals whilst the prolonged survival of human cells in pig hearts has been limited so far^8^.

Applying different transgenic reporter cell lines to track CM engraftment and function, recent NHP studies demonstrated the formation of electrically coupled and well-structured cardiac muscle islands from injected human embryonic stem cell (hESC)- and hiPSC-CMs in xenogeneic approaches^9–11^ and an equivalent allogeneic study using NHP iPSC-derived CMs^12^ as well. In a sub-acute MI model in macaques, Shiba et al. additionally reported a significant recovery of the ejection fraction (EF) and fractional shortening of respective hearts after the injection of NHP iPSC-CMs^12^. In more recent studies, the transplantation of hESC- and hiPSC-CMs into the infarcted heart in NHP models also suggested the substantial improvement of heart function 3 months after cell treatment^10,11^.

In terms of translation, the proposed need for relatively high cell doses for the regenerative treatment of a human heart, which is ~10 to 12 times larger compared to macaques^13^, creates substantial challenges for the (commercially viable) cell production and raises additional safety concerns in the clinical setting. Moreover, as of yet, the functional data provided by the NHP studies have to be considered preliminary in view of the small numbers of treated animals. It is also poorly understood whether the functional heart recovery depends on the contractile properties of the applied CMs and whether different reporter transgenes used in previous studies impact on the results. The transplantation of PSC-CM into NHP hearts has also been associated with adverse events: ventricular arrhythmias of different characteristics were observed in several cases and persisted throughout the observation period^9,11,12^, requesting further investigations before the clinical translation.

Here, we report the treatment of MI-damaged cynomolgus monkey (Macaca fascicularis) hearts with hiPSC-derived CMs. Applying a fundamentally clinically applicable and commercially viable approach, we have transplanted CM aggregates (hiCMAs) produced by scalable, chemically defined suspension culture technology^2,3^. This approach aims at testing whether the aggregate delivery, which remains compatible with the simple and flexible administration via intramyocardial injection, promotes the robust retention, survival and engraftment of transplanted cells, while reducing the applied cell dose compared to single-cell CMs recently tested in comparable models^9,11^. We demonstrate that the application of a well-characterized cell product, typically consisting of ~95–98% of ventricular-like CMs integrated into aggregates of ~200 µm average diameter, leads to the formation of substantial human cardiac grafts despite applying relatively low cell doses, that is ~5–7 × 10^7^ CMs per NHP heart. Our data suggest a remarkable improvement of the left ventricular ejection fraction (LVEF) of hiCMA-injected hearts compared to infarct-only controls. Notably, a parallel in vitro potency assay suggests that the in vivo recovery of heart function correlates with contractile forces and the transcriptional profiles of iPSC clone-dependent properties of the applied CMs. Molecular and cellular investigations revealed unexpected reporter transgene-related effects on cardiac gene expression as well as severely modulated contractile-, electrophysiological- and other properties of CMs derived from respective genetically engineered iPSC clones. In our NHP model, we have also observed and characterized cardiac arrhythmias of varying types, frequency and duration after MI induction and subsequent cell transplantation. Equivalent to the functional recovery data, the observed arrhythmias showed substantial iPSC clone/ transgene-dependent differences regarding their characteristics and severity. Generally, the arrhythmias were not life-threatening and gradually decreased over the 3 months observation period, in analogy to similar previous studies^11^.

Together, despite typical limitations such as the limited number of animals inherent to the NHP model, the study reveals robust proof-of-concept for our hiCMA-based treatment strategy to rescue damaged hearts, thereby providing a realistic option for clinical trials in the near future.

## Results

### High cardiomyocyte purity in cardiac aggregates is maintained after prolonged suspension culture

**Fig. 1:**
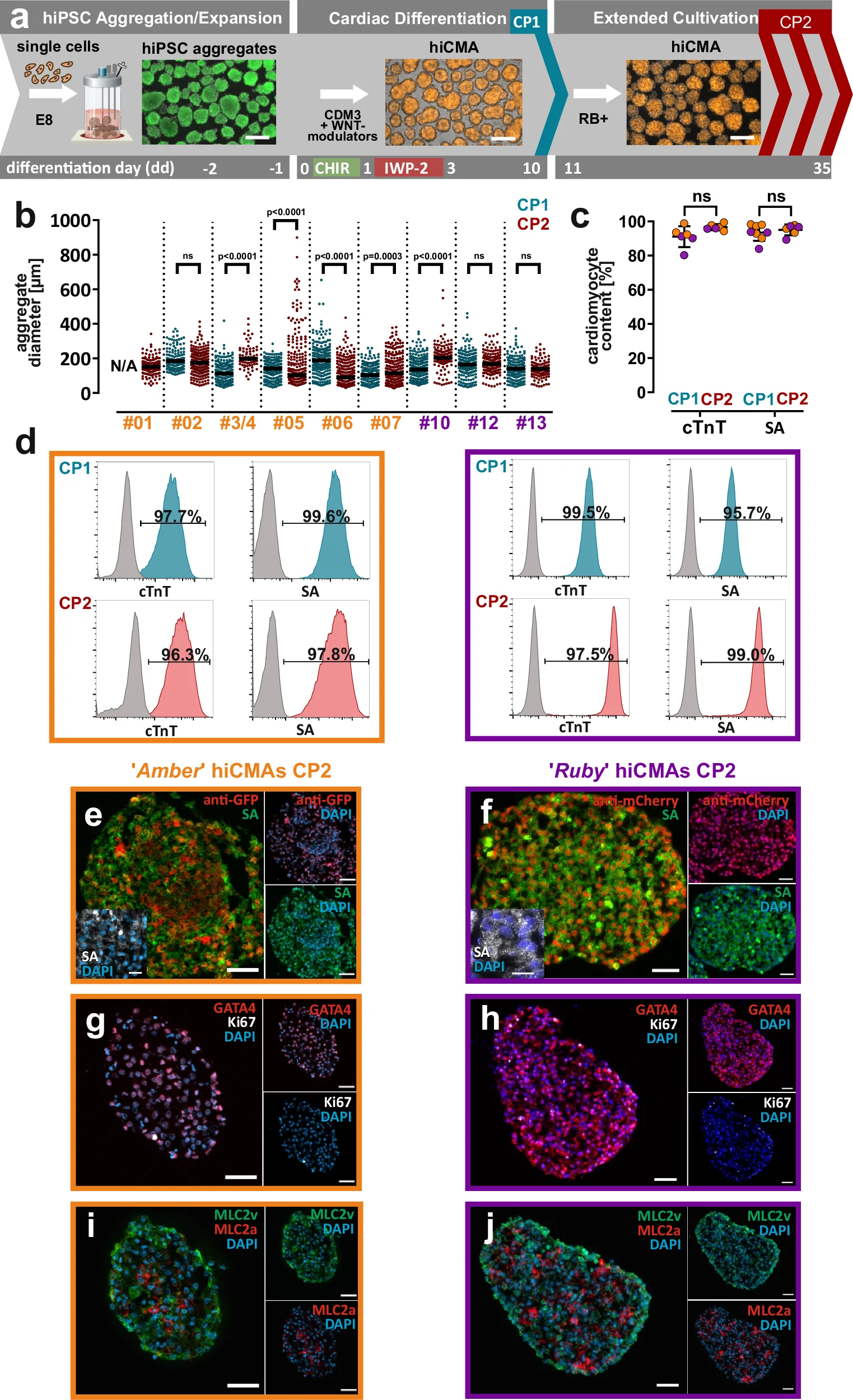
hiPSC-derived enriched cardiac aggregates for transplantation studies can be produced and maintained under defined and scalable culture conditions. **a** Viable hiPSC aggregates formed within 2 days after inoculation of a hiPSC suspension (fluorescence microscopy (FM) of calcein stain). For cardiac differentiation, WNT-modulators are supplemented; from dd11, RB+ Medium is used for extended hiCMA cultivation. On process Check Point 1 (CP1; end of differentiation) and CP2 (dd18–dd34, time point of transplantation) cardiomyocyte aggregates (hiCMAs) were analysed (FM picture of RedStar expression in “*Ruby*” hiCMAs; Scale bars: 100 µm). **b** Independently differentiated hiCMA batches showed similar aggregate diameters on CP1 and CP2, respectively (dots = individual aggregates; bars = resulting mean diameter); #01–#07 “*Amber*” hiCMAs; #10–#13 “*Ruby*” hiCMAs; ns not significant, or *p* value for one-way ANOVA with Bonferroni’s multiple comparison test. Individual batches for transplantation experiments are displayed with *n* = 69 to 256 aggregates analysed. **c** Flow cytometry indicated a typical CM content of ~95–98% on CP1 and CP2, respectively, for cardiac troponin T (cTnT) and α-sarcomeric actinin (SA); “*Amber*” hiCMAs (orange data points); “*Ruby*” hiCMAs (purple data points); Mean ± SD for *n* = 6; ns not significant for one-way ANOVA with Bonferroni’s multiple comparison test. **d** Representative flow cytometry data show high levels of cardiac marker expression in CMs derived from “*Amber*” (left box) and “*Ruby*” (right box) iPSCs (coloured) compared to controls (grey), both for CP1 and CP2. **e**–**j** Immunofluorescence (IF) analysis of cryosections of representative “*Amber*” (**e**, **g**, **i**) and “*Ruby*” (**f**, **h**, **j**) hiCMAs from CP2 for expression of α-sarcomeric actinin (SA), and the transgenes Venus_nucmem_ and RedStar_nucmem_ (stained with cross-reacting anti-GFP and anti-mCherry mAbs, respectively) in (**e**, **f**). **g**, **h** IF staining showed comparable expression of cardiac GATA4, and Ki-67 (Ki67) in “*Amber*” and “*Ruby*” hiCMAs. **i**, **j** Cryosections of representative hiCMAs show expression of ventricular and atrial myosin light chain (MLC2v and MLC2a). Nuclei stained with DAPI (blue). Scale bars: 100 µm; in the insets in (**e**, **f**): 20 µm. Representative images for a total of 6 “*Amber*” and 3 Ruby hiCMA batches. Source data are provided as a Source Data file. The “Single cells” and the “Bioreactor” illustrations incorporated in this figure (**a**) were created by D. Kleimenhagen.

We have recently established advanced cardiomyogenic differentiation of hPSCs in chemically defined suspension culture, enabling the robust induction of ventricular-like CMs at ~95–98% purity for numerous independent cell lines^2^. The schematic in Fig. 1a outlines this differentiation strategy, whereby the first process assessment was performed on “checkpoint 1” (CP1). However, here we extended this protocol by an additional cultivation period of the resulting cardiac aggregates (hiCMA) in stirred suspension culture for up to 5 weeks, that is until “checkpoint 2” (CP2; dd18–dd34). Notably, hiCMAs derived from two hiPSC-reporter cell lines were used in this study, MHHi0001-A-11 “*Amber*” and MHHi0001-A-5 “*Ruby*”, which represent subclones of the same parental cell line^14^ that are equipped with different reporter transgenes.

Besides some inter-experimental variability in the average diameter of hiCMAs (which was around ~200 µm; black bars in Fig. 1b) and the respective aggregate size distribution spread (indicated by individual dots in Fig. 1b), no apparent differences between CP1 versus CP2 samples were observed. Comparison of corresponding hiCMA samples on CP1 versus CP2 by flow cytometry specific to sarcomeric markers α-sarcomeric actinin (SA), and cardiac troponin T (cTnT) revealed no apparent difference in the CM content, which was typically >95% across numerous independent process runs (Fig. 1c). Exemplary data for individual differentiation experiments shows equally high expression levels for cTnT and SA at CP1 and CP2 for both “*Amber*”- and Ruby-derived CMs (Fig. 1d). This suggests the successful long-term maintenance of hiCMAs without apparent changes in the key cellular and physical properties of the aggregate cultures in our stirred suspension approach for both cell lines.

Individual hiCMAs were round-shaped and showed the expected nuclear localization of the reporter transgenes Venus^15^ and RedStar^16^ demonstrated by immunostaining with anti-GFP and anti-mCherry antibodies, respectively (Figs. 1e, f and S1). Immunostaining of hiCMA sections at CP1 for GATA4 and Ki67 showed the expression of an early cardiac marker and an active cell cycle, suggesting an expected “fetal-like” status (Fig. S1c, d). At CP2, immunostaining for SA demonstrated homogenous distribution of iPSC-derived CMs without any particular cell alignment, but with a cross-striated pattern showing sarcomeric structures (Fig. 1e, f, see insets). Besides some remaining expression of GATA4 and Ki67, the aggregates of both cell lines contained mostly MLV2v^pos^ cells as well as a portion of MLC2v/MLC2a double-positive cells (Fig. 1i, j).

Patch Clamp analysis revealed an expected early ventricular-like phenotype of hiCMA-derived CMs from both lines, “*Amber*” and “*Ruby*” (Fig. S1g). However, significant reporter line-dependent differences regarding specific electrophysiological properties were observed, including reduced action potential (AP) amplitude, upstroke velocity, and maximum sodium current density in the “*Ruby*” CMs (Fig. S1h–n).

hiCMAs showed a contractile phenotype in vitro (Supplementary Video 1) and calcium transients as indicated by the fluorescence reporter GCaMP6f^14^ in case of “*Ruby*” hiCMAs (Supplementary Video 2). Following preliminary experiments confirming that aggregates remained intact and viable after passing through a 25G needle (Fig. S1o), hiCMAs were directly used at CP2—without any dissociation or other downstream processing steps—for transplantation into the heart in our NHP model of MI.

### In vitro potency assessment reveals differences in contractile force development of hiCMA-derived CMs from the two hiPSC reporter lines

**Fig. 2:**
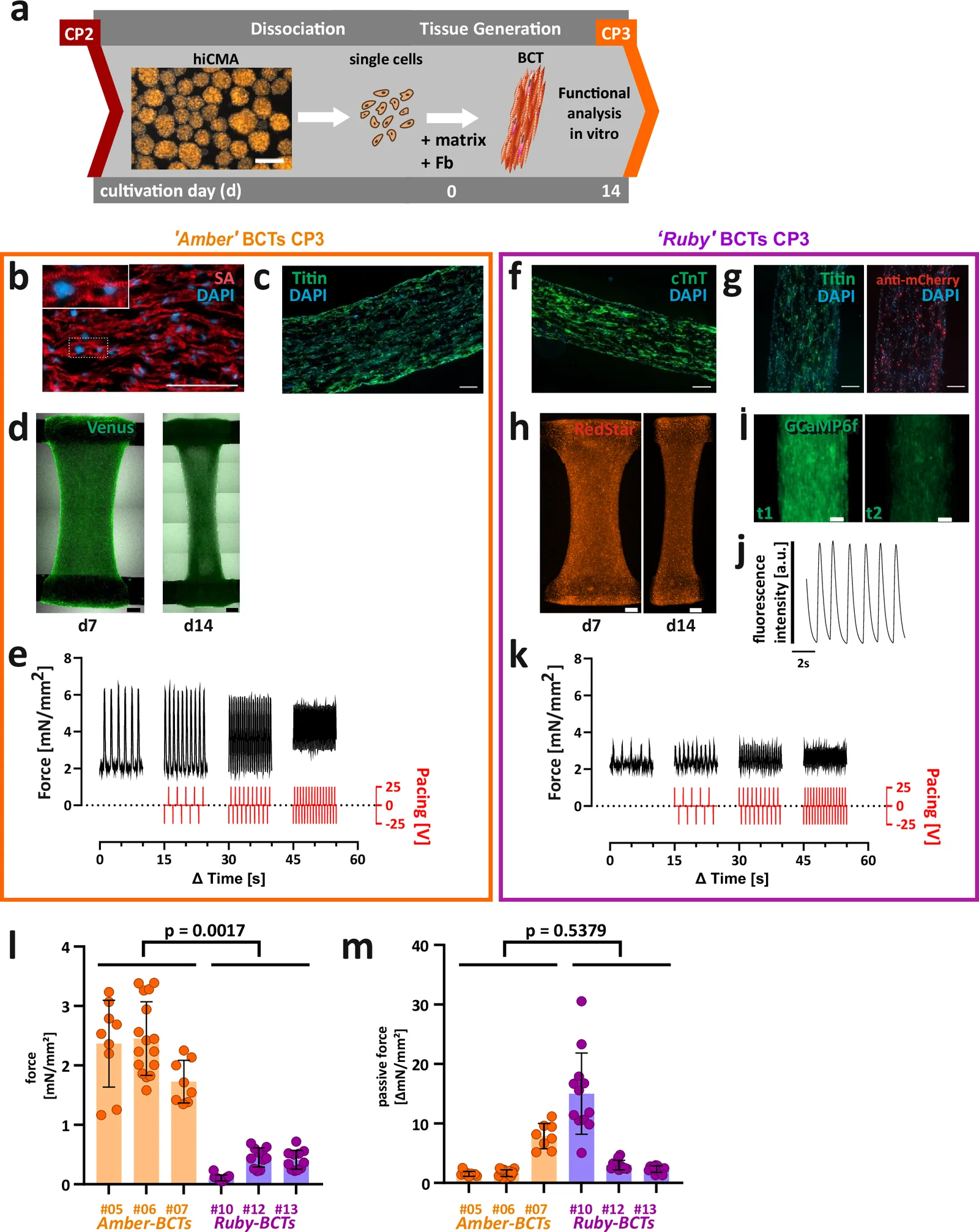
In vitro formation of homogenous bioartificial cardiac tissue confirms plasticity and functionality of hiCMAs used for transplantation. **a** Bioartificial cardiac tissue (BCT) produced from batches of hiCMAs at the time of transplantation (CP2) was used to assess the ability of the “*Amber*” (**b**–**e**), or “*Ruby*” (**f**–**k**) hiCMAs to form structured myocardium in vitro. After 14 d (CP3), BCTs were used for analysis of CM contractility of individual batches. **b**, **c** IF staining of cryosections of d14 BCT from “*Amber*” for α-sarcomeric actinin (SA, red) and titin (green) showed elongated and aligned CMs and a cross-striated staining pattern of sarcomeres (**b**, see inset). Nuclei were stained with DAPI (blue), scale bar 100 µm. **d** “*Amber*” BCTs expressing the Venus_nucmem_ transgene show viable CMs with tissue remodelling on d7 and d14; scale bars 500 µm. **e** Exemplary force measurement data from an “*Amber*”-BCT, shown for spontaneous contractions and during electrical pacing at 1, 2, and 3 Hz. **f**, **g** BCTs from “*Ruby*” (expressing the RedStar_nucmem_ and GCaMP6f transgenes) show aligned cTnT^pos^ and Titin^pos^ CMs and (**h**) tissue remodeling on d7 and d14; scale bars 500 µm. **i** In “*Ruby*”-BCTs, the calcium sensor GCaMP6f allowed monitoring of intracellular calcium in BCTs (still images from one BCT video, scale bar 200 µm), enabling video-optical analysis of calcium-dependent fluorescence oscillations (**j**) (a. u. arbitrary units). **k** Exemplary force measurement data from a “*Ruby*”-BCT for spontaneous contractions and during electrical pacing at 1, 2, and 3 Hz. **l** On d14, maximum active forces of BCTs from individual hiCMA batches (normalized to the cross-sectional area) show large variations of contractility in vitro with significantly higher forces for “*Amber*”-BCTs vs. “*Ruby*”-BCTs (2.2 ± 0.4 mN/mm^2^ vs. 0.3 ± 0.2 mN/mm^2^ for “*Ruby*” BCTs; Mean ± SD for *n* = 3 experiments per group with *n* = 8–16 BCTs each; *p* = 0.0017 for two-tailed nested *t*-test). **m** Normalized Passive forces indicating tissue stiffness tended to be higher in “*Ruby*”-BCTs than in “*Amber*”-BCTs with values up to 15 ± 6.8 mN/mm^2^ (Mean ± SD, *n* = 12 BCTs, *p* = 0.5379 for two-tailed nested *t*-test) for the least contractile “*Ruby*”-BCTs from hiCMA batch #10. Source data are provided as a Source Data file. The “BCT” illustration incorporated in this figure (**a**) was created by D. Kleimenhagen.

In parallel to each cardiac cell transplantation experiment in vivo, the respective batches of hiCMAs from CP2 were also tested for their potential to form contractile bioartificial cardiac tissue (BCTs^17^) in vitro. CMs derived from both iPSC lines formed spontaneously contracting tissues (termed “*Amber*” BCTs and “*Ruby*” BCTs, respectively), which were analyzed 14 days after tissue production (CP3; schematic outline in Fig. 2a). Both “*Amber*” and “*Ruby*” BCTs contained aligned CMs as exemplified for SA, titin and cTnT immunostaining (Fig. 2b, c, f, g) and typical morphological changes i.e., tissue thinning due to matrix remodeling over time (Fig. 2d, h). For “*Ruby*” BCTs, expressing nuclear RedStar (detectable with an anti-mCherry antibody; Fig. 2g) and the additional transgene GCaMP6f, video-optical assessment of calcium concentrations could be performed and oscillations reflected synchronized tissue contractions (Fig. 2i, j). Contraction forces were measured on d14 as exemplified in Fig. 2e, k for spontaneously beating BCTs, as well as after electrical stimulation at different frequencies, revealing a negative or flat force-frequency-relationship for “*Amber*” and “*Ruby*” BCTs, respectively (Fig. S2a, b).

Despite BCTs being produced by highly standardized conditions from individual hiCMAs batches with similar CM content, we observed some degree of batch-dependent variations, but particularly significant hiPSC line-related differences regarding respective levels of active force development (Fig. 2l). While active forces were significantly higher for “*Amber*” BCTs (2.2 ± 0.4 mN/mm^2^ vs. 0.3 ± 0.2 mN/mm^2^ for “*Ruby*” BCTs; Mean ± SD for 3 experiments with *n* = 8–16 BCTs each; *p* < 0.05 for nested *t*-test), passive forces (indicating tissue stiffness) were somewhat higher for “*Ruby*”-BCTs with values up to 15 ± 6.8 mN/mm^2^ (Mean ± SD, *n* = 12 BCTs) (Fig. 2m).

Such differences in active forces for “*Amber*” and “*Ruby*” BCTs were consistent in two additional, independent clones representing the same transgene modifications specific for each reporter cell line (Fig. S2c, d). These results prompted us to further investigate the influence of the different transgenes in comparative analyses including: “*Amber*” BCTs (expressing Venus only), “*Ruby*” BCTs (expressing both RedStar and GCaMP), BCTs derived from additional mono-transgenic iPSC lines derived from “*Phoenix*” (i.e., the non-transgenic maternal iPSC line of “*Amber*” and “*Ruby*”) representative of the respective individual transgenes (i.e., a nuclear RedStar only and a GCaMP only expressing line) and non-transgenic controls as well (i.e., “*Phoenix*”). Interestingly, BCTs generated from “*Ruby*”- and “*RedStar*”-derived CMs showed significantly reduced contractile forces in this comparative assay (Fig. S2e), suggesting that the presence of the nuclear RedStar transgene expressed in these cells is the underlying cause of the “low contraction force” phenotype.

### Infarction-related loss of myocardial function in the NHP model can be correlated with serum levels of troponin T as a reliable biomarker

**Fig. 3:**
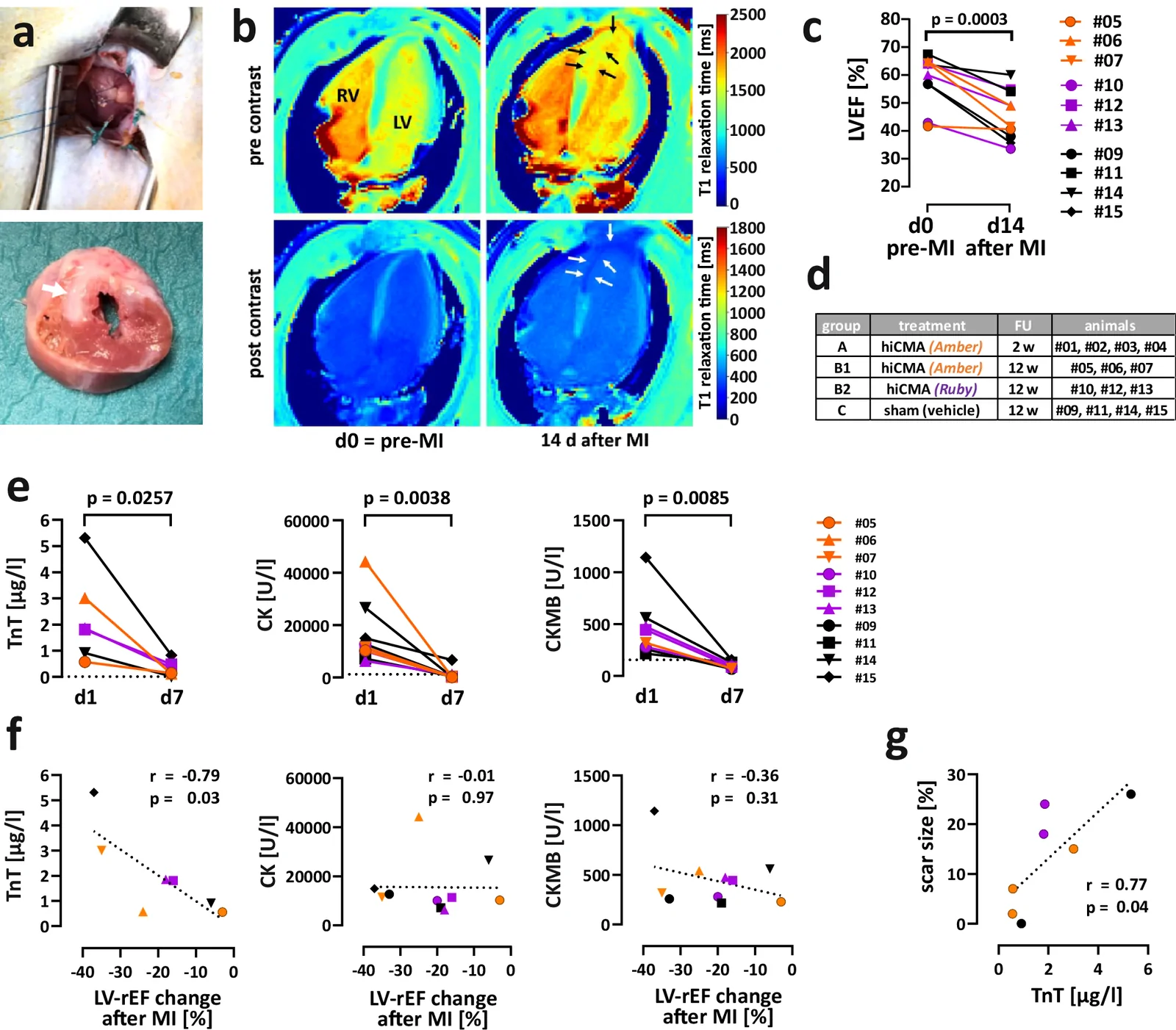
Induction of myocardial infarction in cynomolgus monkeys. **a** Myocardial infarction (MI) was induced by permanent coronary artery ligation after exposing the heart via left lateral thoracotomy under general anaesthesia. After explantation, macroscopic assessment of a short-axis heart slice of an untreated animal (#09), 3 months after MI, indicates extensive scar formation (white arrow). **b** Representative MRI T1 relaxation maps of the myocardium from healthy monkey #09 before MI (pre-MI) and 14 days after infarction demonstrate localised changes in tissue composition. Increased native (pre-contrast, top) and markedly reduced post-contrast (bottom) T1 relaxation times delineate the fibrotic scar (black and white arrows) located in the septal and apical regions of the myocardium. **c** Cardiac MRI demonstrated a wide range of pre-MI LVEF and LVEF on d14 after MI for individual animals; with overall reduction of LVEF after MI being significant (*p* = 0.0003, two-tailed paired *t*-test; *n* = 10), but with very small changes in animals #05 and #14 (see Table 1 for individual data). **d** Infarcted animals were assigned to the treatment groups A and B1 (treatment with hiCMA “*Amber*”), group B2 (treatment with hiCMA “*Ruby*”), and sham control group C (injection of vehicle (PBS), black). **e** Serum levels of infarction markers cardiac troponin T (cTnT, *n* = 6 animals), creatine kinase (CK, *n* = 10), and creatine kinase MB isoenzyme (CK-MB, *n* = 8) were determined 1 and 7 days after MI in animal groups B1, B2, and C (*p* values for two-tailed paired *t*-test). Dotted lines represent mean levels measured in healthy control animals unrelated to this study (*n* = 6). **f** Correlation analyses of serum levels of cTnT, CK, and CK-MB on d1 after coronary artery ligation and functional impairment of cardiac contractility on day 14 assessed by MRI (LV-rEF change after MI [%] = (LV-rEF d14 – LV-rEF d0) × 100) for groups B1, B2, and C (Pearson correlation coefficient (*r*) and *p* value for two-tailed analysis with *n* = 7–10). **g** Correlation analyses of serum levels of cTnT and scar size assessed by MRI for groups B1, B2, and C (Pearson correlation coefficient (*r*) and *p* value for two-tailed analysis with *n* = 7). Source data are provided as a Source Data file.

An MI model in cynomolgus monkeys (*n* = 15) was established by permanent coronary artery ligation under general anesthesia (Fig. 3a). Directly following the ligation, the affected territory appeared pale and intra-operative transoesophageal echocardiography showed dyskinetic myocardial regions (data not shown). Untreated MI led to extensive myocardial scar formation after 3 months (Fig. 3a).

On d14 after MI, monkey hearts were assessed by cardiac MRI, showing loss of contractile myocardium in left ventricular and septal areas near the apex (Figs. 3b and S3a, b). Functional parameters showed a significant increase of left ventricular end-systolic volume (LVESV) after MI (7.8 ± 2.4 ml vs. 5.6 ± 1.3 ml before MI), while the end-diastolic volume (LVEDV) did not change significantly (Fig. S3c; see Table 1 for individual data). Consequently, LVEF was reduced significantly (Fig. 3c), as was demonstrated by two independent observers with good inter-observer agreement (Fig. S3d). Cardiac MRI demonstrated a wide range of pre-MI LVEF and LVEF on d14 after MI for individual animals with very small changes in animals #05 and #14 (Table 1). Due to the large variance of pre-MI LVEF, relative LVEF values normalized to pre-MI values (Fig. S3c) were used for comparative analyses. Following MI, individual animals were treated either by the intramyocardial injection of iPSC line-specific hiCMAs in groups A, B1, and B2 or Dulbecco’s PBS for the sham control group C (Fig. 3d).

**Table 1:** Data for individual macaques in MRI study

				Pre-infarction				Post-infarction baseline				2 weeks after treatment				12 weeks after treatment			
Animal #	Sex	Age (years)	Weight (kg)	LVEDV (ml)	LVESV (ml)	LVEF (%)	LV-rEF	LVEDV (ml)	LVESV (ml)	LVEF (%)	LV-rEF	LVEDV (ml)	LVESV (ml)	LVEF (%)	LV-rEF	LVEDV (ml)	LVESV (ml)	LVEF (%)	LV-rEF
Group B1—hiCMA iPSC1 transplantation																			
#05^a^	M	6.1	11.7	11.7	6.8	41.7	1.00	12.4	7.4	40.6	0.97	10.1	5.9	41.6	1.00	11.6	6.6	43.0	1.03
#06	M	7.9	10.5	15.1	5.3	64.8	1.00	16.0	8.2	49.0	0.76	14.1	5.4	61.6	0.95	15.6	6.6	57.8	0.89
#07	M	7.5	8.8	15.6	5.6	64.3	1.00	16.6	9.7	41.6	0.65	12.3	7.1	42.4	0.66	13.4	6.2	54.1	0.84
Group B2—hiCMA iPSC2 transplantation																			
#10^a^	M	7.3	11.8	9.8	5.6	42.9	1.00	8.3	5.5	33.6	0.80	8.7	5.5	37.1	0.88	7.8	5.1	36.0	0.84
#12	M	7.3	12.3	12.0	4.5	64.0	1.00	12.7	5.9	54.9	0.84	12.3	6.0	52.0	0.81	14.5	6.6	55.3	0.87
#13	M	7.3	9.0	16.4	6.6	60.8	1.00	15.3	7.8	49.5	0.82	14.2	6.8	51.1	0.87	16.3	8.4	48.7	0.81
Group C—Sham control																			
#09	M	7.2	9.8	17.8	7.7	56.8	1.00	20.1	12.4	38.1	0.67	18.2	11.3	37.8	0.67	19.3	11.5	40.5	0.71
#11	M	3.7	7.8	10.9	3.5	67.5	1.00	10.6	4.9	54.1	0.81	9.8	4.0	59.3	0.88	11.0	5.3	51.6	0.77
#14	M	7.7	10.5	12.3	4.5	63.7	1.00	15.1	6.0	60.1	0.94	15.4	5.4	64.8	1.02	14.2	5.8	59.5	0.93
#15	M	7.6	9.3	13.9	6.0	56.7	1.00	15.4	9.9	35.7	0.63	16.3	10.1	38.2	0.67	18.7	11.1	40.8	0.72

MRI data are given as mean from two independent observers.

*LVEDV* left ventricular end-diastolic volume, *LVESV* left ventricular end-systolic volume, *LVEF* left ventricular ejection fraction, *LV-rEF* relative left ventricular ejection fraction.

^a^Lower absolute values for MRI parameters due to analysis with real time mode.

Serum level analyses for clinically used infarction biomarkers troponin T (TnT), creatine kinase (CK), and creatine kinase MB isoenzyme (CK-MB) were performed after MI and in healthy controls. On d1 after ligation, all markers showed significantly elevated levels and returned back to control levels on d7 (Fig. 3e). Then, d1 biomarker levels were assessed in relation to the decrease in LVEF observed by MRI (Fig. 3f and Table 1). While CK and CK-MB levels—as well as their ratio—were affected in our animal model, only levels of troponin T directly correlated with the LV-rEF change after MI (Figs. 3f and S3e) and scar size (Fig. 3g), thus only troponin T seems to represent a reliable biomarker for the extent of infarction-related loss of contractile function.

### Human iCMAs form large grafts structurally integrated into the primate myocardium

**Fig. 4:**
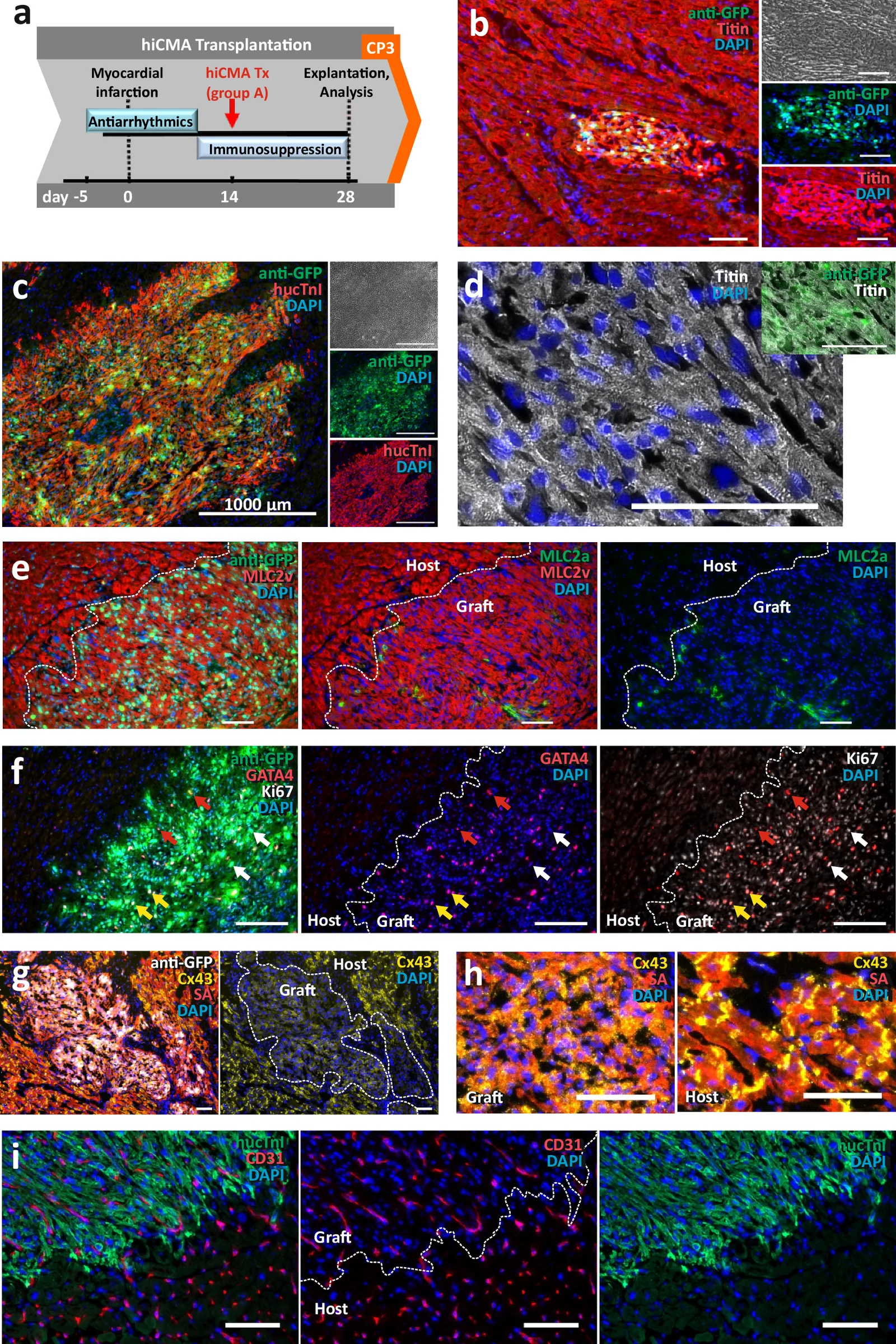
Human iPSC-derived cardiomyocyte aggregates (hiCMAs) integrate into the infarcted non-human primate heart and form large matured grafts within 14 days. **a** Experimental outline of short-term group A hiCMA transplantation 14 d after MI and heart tissue sampling and analyses after another 14 d on d28 (CP3). Within group A only “*Amber*” hiCMAs were injected. **b** Small human CM graft presumably originating from a single hiCMA, identified by IF in the host myocardium with an anti GFP antibody (staining the yellow GFP variant Venus, green) and an antibody against Titin (red). **c** IF also identified large grafts of human CMs in the host myocardium with a species-specific antibody against human cTnI (red) and GFP (staining Venus, green). **d** Venus_nucmem_^pos^ hiCMA grafts (green) expressing the cardiac marker titin (white) contain aligned, elongated cells with a cross-striated staining pattern that reflects sarcomeric organization. **e** Characterization of CM subtype marker expression by IF with shows predominantly MLC2v^pos^ CMs (red) in the graft area identified with the anti-GFP mAb (green, left panel), boundary marked with a dotted line. Middle and right panels: Co-staining for MLC2v^pos^ CMs (red) and MLC2a^pos^ CMs (displayed in green). **f** In human grafts, identified with the anti-GFP antibody (left panel) and marked with a dotted line (middle and right), IF detected GATA^pos^ CMs (red, red arrows), Ki67^pos^ cells (white, white arrows) and GATA^pos^/Ki67^pos^ CMs (yellow arrows). **g** Connexin 43 (CX43, yellow) is expressed in human grafts but at a lower level than in the surrounding NHP tissue. **h** CX43 is less organized in human grafts (left) than in the host myocardium (right). **i** In human grafts, identified with the antibody against human cTnI (green), IF detected CD31^pos^ endothelial cells (red). All nuclei were stained with DAPI (blue); Scale bars 100 µm in (**b**); 1000 µm in (**c**); 100 µm in (**d**–**i**). Representative images for a total of 4 cell transplantation experiments with “*Amber*” hiCMAs.

Intramyocardial injection of hiCMAs was performed 14 days after MI, reflecting a sub-acute disease state; immunosuppression was initiated 5 days before cell administration (Figs. 4a and S4a). In a first set of experiments with hiCMAs from “*Amber*” iPSCs (group A, *n* = 4), we focused on the analysis of short-term engraftment 2 weeks after hiCMA injection (i.e., CP3, Figs. 4a and S4b). In 4 out of 4 NHP hearts, donor cells were detected: human CMs were identified by staining with an anti-GFP antibody (staining the yellow GFP variant Venus). Grafts of small areas approximately equivalent to individual hiCMA dimensions (Fig. 4b) suggested that individual aggregates did not disintegrate during the injection procedure. These grafts showed structural integration between existing muscle fibers and typically showed an oval appearance (Fig. 4b), but were rather round-shaped when embedded in fat/connective tissue (not shown). Relatively large grafts were detected as well, indicating substantial cell survival and the in vivo fusion of individual hiCMAs (Fig. 4c). Human cell alignment was visible within the grafts, including clear cross-striation of the donor CMs (Fig. 4d); both aligned and less organized areas were observed. Human cells expressed the CM markers titin, cardiac troponin I (cTnI), SA (Fig. 4b–d, g–i), as well as cTnT (not shown). Already on day 14, the vast majority of grafted human cells solely expressed the CM subtype-specific marker MLC2v; only a minority expressed MLC2a (Fig. 4e).

CMs within the graft further expressed the cardiac transcription factor GATA4 at different levels. The number of Ki67-expressing cells was higher in the graft region compared to the surrounding host myocardium (Fig. 4f), including a subset of GATA4/Ki67 double-positive human CMs, indicating maintenance of an immature embryonic-like expression profile. Expression of connexin 43 (CX43) was detectable but significantly lower signals were observed in the grafted cells in comparison to the host myocardium (Fig. 4g). Moreover, CX43 expression was less organized in the grafts (Fig. 4h). Notably, within the graft regions, also CD31^pos^ endothelial cells (ECs) were abundant (Fig. 4i), indicating fast vascularization of the human graft during the initial post-transplantation period.

**Fig. 5:**
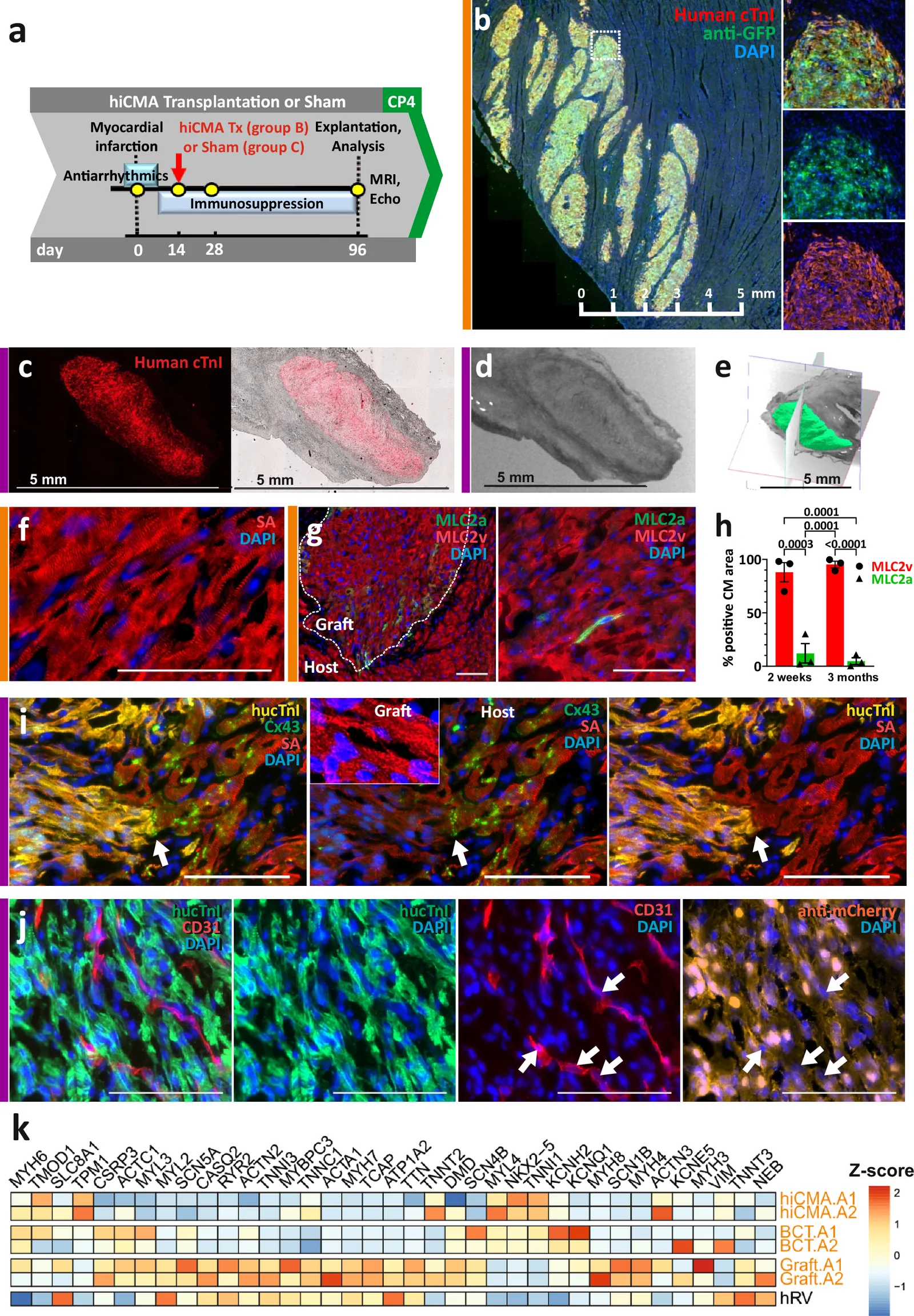
Three months after injection human iCMAs form large grafts structurally integrated into the infarcted non-human primate heart. **a** Long-term groups with “*Amber*” or “*Ruby*” hiCMA transplantation 14d after MI and heart tissue sampling and analyses on day 96 (3 months after cell transplantation, CP4). **b** Extensive grafts of human “*Amber*”-derived CMs in the host myocardium were immunostained for human-specific cTNI (red) and the yellow Venus_nucmem_ (green) and quantified by 2D-histomorphometry; here: grafts in animal #07 with a total area of 16 mm^2^; scale bar 5 mm. **c** Large islands of engrafted “Ruby”-derived CMs were confirmed by IF for human-specific cTNI (red; scale bars 5 mm) after **d** 3D imaging using a laboratory X-ray μ-CT (**d**, single plane corresponding to (**c**)); **e** 3D reconstruction for volumetric analysis showing a substantial “*Ruby*” graft in animal #13 with a volume of 9.2 mm^3^; scale bar 5 mm. **f** Anti sarcomeric actinin (SA) staining shows “*Amber*”-derived aligned, cross-striated human graft myocardium. **g** IF for MLC2v (red) and MLC2a (green) in the “*Amber*” iPS-derived human de novo myocardium showed small numbers of MLC2a^pos^ cells in clusters (left) or as single cells (right). **h** Quantification of MLC2v^pos^ CMs (red) and MLC2a^pos^ CMs (green) given as % positive CM area in the grafts after 2 weeks (2w, “*Amber*” only) and 3 months (3 m, “*Amber*” and “*Ruby*”). Mean ± SEM; *n* = 3 animals; *p* values for two-way ANOVA with Bonferroni’s multiple comparison test. **i** Anti CX43 staining suggests functional coupling of “*Ruby*”-CMs; human-specific cTNI (yellow), SA (red), CX43 (green). **j** The “*Ruby*” hiPSC-derived myocardium detected by human-specific cTNI, green, contains capillaries from host-derived (i.e., RedStar/mCherry^neg^, orange) endothelial cells (CD31^pos^, red, see white arrows). All nuclei were stained with DAPI (blue); Scale bars 5 mm in (**b**); 100 µm in (**c**–**f**). **k** RNASeq data reveals progressing CM maturation in “*Amber*”-derived human grafts three months after injection of hiCMAs in comparison to freshly produced hiCMAs and BCTs derived for “*Amber*” iPSCs. Color scale represents row-wise *Z*-score-scaled TPM values. Representative images for a total of 6 cell transplantation experiments with *n* = 3 animals each for “*Amber*”- and “*Ruby*”-hiCMAs. Source data are provided as a Source Data file.

Long-term survival and integration of hiCMAs after sub-acute MI in NHPs (group B animals) was also explored in another set of experiments with a follow-up time of 3 months (Fig. 5a) for both “*Amber*” hiCMAs (group B1, *n* = 3) and “*Ruby*” hiCMAs (group B2, *n* = 3) Fig. S4b). All injections were performed under visual control after re-thoracotomy to target the left-ventricular region apical to the coronary artery ligation (Fig. S5a).

On d96 after transplantation, human cells were detected in 6 out of 6 NHP hearts, confirming that the immunosuppression applied enabled the long-term survival of xenogeneic human iPSC-CMs. Grafts were located in particular outside the infarct area and mostly in apical segments of the left ventricle, including the apex itself (Figs. 5 and S5b).

To exemplarily quantify graft sizes, we used histomorphometry of 2D images of tissue sections after immunostaining (Fig. 5b, c), or 3D imaging of paraffin-embedded tissue samples using a laboratory X-ray μ-CT (computed tomography) (Figs. 5d, e and S5c, d). The substantial “*Amber*” grafts found in animal #07 had a visible area of 16 mm^2^ (Fig. 5b) and a total estimated graft volume 6–8 mm^3^. In the “*Ruby*” injected animal #12 a total graft volume of 5.6 mm^3^ was observed (data not shown) whereas in animal #13 a single graft volume of 9.2 mm^3^ (Fig. 5e) and a total graft volume was 17.2 mm^3^ was revealed. For both cell lines, CM density was similar in all grafts investigated 3 months after injection with a mean of 1795 ± 151 CM/mm^2^ (determined as nuclei of titin^+^ cells in the graft area, Fig. S5e). Based on these data, the estimated number of engrafted hiPSC-derived CMs were ~650,000 for “*Amber*” grafts found in animal #07, compared to 502,769 ± 56,956 and 1,539,729 ± 174,428 for “*Ruby*” grafts found in animals #12 and #13, respectively.

Depending on the muscle fiber orientation in the analyzed tissue slices, linear arrangement of CMs with distinct cross-striations was observed in “*Amber*”- and “*Ruby*” grafts (Fig. 5f, i). While already in d14 grafts, predominantly MLC2v expressing human CMs were detected, the number of MLC2a expressing human donor cells was further reduced after 3 months of engraftment, indicating progressive maturation of the transplanted cells (Fig. 5g, h). This was confirmed by analyses of RNASeq data that indicate progressing maturation of grafted “*Amber*”-derived human CMs 3 months after hiCMA injection compared to freshly differentiated “*Amber*”-CMs, “*Amber*”-BCTs and human right ventricular myocardium (Fig. 5k). As seen for “*Amber*”-CMs already after two weeks (Fig. 4g, h), anti CX43 staining suggested potential cell-cell coupling of individual host and graft CMs via gap junctions for “*Ruby*” CMs as well (Fig. 5i). Moreover, anti CD31/anti-mCherry co-staining provided evidence for a host-derived vascularization of the newly formed human myocardium from “*Ruby*” CMs (Fig. 5j); however, the endothelial cell density appeared to be lower in *Ruby’* grafts (Fig. S5f).

### hiCMA transplantation leads to substantial recovery of heart function after sub-acute myocardial infarction

Cardiac MRI was performed at four time points to assess myocardial function before and after treatment with a long-term follow-up for 3 months (Fig. S4a, b). In the experimental groups B1, B2, and C, a total of 10 animals underwent coronary artery ligation to induce MI as described above (Figs. 3d and S4b). The presence and extent of non-contractile scar tissue in NHP hearts was demonstrated by HE staining (Fig. S6a), confirming cardiac MRI analyses. Within the scar area, characteristic values of T1-relaxation times and an increased extracellular volume (ECV) of 80% showed fibrotic tissue changes in comparison to the remote region with an ECV of ~25%, while the scar tissue characteristics did not differ between the treated and untreated group (Fig. S6b). The size of the fibrotic area showed a significant correlation with the decrease of LV function (Fig. S6c).

Due to the large variance of pre-MI LVEF (Table 1), relative LVEF values normalized to pre-MI values were used to assess the effect of intramyocardial hiCMA injection. A subset of the animals was treated with hiCMA transplantation with “*Amber*” (group B1, *n* = 3) or “*Ruby*” (group B2, *n* = 3), while the remaining animals served as sham control group with re-thoracotomy and vehicle (Dulbecco’s PBS) injection (Fig. S4b).

An unbiased assessment of the combined treatment groups B1 + B2 (*n* = 6) compared to the sham control group C (*n* = 4) is shown in Fig. S6d with a trend for an increased LV-rEF after 3 months, however, the two animals with very small LVEF changes after MI (#05 and #14, Fig. 3c and Table 1) were identified as outliers preventing a meaningful analysis of functional improvement.

**Fig. 6:**
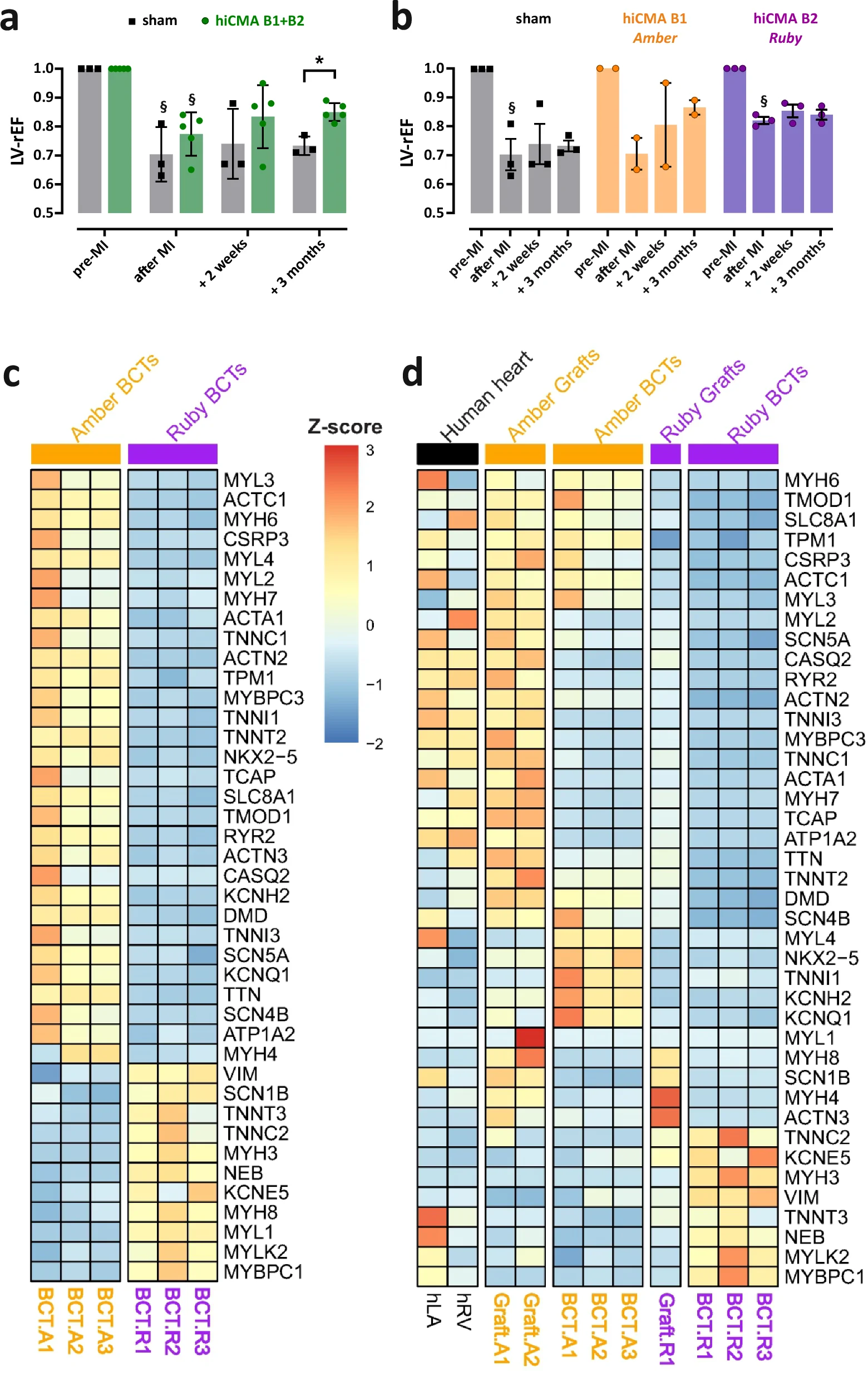
hiCMA transplantation results in substantial recovery of cardiac function, with cell line/transgene -specific differences in outcome corresponding to cardiac gene expression patterns, including key sarcomeric markers. **a** LVEF values normalized to pre-MI values (LV-rEF) were used to assess the outcome of individual hiCMA-treated animals. Only animals with a significant reduction of LVEF after MI were included (*p* < 0.05 for paired *t*-test vs pre-MI) i.e., the treatment groups B1 + B2 (green symbols) with *n* = 5 (#06, #07, #10, #12, #13) and the sham control group C (black symbols) with *n* = 3 (#09, #11, #15). Direct comparison showed a significantly increased LV-rEF with relative values of 0.85 ± 0.03 vs. 0.73 ± 0.03 for treated vs. sham-treated animals, respectively, after 3 months (§, *p* = 0.0026 and *p* = 0.0322, respectively, compared to respective pre-MI with paired *t*-test; *, adjusted *p* value = 0.007 for unpaired *t*-test with Holm-Šidák method and *n* = 3–5). **b** This data was re-analyzed for treatment groups B1 (#06, #07: orange bars & symbols) and B2 (#10, #12, #13; purple bars & symbols), separately. While the low number of treated animals did not allow achieving significance, a clear trend towards increased LV-rEF two weeks and 3 months after cell injection was observed after hiCMA treatment using hiCMA from “*Amber*” (treatment group B1), indicating substantial recovery of myocardial contractility after treatment. In contrast, no improvement was observed for hiCMA treatment using iPSC line “*Ruby*” (treatment group B2; §, *p* = 0.0041 compared to respective pre-MI with paired *t*-test); no significant differences between groups with unpaired *t*-test with Holm-Šidák method and *n* = 3). In addition, gene expression patterns were analysed for combined GO terms of cardiac muscle contraction and muscle filament sliding in BCTs and grafts derived from “*Ruby*” and “*Amber*” hiPS cell lines. **c** Comparison of BCTs from hiPSC lines “Ambe*r*” and “*Ruby*”; **d** Comparison of “*Amber*” and “*Ruby*” derived BCTs and grafts 3 months after hiCMA injection into NHP hearts and myocardial control samples from human left atrium (hLA) and right ventricle (hRV). Color scale represents row-wise *Z*-score-scaled TPM values. Source data are provided as a Source Data file.

Including only noticeably infarcted animals in Fig. 6a, the values for the relative LV-rEF confirmed a significant decrease of contractility after MI for the cell-treated group B (0.78 ± 0.08 vs. pre-MI (= 1); *p* < 0.05 for *n* = 5), and for the untreated group C animals (0.70 ± 0.10 vs. pre-MI (= 1); *p* < 0.05, for *n* = 3). Substantial recovery was observed for the cell-treated group B animals already on day 14 (relative LVEF of 0.83 ± 0.11), and more pronounced after 3 months with a relative LVEF of 0.85 ± 0.03, which is significantly higher than for the sham control animals (0.73 ± 0.03; *p* < 0.05 for *n* = 3).

Finally, a cell line-dependent analysis revealed that the observed functional recovery after hiCMA treatment was due to substantial improvement of LV-rEF in group B1 (Fig. 6b). Animals that received hiCMAs produced from the “*Amber*” reporter line showed a substantial recovery of heart function already on day 14 (relative LVEF 0.81 ± 0.21 vs. 0.71 ± 0.01 at baseline after MI); such observations were even more pronounced in animals terminated after 3 months (relative LVEF 0.87 ± 0.04). In contrast, no increase in LV-rEF was observed in animals that received hiCMAs derived from the dual reporter line “*Ruby*”. In individual animals, the extent of functional improvement (LV-rEF change after 3 months) seemed to correlate with the extent of initial loss of contractility 2 weeks after MI (Fig. S6e and Table 1). Interestingly, a slight recovery was observed also for sham treated animals after large impairment (> 30% LVEF reduction), but this was clearly outperformed by the treatment with “*Amber*” hiCMAs. In contrast, no measurable recovery was observed after treatment of infarcted animals (with ~20% LVEF reduction) using hiCMA from “*Ruby*”, potentially reflecting the poor in vitro contractility of the respective CM production batches assessed by the parallel BCT approach (Fig. 2l).

### Low contractility of “*Ruby*”-derivatives is associated with substantially downregulated expression of major cardiac sarcomere markers

To investigate these functional observations on the molecular level, differential gene expression patterns of representative “*Amber*”- versus “*Ruby*”-derived BCT samples were investigated. Gene ontology (GO) analysis revealed that the GO terms “cardiac muscle contraction” and “muscle filament sliding” were among the GO terms with the highest percentage of significantly regulated genes (Fig. S7a). Since a substantial number of genes is shared between these two GO terms, those were combined and the 41 differentially expressed markers are shown by heat map in Fig. 6c, d.

Notably, 30 of these genes were expressed at substantially lower levels in the “*Ruby*”-derived BCTs, including those encoding for major sarcomeric proteins in heart muscle that is: Myosin Light Chains (MYL) 2, 3, 4; Myosin Heavy Chains (MYH) 4, 6, 7; Actin Alpha Cardiac Muscle 1 (ACTC1), Actinin Alpha 2 (ACTN2), Troponins (TNN) C1, I1, I3, T2; Myosin-Binding Protein C3) and Titin (TTN), many of which are known to be involved in altered force generation and cardiomyopathies^18^. Moreover, Ryanodine receptor 2 (RYR2) encoding for one of the major components of a calcium channel that supplies calcium to cardiac muscle and the cardiac homeobox transcription factor NKX2-5, which plays a critical role in transcriptional regulation during cardiogenesis and human congenital heart diseases^17,19,20^, were, besides other factors, also downregulated in “*Ruby*”-derived BCTs.

In addition to the BCTs, we further aligned the expression of these 41 marker genes to human cardiac tissue grafts isolated from NHP heart sections at 3 months post transplantation (including “*Amber*”*-* and “*Ruby*”-derived samples) and to native adult human heart muscle control samples as well (displayed in the heat map in Fig. 6d). Notably, this comparison suggests that the differential BCT expression patterns where partially also reflected in the respective grafts and, importantly, that the “*Amber*”-derived grafts seem to better reflect gene expression in the adult human heart compared to a “*Ruby*”-derived sample.

Together, this analysis highlights that “*Ruby*”-derived cardiac tissue expresses—in vitro and in vivo—substantially lower levels of key cardiac markers known to be involved in proper contractile function, as compared to “*Amber*”-derived samples. This correlates with the significantly higher contractile forces of “*Amber*”-BCTs (Figs. 2l and S2d, e) and, by tendency, an improved heart function in damaged NHP hearts after transplantation of “*Amber*”-hiCMAs, which was not observed after the transplantation of “*Ruby*”-hiCMAs (Figs. 6a, b and S6e and Table 1).

To explore potential paracrine effects of the grafts, we have analyzed the differential expression of secretion-associated genes (GO terms GO:0046903, GO:0032940, GO:0046887, GO:0050663, GO:0070278, GO:0002793) in “*Amber*” vs. “*Ruby*” BCTs. The heatmap in Fig. S7b shows the detected 53 differentially expressed genes that were also among the 613 genes that have recently been associated with post-myocardial infarction responses^21^. Among them, 12 genes encoding for secreted factors (highlighted in green letters; including IL6, LIF, EDN1, SCF2, WNT5A, and others) were upregulated in “*Ruby*” and 6 (i.e., NPPB, APLN, APOA1, VEFGA, IL33, and AGT) in the respective “*Amber*” samples. Since numerous of these factors, such as Vascular endothelial growth factor (VEGF)-A, Apelin (APLN), Apolipoprotein A1 (APOA1), and Interleukin 33, have been associated with cardio-protection or heart function improvement^22–25^, we cannot exclude paracrine effects resulting from our hiCMA injection.

### hiCMA transplantation is associated with a transient increase in ventricular arrhythmias

**Fig. 7:**
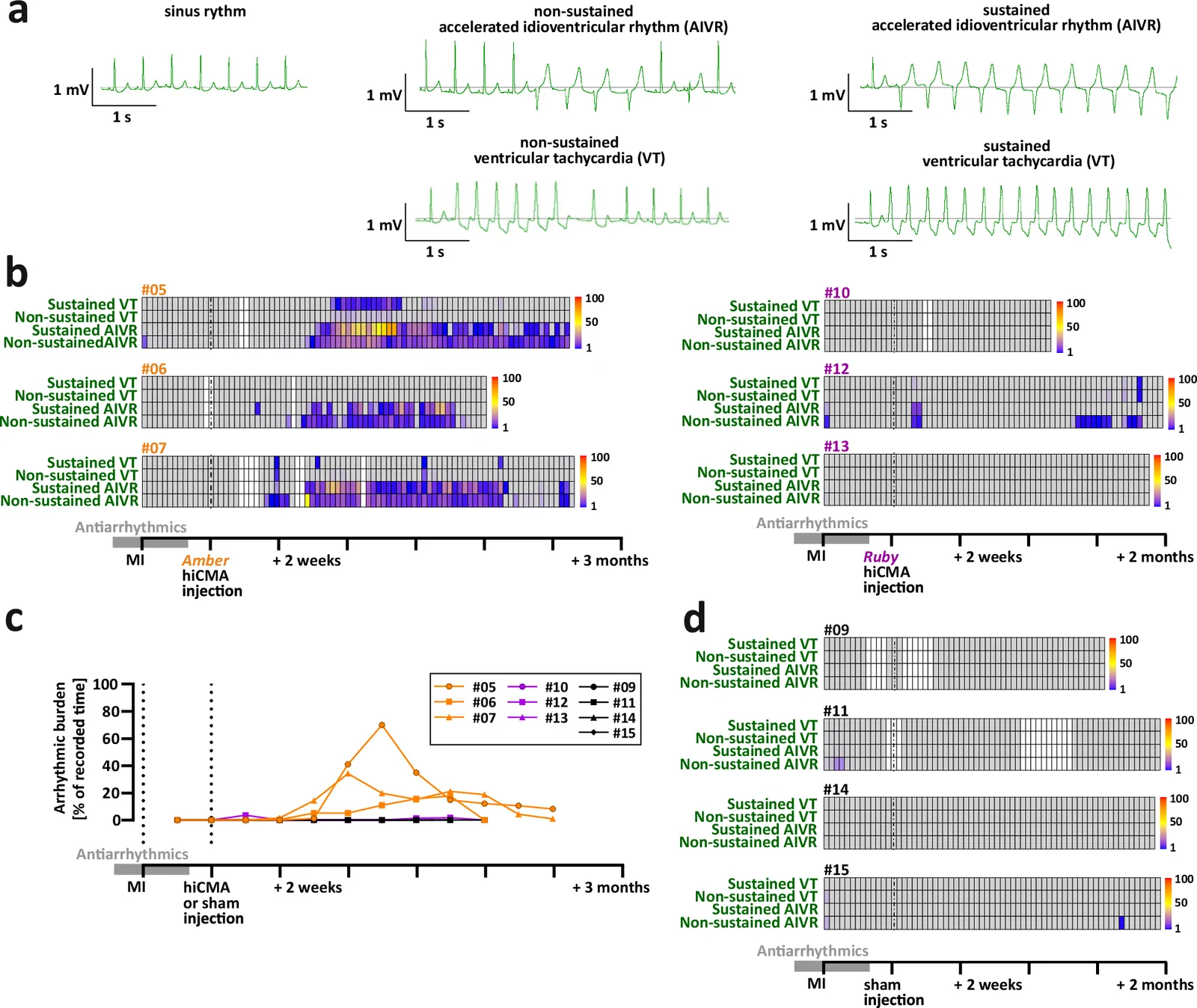
hiCMA transplantation is associated with transient graft-associated arrhythmias that substantially decline over time. **a** Telemetric ECG recording allowed for the detection of various types of arrhythmias, including non-sustained and sustained accelerated idioventricular rhythm (AIVR) as well as non-sustained and sustained ventricular tachycardia (VT) (see original traces). **b** Quantitative analyses of VTs and AIVRs revealed the duration per day (% of the recorded time) for each type of arrhythmia, shown as heat maps (white: no data acquired due to technical issues, including premature end of recording due to reduced battery lifespan); grey: data acquired but no arrhythmias. After hiCMA transplantation, a significantly higher burden of arrhythmia was found for individual animals in group B1 (#05, #06, #07; “*Amber*”) compared to B2 (#10, #12, #13; “*Ruby*”) over time. **c** Summarized data for the ventricular arrhythmia burden (VA, i.e., VT and AIVR) per week for individual animals after hiCMA transplantation (#05, #06, #07, “*Amber*” in orange; #10, #12, #13, “*Ruby*” in purple) compared to untreated control animals (#09, #11, #14, #15 in black). **d** Control animals from the sham-treated group C (#09, #11, #14, #15) showed none or a very short duration of each type of arrhythmia per day. Source data are provided as a Source Data file.

Telemetric ECG recording was performed to assess adverse effects of cell application, i.e., the occurrence of arrhythmias as described in the literature^9,11^. All animals received an initial anti-arrhythmic medication in the peri-infarct period with amiodarone and metoprolol (for 14 and 11 days, respectively). Original ECG traces of the observed types of arrhythmias are depicted in Fig. 7a, for each arrhythmia type, the duration was quantified per day and given as % of the recorded time (Fig. 7b–d). During the first two weeks after MI, short periods with non-sustained accelerated idioventricular rhythm (AIVR) and non-sustained ventricular tachycardia (VT) were seen in a few animals. No relevant burden of sustained AIVR or sustained VT was observed during this time (Fig. 7b, d). Despite the presence of non-contractile scar tissue in untreated NHP hearts, we did not observe a relevant burden of arrhythmia for the PBS-treated control animals #09, #11, #14, and #15 (Fig. 7d).

A distinct increase of non-sustained and sustained AIVR was apparent during the follow-up of animals treated with hiCMA injection from “*Amber*” (#05, #06, #07; Fig. 7b, left side). However, latency of this arrhythmic period after cell application varied between animals. Whereas animals #06 and #07 showed an increase of arrhythmia duration as early as 9 and 11 days, respectively, after hiCMA injection, animal #05 showed no significant arrhythmias within the first 18 days after treatment. However, animal #05 was the only example showing a relevant burden of sustained VTs three weeks after cell injection (Fig. 7b). In all “*Amber*” hiCMA-treated animals the duration of ventricular arrhythmias (AIVR + VT) peaked at 4–6 weeks after cell transplantation, followed by a steady decrease (Fig. 7c). In contrast, no relevant arrhythmia burden was observed for any of the “*Ruby*” hiCMA-treated animals (Fig. 7b, right side). No relevant bradyarrhythmias (including asystoles) were detected in any of the animals assessed in the study (data not shown).

In light of the differences in the in vivo arrhythmogenicity observed for the two cell lines, we have further applied patch clamp analysis for investigating the in vitro electrophysiological properties of “*Amber*”- vs “*Ruby*”*-*derived CMs as well as the differential expression of cardiac conduction-associated genes in respective BCTs (Fig. S8). The notably reduced action potential (AP) amplitude, upstroke velocity, and maximum sodium current density in “*Ruby*” CMs (Fig. S8A–D) is in line with the clone-specific downregulation of dominant voltage-gated sodium channels encoded by SCN5A and SCN4B as well as the differential expression of other genes involved in cardiac conduction (Fig. S8e). These include depolarization-associated genes encoded by HCN4 and SLC8A1, which are known for their role in the development of engraftment arrhythmias^26^. However, for the depolarization-associated gene CACNA1H and the hyperpolarization-associated gene KCNJ2, both of which influence CM automaticity, no differential expression levels between “*Amber*” and “*Ruby*”-derived BCTs were observed (Fig. S8e). The electrophysiological differences, including lower AP amplitude and upstroke velocity, are reflected in decreased overall force generated by the “*Ruby*” BCTs (Fig. S8f). The somewhat increased spontaneous activity observed in “*Ruby*” CMs (Fig. S1h) seems to corresponds to the higher spontaneous beating frequency in the “*Ruby*” BCTs (Fig. S8g).

## Discussion

In this study, we have addressed a number of challenges towards the clinical translation of hiPSC-CMs for heart repair, including (i) advanced cell production and maintenance protocols, (ii) suitable pre-clinical animal models and ways to deal with their limitations, and (iii) strategies for the more efficient and safer application of cell therapies to the heart.

Building on our established suspension culture technologies^2,4^, clinically relevant numbers of hiPSC-CMs were generated via chemically defined and, in principle, GMP-compliant conditions. It is noteworthy that the generated CM aggregates, across numerous independent production batches, were highly homogeneous regarding the physical properties and the high CM content of ~95–98% remaining phenotypical stable for up to 5 weeks of prolonged culture maintenance. This enables the spatial and temporal uncoupling of hiCMA production from their transplantation site in a cryopreservation-independent manner, which is of key importance for cell therapy logistics. The low batch-to-batch variation in CM content revealed by flow cytometry for cTnT, SA, MLC2a, and MLC2v emphasizes the high reproducibility and homogeneity of our production approach; a mixed composition of CMs pre-dominantly expressing one or the other myosin light chain (MLC2a or MLC2v) isoform is notably neither indicative of a batch-to-batch heterogeneity nor of a ventricular- vs. atrial-like phenotype but rather suggests a transient maturation state^27^.

Previous patch clamp analyses of our suspension-derived CMs revealed a predominantly ventricular-like immature phenotype, while not excluding a minor proportion of atrial-like cells^2,28,29^, which has been confirmed for both cell lines “*Amber*” and “*Ruby*” tested in this study. However, AP patterns of “*Ruby*”-CMs indicate lower sodium currents, which seems to correlate with their reduced expression of the two subunits SCN5A and SCN4B of Nav1.5 (Fig. 6c), the predominant sodium channel in the ventricular mouse and human myocardium^30^.

Despite the highly similar differentiation efficiencies of individual CM batches derived from the two different reporter iPSC lines, significantly different cell line-dependent contraction force levels were observed in our BCT assay. While Mannhardt et al. reported a substantial variability in active forces of iPSC-CM-derived engineered tissue among unrelated iPSC lines^31^, the phenotypical differences observed here seemed surprising as both cell lines were derived from the same parental line “*Phoenix*” and were genetically modified with the same experimental procedures. Underlying reasons may include clonal genetic or epigenetic aberrations introduced in the wake of transgene integration and the downstream single cell cloning, and/ or may result from the different transgenes i.e., nuclear Venus (a GFP variant) expressed in “*Amber*” or the co-introduced nuclear RedStar and GCaMP6f reporters in “*Ruby*”; our combined analyses indeed strongly suggest a major impact of the reporter transgenes.

Studies have suggested that calcium sensors such as GCaMP could lead to reduced contraction forces by acting as a calcium buffer, but Saleem et al. did not observe this phenomenon in engineered heart tissue^32^. In our case, force measurements on BCTs derived from “*Amber*” and “*Ruby*”—when compared with BCTs from the maternal line “*Phoenix*” and two single-transgenic * “Phoenix”*-derivatives expressing either GCaMP6f or RedStar—suggest that the nuclear DsRed red fluorescent protein (RFP) variant RedStar^16^ leads to a dysregulated expression of certain cardiac genes and the impaired contractile function in our study.

While this has not been reported before for monomeric RFPs such as mCherry or dtTomato^33^, and also not for RedStar, the tetrameric DsRed was found to cause problems in certain applications^34^. Another study reports distinct changes in gene expression in the hearts of mice overexpressing RFP, resulting in fibrosis, impaired protein degradation and severe cardiac dysfunction^35^. Interestingly, we found some reminiscent gene expression patterns in the “*Ruby*” graft in animal #10 (Fig. S9), potentially hinting towards a similar mechanism of RFP aggregation-mediated cardiac toxicity in “*Ruby*”-CMs, -BCTs, and -cardiac grafts as well. While future investigations are mandatory to fully uncover the underlying mechanisms, our work reveals previously unexpected reporter transgene-induced modulations of hiPSC-CM properties in vitro, suggesting their impact on cardiac recovery and arrhythmogenicity in vivo, which has substantial consequences for future studies in the field.

Our model of sub-acute MI was developed to explore the potential of hiCMA injection for heart function recovery in cynomolgus monkeys. These animals were found to infarct slowly compared to pigs but more similar to humans; at the same time their collateral myocardial blood flow is low and does not significantly influence the infarct size^36^ and functional recovery. Similar to Shiba et al.^12^, Chong et al.^9^, Liu et al.^11^, and Kobayashi et al.^10^, we have decided to inject hiCMAs at sub-acute conditions that is only two weeks after experimental MI induction. This reduces the risk of grafted cell depletion due to acute inflammation directly post-MI and promotes cell engraftment prior to scar formation. However, from the perspective of a cell therapy development, the relatively short time-frame of 2 weeks for cell delivery post MI is certainly not compatible with the patient-specific/autologous production of iPSC-CM, including the differentiation procedure and required quality controls etc. even if a stock of cryopreserved patient-specific iPSCs would be available. Therefore, the therapeutic concept of cellular therapy after subacute MI is rather compatible with an allogeneic transplantation strategy. This may potentially apply to HLA-matched-, HLA-homozygote-, or other genetically engineered- cells, compliant with decreased immunogenicity. However, transplantation of these cells would likely still require some degree of immunosuppression. In order to explore the concept of an autologous cell therapy, requesting at least 6 months of upstream time for donor CM production and characterization, a chronic-like MI model needs to be developed in future studies.

Our data confirm that proper immunosuppression enables the long-term survival and structural integration of human CMs in macaque hearts for at least 3 months, as also demonstrated by others^9,11^. We thereby demonstrate that human CMs can be injected in the form of distinct cell aggregates, which are remodeling in vivo to form larger human grafts as revealed by our histological results. We also noted the presence of host-derived endothelial cells in the human grafts already 2 weeks after CM injection, suggesting fast vascularization of the aggregate-based grafts, yet another support of our transplantation strategy. It has to be noted though that the largest and most mature grafts were observed in areas of healthy donor myocardium; cardiac T1 mapping and assessment of ECV by MRI also demonstrated the presence of residual scar tissue even after treatment in accordance with published work^37,38^. For a more complete remuscularization of the injured myocardium, a targeted application to the infarct border zones and the scar area might be an adequate option, which needs to be explored.

Published data based on low numbers of treated animals suggested the substantial improvement of heart function after transplantation of ~10^9^ single cell hESC-CMs per NHP heart^9,11,12^. While these studies demonstrated the formation of substantial hPSC-derived donor grafts, relatively large numbers of single-cell CMs had to be applied to achieve these results. Based on previous studies by us and others suggesting the substantial cell loss immediately after cell injection by both backflush from the injection channel and in particular the venous route^39,40^, we hypothesized that the application of cell aggregates, rather than single cells, may support the cell retention and engraftment efficiency after intra-myocardial injection. Indeed, our application of hiCMAs of ~200 µm average diameter led to the formation of relatively large grafts, despite using relatively low cell numbers of 5 × 10^7^ CMs/NHP heart. This confirms data from a recent study by Kobayashi et al., which applied cardiac spheroids of a similar size compared to our approach, but generated via an additional culture step starting from dissociated and cryopreserved iPSC-CMs prior to transplantation^10^. However, due to the limited number of animals that can be tested due to ethical, regulatory and financial restrictions, our as well as the Kobayashi study have not performed a direct comparison of the aggregate approach versus single cell administration in NHPs.

One striking result is the significantly lower CM density of 90,000 ± 7500 cells/mm^3^ found in our hiCMA-based grafts compared to values of ~242,000 cells/mm^3^ reported by Liu et al. for the injection of single cell suspension CMs^11^. In the human heart, due to the progressive, maturation-related CM hypertrophy, the CM density was found to decrease from 430.000 ± 72,000 cells/mm^3^ shortly after birth down to 28,000 ± 7200 cells/mm^3^ in subjects aged 20–73^41^. Thus, our aggregate approach, including our extended cultivation step, might promote the generation of more mature myocardium due to transplantation of well-connected CMs in a proper microenvironment. Given our promising results, a head-to-head comparison of these alternative approaches deems relevant, eventually considering alternative experimental setups compatible with higher numbers of experimental repeats.

The small numbers of animals typical for NHP studies lead to additional challenges for demonstrating statistically robust data on the functional heart recovery after MI and cell administration vs controls. Already for the pre-MI LVEF values a substantial degree of variation was observed. This is partially due to the inter-individual differences in animals’ age, weight and coronary artery anatomy; however, the outlying low values for animals #05 and #10 (Table 1) can be explained by technical reasons. These two animals were evaluated using the Real-Time approach due to distorted ECG signals (preventing ECG-gated segmented cine Fast Low-Angle Shot (FLASH) technique), while standard CINE MRI was used for assessing all other animals. To account for this limitation, we decided to determine heart function as LVEF relative to the pre-MI LVEF; this normalization allowed for the comparative analyses in small groups of large animals. After MI, the extent of LVEF reduction varied based on animals’ anatomy and positioning of the coronary artery ligation, but was successful in most animals with a loss of contractile myocardium in left ventricular and septal areas near the apex. While it is known that there are non-cardiac causes of troponin elevation in humans^42^, we found that after experimental MI in our model, serum levels of cTnT can be used as valuable biomarker not only for the presence of myocardial damage, as described by ref. ^43^, but also for the extent of scar formation and LVEF reduction. Thus, early cTnT measurements could serve as inclusion-exclusion criteria in future NHP experiments. Compared to cTnT, total CK, and CK-MB were found to be less reliable markers as expected, due to the reduced cardiac specificity of CK-MB in cynomolgus monkeys compared to humans^43^.

The cell-treated group B showed substantial heart function recovery already after 2 weeks and particularly after 3 months, but only for animals receiving “*Amber*”-hiCMAs whereas no LVEF increase resulted from “*Ruby*”-based treatments. The extent of recovery after 3 months of “*Amber*” hiCMAs injection was very similar to that observed by Liu et al. after the application of about 20-fold (!) larger numbers of dissociated, cryopreserved CMs^11^ and to the degree of recovery described by Kobayashi et al. for a similar number of re-aggregated CMs^10^. Although we could only exemplarily quantify human graft dimensions, i.e., in animals #07 (receiving “*Amber*” hiCMAs), #12 and #13 (receiving “*Ruby*”-hiCMAs), our data show the formation of substantial grafts from both reporter cell lines. This argues against the possibility of poor graft formation underlying the lack of heart function recovery in *‘Ruby’* treated animals. Moreover, no apparent hiPSC clone-related differences in human graft structure and marker proteins expression was observed.

However, RNASeq analyses indicated that there are substantial differences in the expression of genes related to cardiac muscle contraction and muscle filament sliding in “*Amber*” vs “*Ruby*”-hiCMAs, BCTs and grafts as well; in general, gene expression patterns in “*Amber*” derivatives were more similar to native human myocardium compared to “*Ruby*”. Together with our in vitro BCT “potency/contraction assay” data, it is tempting to speculate that “*Amber*”-hiCMAs promote heart function recovery mainly by their contractile properties, although we cannot exclude additional paracrine or other indirect mechanisms discussed in the field^44–46^. Clearly, the contribution of contractile forces of the engrafted CMs to heart function recovery needs more direct assessment of electromechanical donor-host coupling in the future. However, our observations and reports on the substantial variability in active forces of iPSC line-specific engineered heart tissues^31^ highlights both the requirement for a suitable in vitro potency assay (also requested by regulatory authorities)^47,48^ and the utility of our study as a practical solution for such an assay.

We here confirm the occurrence of graft-induced ventricular arrhythmias as described in the literature^9,11^. The origin of hPSC-CMs application-related arrhythmias is vividly discussed in the field, including the potential presence of pacemaker cells and the immaturity of the transplanted CMs^49^. In our study, the observed arrhythmias were transient and non-life-threatening but may call for a temporary anti-arrhythmic treatment in future patients. We have observed a delayed onset of arrhythmias starting not before two weeks after the injection of “*Amber*”-hiCMAs; this may result from the administration of the anti-arrhythmic drug amiodarone^50^ applied for up to 9 days after cell injection.

In parallel to the lack of functional heart recovery, we did not observe arrhythmias after the injection of “*Ruby*”-hiCMAs despite the observed formation of large grafts. A recent study by Marchiano et al.^26^ suggests that the immature phenotype of ventricular-like hPSC-CMs underlies graft-related arrhythmia. Interestingly, we found that the ion channel encoding genes HCN4, and SLC8A1, which have been knocked out in combination in hESC-CMs by Marchiano et al. to avoid respective arrhythmias, are downregulated in “*Ruby*”- compared to “*Amber*”- CM- derived BCTs (in addition to the sodium channel encoding genes SCN5A and SCN4B), while the hyperpolarization-associated gene KCNJ2 (overexpressed in the Marchiano study) and the channel CACNA1H (also knocked-out by Marchiano et al. combined with HCN4 and SLC8A1) show variable expression between our “*Amber*”- versus “*Ruby*”-BCT samples. Since gap junctions and thus connexins likely play a decisive role in the induction of arrhythmia by the grafted CMs, we have also analyzed gap junction proteins in “*Amber*”- and “*Ruby*”-BCTs and observed upregulation of Connexin 43 (Cx43; encoded by GJA1) in “*Ruby*”-BCTs. These combined data may suggest that transplanted “*Ruby*”-hiCMAs may actually couple to the host myocardium (given the upregulation of Cx43) but apparently do not induce arrhythmias potentially due to their low expression of the depolarization-associated genes HCN4, SLC8A1, and CACNA1H as well (Fig. S8E), in agreement with the findings by ref. ^26^. In contrast, “*Amber*”-hiCMAs do apparently transmit their autonomous depolarization activity (associated with their developmental immaturity) to the host myocardium despite their reduced Cx43 expression (relative to “*Ruby*”-CMs), which, however, may contribute to the relative late onset of arrhythmogenicity observed in our model.

Besides therapies’ efficiency, patients’ safety will be of utmost importance for the clinical translation. We are tempted to speculate that the injection of hiCMAs and their efficient engraftment observed in this study has the potential to reduce treatment-related risks. The application of relative low cell doses and the hypothesized lower leakage of cells into the circulation lowers the risks of undesired downstream accumulation, inflammation and engraftment in organs such as the lung or liver, as compared to previous single cell transplantation approaches^40^.

However, following promising results in guinea pigs, pigs^51^ and more recently in macaques, a first clinical trial for heart repair via engineered cardiac patches from hiPSC-CMs was initiated in Germany^52^. This surgical approach of tissue (rather than cell) delivery overcomes some challenges related to the retention of injected cells, but is associated with others. Those include, for example, the questionable functional coupling of tissue grafts placed on the epicardium, which may remain isolated from the myocardium; it also remains to be seen how the strategy will perform regarding the primary endpoint of ATMP testing, that is patients’ safety in the short and long run.

Taken together, we show that the transplantation of hiCMAs resulted into the formation of substantial, structurally integrated muscular grafts and improved heart function in a NHP model of sub-acute MI. The application of CM aggregates, rather that single cells, enabled the usage of relatively small cell numbers, thereby promoting the efficiency of the procedure by reducing cell production costs and clinical risks associated with administering high numbers of cells and the accompanied cell death. According to our study, hiCMA injection can be considered reasonably safe and potential temporary arrhythmias should be manageable in the clinic. Since we have also demonstrated the temporal uncoupling of human CM production from transplantation and the implementation of an in vitro potency test, our study provides pragmatic new avenues for the clinical translation of hiPSC-based heart repair.

## Methods

We confirm that our research complies with all relevant ethical regulations. Collection of human blood and tissue samples was approved in accordance with institutional guidelines by the local research ethics committee (MHH no. 1303-2012). Animal experiments were approved by the Lower Saxony State Office for Consumer Protection and Food Safety.

### Differentiation and analysis of cardiomyocyte aggregates for transplantation and in vitro potency test

Human iPSC cultivation, chemically defined cardiac differentiation, and cell/aggregate analysis was performed essentially as described in refs. ^2,4^. In brief, the hiPSCs reporter line MHHi0001-A-11 (“*Amber*”, constitutively expressing Venus_nucmem_^15^ under control of the CAG promoter from the AAVS1 locus) or the line MHHi0001-A-5 (“*Ruby*”, constitutively expressing RedStar_nucmem_ and the calcium sensor GCaMP6f under control of the CAG promoter from the AAVS1 locus^14^) were expanded and used for single cell inoculation in suspension culture into 500 mL Disposable Spinner Flask with Vent Cap (Corning), stirred at 60 RPM. Directed cardiac differentiation was induced in the same Spinner platform: after check point 1 (CP1; see Fig. 1), resulting CM aggregates (hiCMAs) were cultured in RB^+^ medium (RPMI-B27 with insulin, Gibco; plus Penicillin/Streptomycin, Sigma-Aldrich) until check point 2 (CP2; see Fig. 1). The hiCMA diameter was assessed by the automatic analysis of microscopic pictures by ImageJ and the CM content of CP1/CP2 hiCMA samples was analyzed by flow cytometry. At CP2, hiCMAs were collected from the culture medium and used for cell transplantation (described below) or for the generation of BCTs^2,17^ to assess CM contractility in vitro. For details, see supplementary methods.

### Animal care

Cynomolgus monkeys (Macaca fascicularis; 1 female, 6.8 kg; 14 male 7.3–12.8 kg) were kept at the German Primate Center under conditions according to §§7–9 of the German Animal Welfare Act, adhering to the European Union guidelines (EU directive 2010/63/EU) on the use of NHP for biomedical research. The experiments were approved by the Lower Saxony State Office for Consumer Protection and Food Safety (33.12-42502-04-14/1404 and 33.12-42502-04-21/3694).

To prevent early infarction-related arrhythmias, animals were treated with amiodarone (100 mg day^−1^, oral) for 14 days starting 5 days before surgical induction of the MI. Metoprolol was administered twice daily (50 mg) for 11 days starting 2 days before surgery.

Immunosuppression started 5 days before cell transplantation with daily injections of cyclosporine A (10 mg kg^−1^ i.m. for 15 days, followed by 7.5 mg kg^−1^ to maintain serum through levels of 600 µg/L) and methylprednisolone (2.5 mg kg^−1^ i.m.) until animals were euthanized. In addition, belatacept (CTLA4-Ig, 10 mg kg^−1^ i.v.), an efficient T-cell costimulatory signal blocker suitable for chronic treatment in cynomolgus monkeys^53^ was administered on the day of cell transplantation, as well as on day 18, 28, 42, and end of week 10 after cell transplantation.

Marbofloxazin (4 mg kg^−1^ i.m. during surgery) and amoxicillin (0.05 mg kg^−1^; s.c.) were administered to prevent opportunistic infections. Post-surgery, wounds were carefully examined and animals were regularly monitored by laboratory (for hematocrit, hemoglobin levels) and Primate Center staff (for signs of distress indicating post-procedure pain).

At the end of the experiments, all animals were euthanized under deep anesthesia with pentobarbital after administration of 500IE/kg heparin i.v. Hearts were processed for histology and immunofluorescence staining as snap-frozen or paraffin-embedded samples. Staining and image analyses were performed as described in the supplementary methods; primary and secondary antibodies used are listed in Tables S1–S3.

### Animal model of myocardial infarction and cell transplantation

Animals underwent left thoracotomy and permanent ligation of the left anterior descending artery (LAD) below the first diagonal branch for the induction of left ventricular MI under general anesthesia. MI was confirmed by identifying a pale ischemic area and by subsequent serum assays for cardiac troponin and creatine kinase 1 day after surgery. Transesophageal cardiac echocardiography was performed before and after induction of infarction.

Two weeks following infarction, a re-thoracotomy was performed under general anesthesia. After opening the pericardium, the heart was exposed, and hiCMAs in phosphate-buffered saline (PBS) were injected into the peri-infarct region of the left ventricular wall via 10–12 injections each of 50 µl volume with a 26G needle. Each hiCMA sample injected per heart contained ~50 million CMs (calculated based on the analysis of parallel, representative hiCMA aliquots dissociated into single cells for counting). An equal volume of phosphate-buffered saline (PBS) was injected in control animals. For details, see supplementary methods.

### ECG monitoring

Continuous monitoring for clinically relevant arrhythmias was established using an implantable telemetry device (PhysioTel Digital M01, Data Sciences International (DSI), St. Paul, MN, USA). The device was implanted at the time of LAD ligation surgery during general anesthesia. DSI Ponemah® version 6.20 with Pattern Recognition Option (DSI, St. Paul, MN, USA) was used for ECG telemetry data collection and analysis. For details, see supplementary methods.

### Cardiac MRI

The monkeys underwent MRI measurements before MI (pre-MI), before cell transplantation (baseline), and 2 and 12 weeks after cell transplantation (Fig. 4a). All experiments were performed on a 3T MR-system (Magnetom Prisma, Siemens Healthineers, Erlangen, Germany) using a 16-channel multipurpose coil (Variety, Noras MRI products, Hoechberg, Germany) for signal reception in anesthetized and intubated monkeys.

Left ventricular function was assessed using an ECG-gated segmented cine fast low-angle shot (FLASH) technique as previously described in ref. ^54^, with manual segmentation using the software package Segment (Version 2.0 R6435, Medviso, Lund, Sweden)^55^. Real-Time MRI during free breathing was acquired using spoiled radial FLASH. Maps of the T1-relaxation time were acquired before and after contrast agent administration (Gadobutrol (Gadovist®), 0.1 mmol/kg per BW) using a novel fast Real-Time single-shot inversion recovery FLASH technique (imaging parameters)^56^. For details, see supplementary methods.

### Laser microdissection and RNASeq analysis

To facilitate compartment-specific analysis, graft target structures were isolated using the CellCut laser-microdissection System (MMI Molecular Machines & Industries AG, Eching, Germany). RNA was then isolated using the RNeasy FFPE Kit (Qiagen, Venlo, The Netherlands)^57^. RNA sequencing libraries were prepared using strand-specific protocol with ribosomal RNA depletion and sequenced on Illumina NovaSeq platform (PE 2 × 150 bp).

A comparison of gene expression between the defined groups of samples was performed via DESeq2. The Wald test was used to generate p-values and log2 fold changes. Genes with an adjusted *p*-value < 0.05 and absolute log2 fold change > 1 were called as differentially expressed genes for each comparison.

For Gene Ontology (GO) analysis, genes that demonstrated statistically significant differential expression were analyzed with GeneSCF v.1.1-p2, clustering genes according to biological processes with goa_human as the GO list.

Gene expression patterns for combined GO terms GO:0060048 (cardiac muscle contraction) and GO:0030049 (muscle filament sliding) were visualized as heatmap. For details, see supplementary methods.

Human atrial and ventricular heart samples were obtained after cardiac surgery in accordance with institutional guidelines after approval by the local research ethics committee (MHH no. 2997-2016).

### Statistical analysis

The GraphPad Prism Version 9 (GraphPad Software, Inc., La Jolla, CA, USA) was used for statistical analyses. Descriptive statistical analysis was expressed as Mean ± Standard Deviation. Continuous numerical variables were compared using the student’s *t*-test for independent samples, significance was evaluated by two-tailed testing, and assumed at *p* < 0.05.

To compare more than two groups, a two-way ANOVA with Tukey’s or Bonferroni’s multiple comparisons test was performed. A *p*-value < 0.05 was considered statistically significant.

### Reporting summary

Further information on research design is available in the Nature Portfolio Reporting Summary linked to this article.

## Supplementary information

Supplementary Information

Description of Additional Supplementary Files

Supplementary Video 1

Supplementary Video 2

Reporting Summary

Transparent Peer Review file

## Source data

Source Data

## Data Availability

The authors declare that all data supporting the findings of this study are available within the article and its Supplementary Information files, or in the source data provided with this paper. RNASeq data have been deposited in the NCBI Sequence Read Archive (SRA) under the BioProject ID PRJNA1248569. (https://www.ncbi.nlm.nih.gov/bioproject/?term=PRJNA1248569).
