# Supplementary material for: Human iPSC-cardiomyocyte-aggregate cell therapy in non-human primates and correlation of heart recovery with contractile and electrophysiological cardiomyocyte properties
Source: Nat Commun. 2026 Aug 26;17:9066. doi: 10.1038/s41467-026-77103-0 (PMC13518835; doi:10.1038/s41467-026-77103-0)
Supplement: Supplementary file 1 [file 41467_2026_77103_MOESM1_ESM.pdf]

Supplementary Information for

Human iPSC-cardiomyocyte-aggregate cell therapy in non-human primates and correlation of heart recovery with contractile and electrophysiological cardiomyocyte properties

Ina Gruh[1,2,3*], Andreas Martens[1,2,3*], Serghei Cebotari[1,2,4], Annette Schrod[5], Alexandra Haase[1,2], Caroline Halloin[1,2], Wiebke Triebert[1,2], Nils Kriedemann[1,2], Tobias Goecke[1,2,6], Morsi Arar[2,3], Klaus Hoeffler[2], Paul Frank[7], Karen Lampe[5], Amir Moussavi[5], Anna Kuleshova[1,2,], Vojtech Hradil[1,2], Veronika Fricke[1,2], Monika Szepes[1,2], Kerstin Mätz-Rensing[5], Jörg Eiringhaus[7], Anna-Lena de Vries[7], Stephan Hohmann[7], Merlin Witte[1,2], Tim Kohrn[1,2], Jana Teske[1,2], Kevin Cyrys[1,2,], Sara Szadocka[1,2], Annika Franke[1,2], Christopher Werlein[8], Karlo Komorowski[9], Mark Kühnel[10], Danny Jonigk[10], Susann Boretius[5], Christian Veltmann[7], David Duncker[7], Jonathan Jung[11], Nissim Benvenisty[11], Jeanne de la Roche[12], Martin Fischer[12], Axel Haverich[1,2], Arjang Ruhparwar[2,6], Robert Zweigerdt[1,2], Ulrich Martin[1,2,6]

[1]Leibniz Research Laboratories for Biotechnology and Artificial Organs, Cluster of Excellence REBIRTH, Carl-Neuberg-Straße 1, Hannover Medical School, 30625 Hannover, Germany
[2]Dept. of Cardiac, Thoracic, Transplantation and Vascular Surgery, Cluster of Excellence REBIRTH, Hannover Medical School, Carl-Neuberg-Straße 1, 30625 Hannover, Germany
[3]Dept. of Cardiac Surgery, Carl von Ossietzky University Oldenburg, Carl-von-Ossietzky-Straße 9-11, 26129 Oldenburg, Germany
[4]Institute for Cardiac Surgery and Interventional Cardiology, INCCI Heart-Center Foundation, Dept. of Cardiac Surgery, 24, rue Nicolas Ernest Barblé, 1210 Luxembourg, Luxembourg
[5]German Primate Center, Leibniz Institute for Primate Research, Kellnerweg 4, 37077 Göttingen, Germany
[6]Biomedical Research in Endstage and Obstructive Lung Disease (BREATH), German Center for Lung Research (DZL), Hannover Medical School, Hannover, Germany
[7]Hannover Heart Rhythm Center, Department of Cardiology and Angiology, Hannover Medical School, Carl-Neuberg-Straße 1, 30625 Hannover, Germany
[8]Department of Pathology, Hannover Medical School, Carl-Neuberg-Straße 1, 30625 Hannover, Germany
[9]Department of Radiation Protection and Medical Physics, Hannover Medical School, Hannover, Germany
[10]Institute of Pathology, University Hospital RWTH Aachen, Pauwelsstraße 30, 52074 Aachen, Germany
[11]Department of Genetics, The Hebrew University of Jerusalem, Givat Ram Jerusalem 9190401, Israel
[12]Institute of Neurophysiology, Hannover Medical School, Carl-Neuberg-Straße 1, 30625 Hannover, Germany

** these authors contributed equally*

Supplementary Methods

hiPSC culture, differentiation and analysis of cardiomyocyte aggregates

Human iPSC cultivation, chemically defined cardiac differentiation, and cell/ aggregate analysis was performed essentially as described [1, 2]. Monolayer cultures of the hiPSCs reporter lines MHHi0001-A-11 ('*Amber*', constitutively expressing $\text{Venus}_{\text{nucmem}}$ [3] under control of the CAG promoter from the AAVS1 locus) or MHHi0001-A-5 ('*Ruby*', constitutively expressing $\text{RedStar}_{\text{nucmem}}$ and the Calcium sensor GCaMP6f under control of the CAG promoter from the AAVS1 locus [4], both derived from the *'Phoenix'* line MHHi001-A [5] were expanded in E8 medium on Geltrex-coated T75 and T175 flasks. For force measurement in BCTs, also one *'Phoenix'*-derived line constitutively expressing $\text{RedStar}_{\text{nucmem}}$ and another Phoenix-derived line MHHi001-A-8 constitutively expressing GCaMP6f [6], in both cases under control of the CAG promoter from the AAVS1 locus [4] were used.

For single cell inoculation in suspension culture, cells were dissociated by 3-5 min of Accutase (Thermo Fisher Scientific) treatment and seeded at 0.15 x 10^6cells/ml in 300 ml of E8 medium supplemented with 10 mM Y-27632 (Rock-Inhibitor, RI; [7] and 0.1% Pluronic-F68 solution (Gibco, PLU) into 500 mL Disposable Spinner Flask with Vent Cap (Corning), stirred at 60 RPM for 48h.

Differentiation of the resulting hiPSC aggregates was initiated in CDM3 medium supplemented with 5 µM CHIR99021 (Tocris Bioscience) in the same Spinner platform. Precisely 24h later, the medium was replaced by fresh CDM3 supplemented with 5 µM IWP2 (Tocris Bioscience). Precisely 48h later, CDM3-IWP2 was replaced with pure CDM3 and 75% medium was replaced every 2–3 days thereafter. After check point 1 (CP1; see Fig. 1) of differentiation, resulting cardiomyocyte aggregates (hiCMAs) were cultured in RB^+ medium (RPMI-B27 with insulin, Gibco with Penicillin/Streptomycin, Sigma-Aldrich) replaced every 3-4 days until check point 2 (CP2; see Fig. 1).

To monitor the hiCMA diameters, up to twelve independent light microscopic images of hiCMA samples (placed in 6-well plate) were captured at 4x magnification (Olympus CKx41 inverted microscope, Olympus) followed by automated diameter analysis via ImageJ

(https://imagej.nih.gov/ij/); mean diameters represent the arithmetic average of at least 70 individual aggregates.

Patch clamp technique

hiPSC-derived cardiomyocytes originating from *'Amber'* or *'Ruby'* hiPSC line were dissociated into single cells and seeded on Geltrex-coated glass coverslips in a 12-well plate with a density of 20,000 cells per well. 2-4 days after seeding electrophysiological properties of cardiomyocytes were investigated by whole-cell patch clamp recordings obtained at room temperature in current-clamp mode for action potential registration and in voltage-clamp mode for acquisition of underlying ionic currents. The extracellular Tyrode solution contained 140 mM NaCl, 5.4 mM KCl, 1.8 mM $CaCl_2$, 1 mM $MgCl_2$, 10 mM HEPES, 10 mM glucose, adjusted with NaOH to pH 7.4. The intracellular solution contained 120 mM K-gluconate, 10 mM Na-gluconate, 1 mM $MgCl_2$, 10 mM HEPES, 10 mM EGTA/KOH, 3 mM Mg-ATP, with pH adjusted to 7.2 by KOH. The liquid junction potential (+16.2 mV) was calculated using the JPCalc software (Dr. P. Barry, University of South Wales, Sydney, Australia) and corrected *a priori*. Pipettes were pulled from borosilicate glass capillaries (1.2 mm O.D. × 0.94 mm I.D. Harvard Apparatus, GC120TF-10) using a Sutter puller (Model P-97, Sutter Instruments). Polishing by microforge (MF-900, Narishige) provided pipette tips with resistances of 1.7 – 5.0 MΩ. Whole-cell recordings in current- and voltage-clamp mode were performed using an Axopatch 200B amplifier and Digidata 1550 (Molecular Devices). Data was sampled with a rate of at least 10 kHz and low-pass filtered at 5 kHz. Sodium currents were examined as response to voltage steps from a pre-potential of -120 mV to potentials between -80 mV and +80 mV. With a pre-potential of -40 mV, sodium channels inactivated and allowed recording of calcium currents. Steady state potassium currents were recorded after holding the membrane voltage at positive potentials for 3 seconds. HCN currents were determined as slowly increasing inward currents in response to hyperpolarizing voltage steps. pCLAMP software (Molecular Devices), Prism 7 (Graph Pad Software) and CorelDRAW (CorelDRAW Graphics Suite) were used for data analysis and presentation.

Flow cytometry

For CM content monitoring on CP1 / CP2, hiCMA samples were dissociated by STEMdiff Cardiomyocyte Dissociation Kit (Stem Cell Technologies) following supplier's instructions. For flow cytometry analysis, cells were treated with FIX&PERM Solution (Thermo Fisher Scientific) and stained with respective antibodies specific to anti-cardiac Troponin T (cTnT; 1:200, clone 13-11, Thermo Fisher Scientific), anti-sarcomeric α-Actinin (1:800, EA53, Sigma-Aldrich or 1:20, REA402, Miltenyi Biotech), anti-Myosin Heavy Chain (1:20, MF20, DSHB), or isotype control mouse-IgG1 (1:200, DAKO). The primary antibodies were detected by appropriate Cy5-conjugated antibodies (1:200, Jackson ImmunoResearch) followed by data acquisition on a MACSquant Analyzer 10 Flow Cytometer (Miltenyi Biotec) and data analysis by FlowJo software (Flowjo, LLC).

Generation of bioartificial cardiac tissue and force measurement

For the generation of BCTs, the hiCMAs were dissociated and 1 million CMs were used for tissue preparation as described [8]. CMs were mixed with 10% human dermal fibroblasts (ATCC) in 81 µl BCT medium per tissue. An extracellular matrix mixture (i. e. 178 µl/ BCT) composed of 2 mg/ml rat collagen type I (R&D Systems), 14% Geltrex (Life Technologies), and 5.3% 0.4 M NaOH was added. The cell-matrix mixture was poured into a custom-made silicon mold containing two titanium rods (distance 6 mm; initial slack length) and solidified at 37 °C for 30 minutes. Then, the construct was covered by 5 ml BCT medium with 60 µM L-ascorbic acid (Sigma-Aldrich) and cultured under standard cell culture conditions with medium change every 1 or 2 days. For all BCTs, growing static stretch (G-stretch) was applied through stepwise elongation by 400 µm on days 7 and 11. Tissue morphology was monitored during cultivation using an AxioObserver Z1 fluorescence microscope or the Stereo Microscope Discovery V8 (Zeiss, Jena, DE). Diameters of the tissues on day 14 of cultivation were measured using the ZEN or AxioVision SE64 Rel. 4.9 software respectively, and the cross-sectional area was calculated presuming a circular geometry.

Mechanical forces of the tissues were measured on day 14 of tissue cultivation (equivalent to CP3 *in vivo*) in BCT medium at 37 °C, 5% CO_2 levels using a custom-made bioreactor system and analysis software (Central Research Workshop, Hannover Medical School) as previously described [8]. Active contraction force was determined at increasing preload (in 200 µm increments until 1.2 mm in total) by measuring the response to electric stimulation (5x) with biphasic pulses (10 msec, ±25 V). Force-frequency relationship was determined at maximum preload by biphasic stimulation of tissues between 1 and 3 Hz for the duration of 20 sec. Passive force for each preload step was defined by the difference between baselines at the actual step and at the original length (L0).

For video-optical calcium analysis, videos at a sampling rate of 47 frames / sec for 15 sec were acquired using the AxioObserver Z1 fluorescence microscope (Zeiss, Jena, DE). Changes in fluorescence intensity while spontaneous contractions were calculated using the ZEN module Physiology (Dynamics) of the ZENpro software (Zeiss, Jena, DE). After selection of a Region of Interest (ROI) and defining an area for background correction, the ratio calculation for the fluorescence channel was performed as previously described by Tian, Martinez et al. [9].

Cell preparation for transplantation

For simulation of the injection procedure during transplantation, hiCMAs were collected at CP2 and passed through a 26G syringe. The viability score of the resulting aggregate solution was analyzed in comparison to an untreated sample as previously described [10]. Briefly, hiCMAs were stained with calcein-AM (Ca-AM) and ethidium homodimer-1 (EtHd-1) and incubated for 60 min at 37 °C and 5% CO2 in an incubator on a horizontal shaker at 70 rpm. The viability score was determined as a ratio of the Ca-AM and the EtHd-1 signal.

For transplantation, hiCMA were collected from the culture medium by 1 min centrifugation at 300g. After media removal, the hiCMA pellet was carefully washed with DPBS (Gibco), re-collected by centrifugation and carefully filled-up with DPBS to a total volume of 800 µl in scaled 1.5 ml tubes (StarLab). Each hiCMA sample injected per heart contained ~50 million CMs (calculated based on the analysis of parallel, representative hiCMA aliquots dissociated into

single cells for counting). For injection, the ~ 800 µl of hiCMAs/ DPBS suspension was carefully transferred to a 26G syringe.

Animal anesthesia

For all major surgery, animals were pre-medicated with glycopyrronium bromide, medetomidine, maropitant, and meloxicam, then anaesthetized with ketamine (10 to 15 mg kg^{-1}) and propofol, (0.4 to 0.6 mg kg^{-1} min^{-1}), intubated and ventilated using isoflurane to maintain anesthesia. Perioperative and postoperative analgesia was performed with fentanyl (0.1 to 0.5 µg kg^{-1} min^{-1} i.v.), buprenorphine (5-10 µg kg^{-1} s.c.) and bupivacaine hydrochloride (1 mg kg^{-1}, intercostal nerve blockade).

Echocardiography

Trans-esophageal cardiac echocardiography was performed before and after induction of infarction under general anesthesia. Images were captured with an EPIQ 7G device using an S7-3t trans-esophageal phased ultrasound transducer as four–chamber, two-chamber and short axis views. Functional analysis was performed using a CX50 device and software version OS-12.676 (all from Philips N.V. Amsterdam, Netherlands).

ECG monitoring

Continuous monitoring for clinically relevant arrhythmias was established using an implantable telemetry device (PhysioTel Digital M01, Data Sciences International (DSI), St. Paul, MN, USA). The device was implanted subcutaneously in the abdominal region at the time of LAD ligation surgery during general anesthesia. Leads were tunneled to achieve a lead II configuration and a single lead ECG was continuously recorded from day 0 (induction of myocardial infarction) until sacrification of the animal. ECG recordings were analyzed with focus on relevant bradyarrhythmias and tachyarrhythmias and automated results were reviewed by a cardiologist [11]. DSI Ponemah® version 6.20 with Pattern Recognition Option (DSI, St. Paul, MN, USA) was used for ECG telemetry data collection and analysis. Ventricular arrhythmias were classified as

premature ventricular complexes (PVCs; QRS duration >60 ms), ventricular couplets, ventricular triplets, non-sustained accelerated idioventricular rhythms (AIVR; heart rate <180 bpm), sustained AIVR, non-sustained ventricular tachycardia (VT; heart rate ≥180 bpm) or sustained VT as described by Liu et al. [12]. AIVR and VT were considered sustained if ≥30 conjugated respective wide complexes occurred. Representative ECG tracings are shown in Fig.7a. Arrhythmia burden was quantified as number of events per day for each arrhythmia type as well as for the total ventricular arrhythmia burden (aggregate of VT and AIVR).

Cardiac MRI

The monkeys underwent MRI measurements before myocardial infraction (pre-MI), before cell transplantation (baseline), and 2 and 12 weeks after cell transplantation (Fig. S4a). All experiments were performed on a 3T MR-system (Magnetom Prisma, Siemens Healthineers, Erlangen, Germany) using a 16-channel multipurpose coil (Variety, Noras MRI products, Hoechberg, Germany) for signal reception. The anesthetized and intubated monkeys were mechanically ventilated (Servo Ventilator 900C, Siemens-Elema AB, Sweden) and physiological parameters (e.g., heart rate, breathing rate, and end-tidal CO_2) were continuously monitored.

For the assessment of the left ventricular function, short-axis images were acquired during forced expiratory breath holds using an ECG-gated segmented cine Fast Low-Angle Shot (FLASH) technique (repetition time (TR) = 6.5 ms, echo time (TE) = 2.8 ms, flip angle = 12°, bandwidth = 215 Hz/Px, field of view (FOV) = 132 × 149 mm², 6 segments, spatial resolution = 0.85 × 0.85 × 3 mm^3, 13–17 slices covering the heart from the apex to the base with an acquisition time of 15-20 seconds per slice) as previously described [13]. In addition, Real-Time MRI during free breathing was acquired using spoiled radial FLASH (TR = 2.9 ms, TE = 1.9 ms, flip angle = 8°, bandwidth = 1305 Hz/Px, FOV = 128 × 128 mm², base resolution = 128, 13 spokes per frame, Nyquist undersampling factor = 15.5, spatial resolution = 0.9 × 0.9 × 3 mm^3, acquisition time per frame = 37 ms, 13-17 slices and 210 frames per slice). In case of a corrupted ECG signal (animal #05, and #10), the ventricular function was assessed using Real-Time MRI instead of Cine MRI at all four time points of these monkeys.

To assess the volume and function of the left ventricle, the myocardium was manually segmented in a blind fashion by two observers with at least two years of experience in cardiovascular MRI analyses of primates using the software package Segment (Version 2.0 R6435, Medviso, Lund, Sweden) [14]. Besides the standard volumetric and functional parameter of the left ventricle (end-diastolic volume (EDV), end-systolic volume (ESV), stroke volume (SV), ejection fraction (EF)) the relative EF (rEF) was defined as the EF normalized by the respective pre-MI EF of the same monkey. Noteworthy to mention, despite systematic differences in the estimated absolute values of EF acquired by cine and Real-Time MRI, no significant differences in rEF are expected [13].

Maps of the T1-relaxation time were acquired in the 4-chamber view before and 15 minutes after contrast agent administration (Gadobutrol (GadovistTM), 0.1 mmol/kg per BW) using a single-slice fast Real-Time single-shot inversion recovery FLASH technique (TR = 2.7 ms, TE 1.8 = ms, flip angle = 6°, bandwidth = 1345 Hz/Px, FOV = 128 × 128 mm^2, base resolution = 128, 15 spokes per frame, Nyquist undersampling factor = 13.4, spatial resolution = 1 × 1 × 4 mm^3, acquisition time per frame = 40 ms, total acquisition time = 3 s) [15]. T1-relaxation times and extracellular volumes (ECV) were estimated within the region of the scar after infarction as well as in an unaffected region of the myocardium (remote). The ECV was calculated as the hematocrit (hct) normalized ratio of pre- and post-contrast R1-relaxation constant (R1 = 1/T1) differences of the myocardium and blood, respectively (ECV = (1−hct) × (ΔR1myocardium/ΔR1blood)). A hematocrit of 0.4 was estimated for all animals.

Laser microdissection and RNASeq analysis

To facilitate compartment-specific analysis, 5 µm thick formalin-fixed, paraffin-embedded (FFPE) tissue sections were mounted on a membrane attached to a metal frame. Following deparaffinization and haemalum staining, the CellCut laser-microdissection System (MMI Molecular Machines & Industries AG, Eching, Germany) was used isolation of target structures. RNA was then isolated using the RNeasy FFPE Kit (Qiagen, Venlo, The Netherlands) [16].

RNA sequencing libraries were prepared using strand-specific protocol applying NEBNext Ultra II RNA Library Prep Kit (NEB, Ipswich, MA, USA) and sequenced on Illumina NovaSeq platform (PE 2 x 150 bp). Raw data quality was evaluated with FastQC. Sequence reads were trimmed to remove possible adapter sequences and nucleotides with poor quality using Trimmomatic v.0.36. Post-trimming, reads were mapped to the Homo sapiens GRCh38 reference genome from ENSEMBL using STAR aligner v.2.5.2b. Unique gene hit counts were calculated by using featureCounts from the Subread package v.1.5.2. The hit counts were summarized and reported using the gene_id feature in the annotation file. Only unique reads that fell within exon regions were counted.

A comparison of gene expression between the defined groups of samples was performed via DESeq2. The Wald test was used to generate p-values and log2 fold changes. Genes with an adjusted p-value < 0.05 and absolute log2 fold change > 1 were called as differentially expressed genes for each comparison.

For Gene Ontology (GO) analysis, genes that demonstrated statistically significant differential expression were analyzed with GeneSCF v.1.1-p2, clustering genes according to biological processes with goa_human as the GO list.

Gene expression patterns for combined GO terms GO:0060048 (cardiac muscle contraction) and GO:0030049 (muscle filament sliding) were visualized as heatmap. The heatmap2 tool on the Galaxy platform and pheatmap in R were applied, with expression data in TPM (Transcripts Per Million) scaled by row (genes) to highlight differential patterns.

The expression of genes associated with the selected GO terms was visualized using a combined heatmap, which incorporated samples from the human heart, grafts in non-human primates (NHPs), and BCT samples. The gene expression counts were scaled across all included samples to enable comparative analysis of their expression profiles.

For analysis of secreted paracrine factors that may contribute to the observed increase of heart function in Amber grafts, expression patterns of genes that are associated with cell secretion (GO terms GO:0046903, GO:0032940, GO:0046887, GO:0050663, GO:0070278, GO:0002793) and with post-myocardial infarction responses as defined by Nguyen et al. [17] were

assessed. Genes annotated to either GO:0005576 (extracellular region) or GO:0005615 (extracellular space) were further classified as secreted.

Tissue sampling, Histology and Immunostaining

All NHP heart samples were taken from defined locations and were categorized and labeled according to a 17-segment model of the human left ventricle [19]. NHP hearts were processed for histology as snap frozen samples or paraffin-embedded samples. Adherent cells were fixed with 2% paraformaldehyde (PFA, Sigma-Aldrich) for 10 min before immunofluorescence staining. Slide-mounted hiCMAs and snap frozen heart tissue sections (both processed by embedding in Tissue-Tek O.C.T Compound (Sakura Finetek Europe) for cryo-sectioning) were fixed with 4% PFA for 4 minutes. Samples were blocked and permeabilized using Tris-buffered saline containing 0.25% Triton-X 100 and 5% donkey serum. Incubation with primary antibodies was performed either for 1 hour at RT or overnight at 4 ˚C. Secondary antibodies were added for 30 min at RT. Both primary and secondary antibodies were diluted in PBS w/o Ca^{2+} and Mg^{2+} + 1% bovine serum albumin (BSA) and used in ratios listed in tables S1, S2 and S3. Nuclei were stained for 15 min with 4',6-diamidino-2-phenylindole (DAPI). Images were taken using an AxioObserver A1 or Z1 fluorescence microscope and analyzed by AxioVision software 4.71 or ZEN software (both Zeiss).

Histomorphometry of tissue sections after immunostaining

Localization of detected grafts in individual NHP heart samples was mapped for each animal to a 17-segment model of the human left ventricle [19]. For quantification, immunohistological images were taken with a 10x objective at the AxioObserver Z1 fluorescence microscope system and processed with ZEN 3.3 software (all Zeiss, Jena, DE). Three images per animal containing at least 1 graft area were imported into Image J software (version 1.53 c) where graft and stained areas (region of interest or ROI) were measured. Graft area was determined based on GFP or RedStar signal expressed by transgene cells that successfully engrafted in monkey hearts. Before area quantification, hyperstack images were split into single channel images with gray

values and 8-bit quality. If necessary, background subtraction, brightness enhancement and median filtering was applied to images. Global threshold was adjusted using the Auto Threshold function and selecting the best fitting option (values: 9 – 199). ROI was selected and surrounding signal was deleted; the remaining area was quantified using the analyze particles function and taking the sum of all. Quantified area of MLC2a and MLC2v signal (μm^2) is represented in correlation to positive graft area (%). Likewise, cardiomyocyte (CM) density in the graft area was determined in defined ROIs as number of nuclei of titin$^+$ cells/mm^2, and endothelial cell (EC) density as number of CD31$^+$ cells /mm^2. The number of nuclei per mm^3 (N_V) was calculated from the number of nuclei per mm^2 (N_A) according to Abercrombie et al. [18] correcting for nuclei diameter (d = 0.01 mm) and sample thickness (t = 0.01 mm) as $N_V = N_A / (d+t)$, i.e. $N_V = 50 \times N_A$.

X-ray µ-CT of paraffin-embedded tissue samples

For 3D imaging, paraffin embedded non-human primate heart tissue samples (from animals #12 and #13) were scanned by Histomography GmbH (Göttingen, Germany) using a laboratory X-ray µ-CT (computed tomography) system optimized for scanning whole paraffin blocks. The 3D volume was reconstructed using a vendor-specific iterative tomographic reconstruction scheme. To enhance image contrast, a phase-retrieval algorithm based on propagation-based X-ray phase-contrast imaging was applied to the 2D projections prior to the tomographic reconstruction. The resulting reconstructed volumes have an isotropic voxel size of 12 µm. The scanned paraffin blocks were then used for subsequent histological analysis and correlative imaging.

After identification of the grafts in the reconstructed 3D volume, segmentation of the grafts was conducted manually by using the segmentation tools within the software webKnossos [20] (Scalable minds UG, Potsdam, Germany). To this end, 2D segments were created every two to five slices, which were then interpolated. For visualization and 3D rendering, webKnossos and the browser-based viewer provided by Histomography GmbH were used.

Statistical analysis

The GraphPad Prism Version 9 or 10 (GraphPad Software, Inc., La Jolla, CA, USA) was used for statistical analyses. Unless indicated otherwise, descriptive statistical analysis was expressed as Mean ± Standard Deviation. Continuous numerical variables were compared using the student's t-test for independent samples, significance was evaluated by two-tailed testing, and assumed at $p<0.05$.

To compare more than two groups, a Two-way ANOVA with Tukey's or Bonferroni's multiple comparisons test was performed. A *p*-value <0.05 was considered statistically significant.

Supplementary Tables

Table S1: primary antibodies (Flow + IF)

Antibody	Isotype	Company	Dilution
ACTN2 (SA)<br>#A7811	mouse IgG1	Sigma-Aldrich, St. Louis, US	1:800
CD31<br>#M0823	mouse IgG1<br>kappa	Dako, Agilent, Santa Clara, US	1:20
cTnI<br>#ab52862	rabbit IgG	Abcam, Cambridge, UK	1:500
CX43<br>#C8093	mouse IgM	Sigma-Aldrich, St. Louis, US	1:1000
GATA4<br>#ab124265	rabbit IgG	Abcam, Cambridge, UK	1:1000
GFP<br># R1091P	goat IgG	Acris, OriGene, Rockville, US	1:500
Ki67<br>#M7240	mouse IgG1	Dako, Agilent, Santa Clara, US	1:100
mCherry<br>#ab167453	rabbit IgG	Abcam, Cambridge, UK	1:500
MLC2a<br>#311011	mouse IgG2b	Synaptic Systems, Göttingen, DE	1:100
MLC2v<br>#ab79935	mouse IgG1	Abcam, Cambridge, UK	1:100
TTN (9 D10)<br>#AB528491	mouse IgM	Hybridoma Bank; University of Iowa, US	1:200 / 1:20<br>(conc.)
TNNT<br>#MS-295-P	mouse IgG1	Thermo Fisher Scientific, Waltham, US	1:100
TNNT<br>#15513-1-AP	rabbit IgG	Proteintech, Manchester, UK	1:500

Table S2: Isotype control antibodies

Antibody	Isotype	Company
Negative Control #X0942	mouse IgM	Dako, Agilent, Santa Clara, US
Negative Control #ab37415	rabbit IgG	Abcam, Cambridge, UK
Negative Control #X0943	mouse IgG2a	Dako, Agilent, Santa Clara, US
Negative Control #X0944	mouse IgG2b	Dako, Agilent, Santa Clara, US
Negative Control #X0931	mouse IgG1	Dako, Agilent, Santa Clara, US
Negative Control #ab37373	Goat IgG	Abcam, Cambridge, UK

Table S3: Secondary antibodies

Host Species and Reactivity	Conjugation	Company	Dilution
Donkey anti-mouse IgG #ab175658	Alexa Fluor 405	Abcam, Cambridge, UK	1:2000
Donkey anti-mouse IgG #715-605-151	Alexa Fluor 647	Dianova, Hamburg, DE	1:300
Donkey anti-mouse IgG #715-165-150	Cy3	Dianova, Hamburg, DE	1:300
Donkey anti-mouse IgG #715-545-151	Alexa Fluor 488	Dianova, Hamburg, DE	1:300
Donkey anti-mouse IgM #715-605-140	Alexa Fluor 647	Dianova, Hamburg, DE	1:300
Donkey anti-mouse IgM #715-485-020	DyLight 488	Dianova, Hamburg, DE	1:200
Donkey anti-rabbit IgG #ab175651	Alexa Fluor 405	Abcam, Cambridge, UK	1:2000
Donkey anti-rabbit IgG #711-505-152	DyLight 549	Dianova, Hamburg, DE	1:300
Donkey anti-rabbit IgG #711-605-152	Alexa Fluor 647	Dianova, Hamburg, DE	1:300
Donkey anti-rabbit IgG #711-545-152	Alexa Fluor 488	Dianova, Hamburg, DE	1:300
Donkey anti-goat IgG #705-495-147	DyLight 649	Dianova, Hamburg, DE	1:300

Supplementary Figures

Figure S1

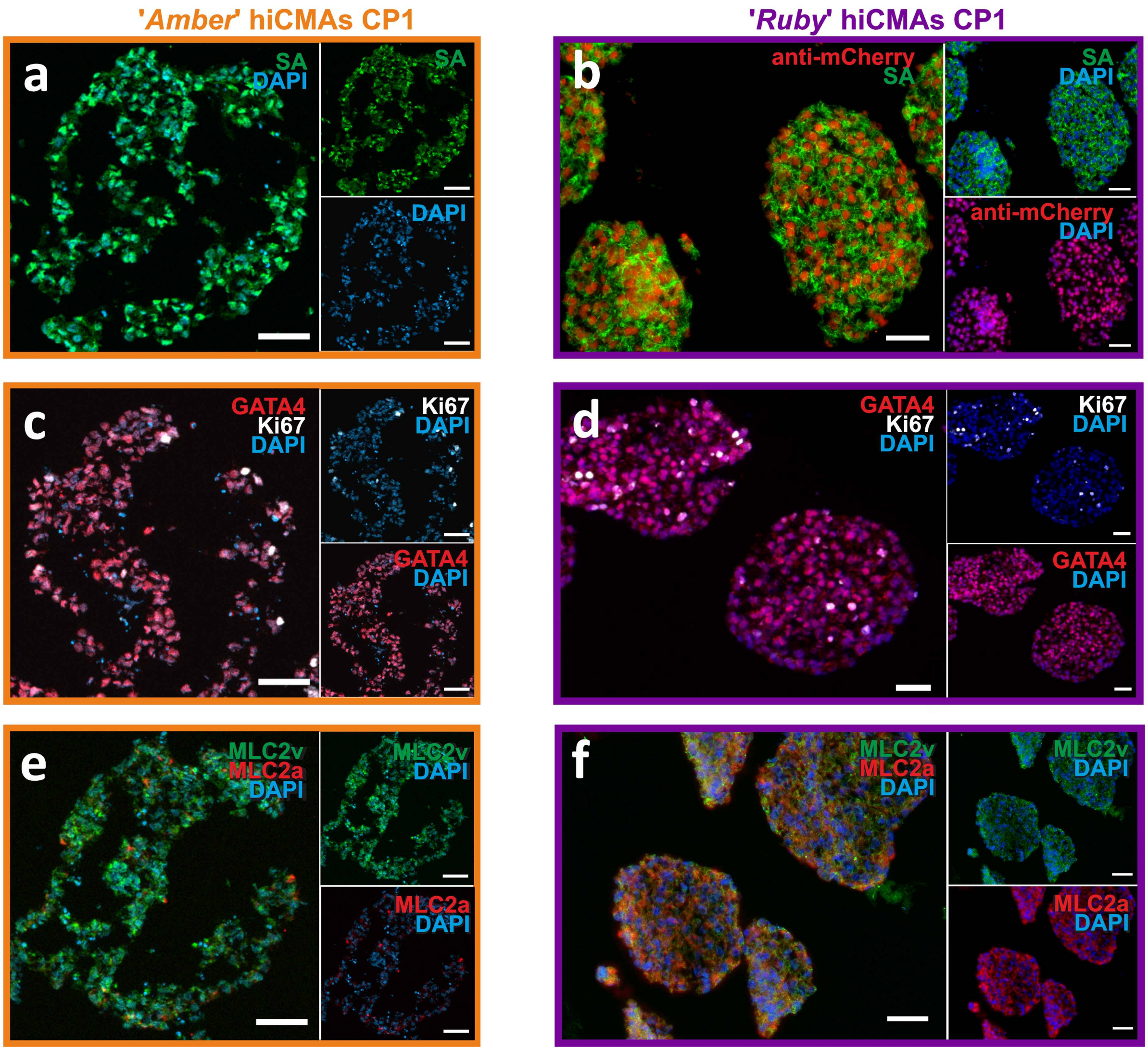

Figure S1 continued

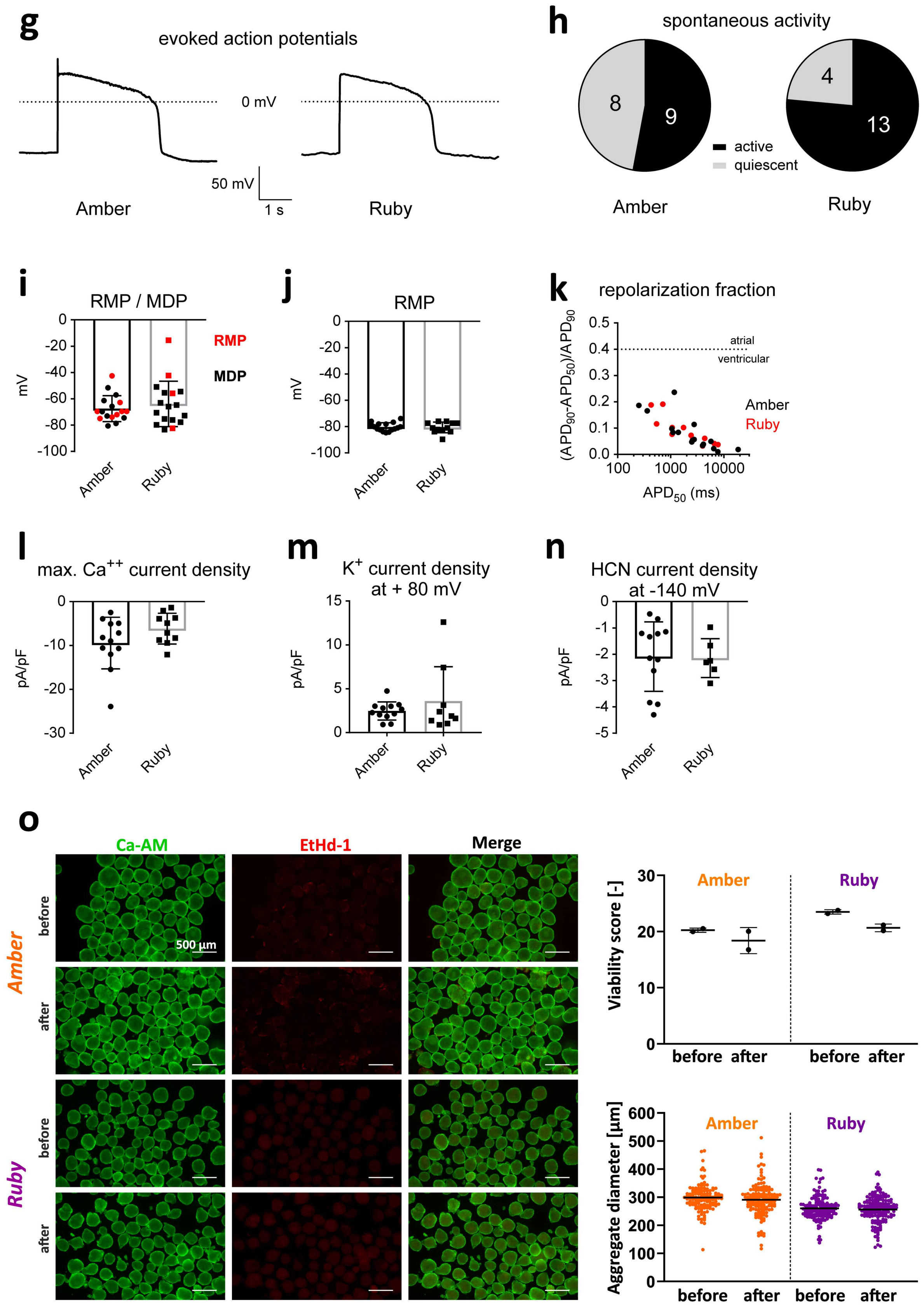

Figure S1: hiPSC-derived enriched cardiac aggregates for transplantation express cardiac markers at CP1, remain intact after passing through a 25G needle and show distinct electrophysiological properties of CMs from *'Amber'* and *'Ruby'* cell lines.

a-f) Immunofluorescence (IF) analysis of cryosections of representative *'Amber'* (**a, c, e**) and *'Ruby'* (**b, d, f**) hiCMAs for 6 Amber and 3 Ruby hiCMA batches from CP1 for expression of α-sarcomeric actinin (SA), and nuclear RedStar (stained with anti-mCherry mAb) in **b**. **c, d**) IF revealed comparable expression of GATA4 and Ki-67 (Ki67) in *'Amber'* and *'Ruby'* hiCMAs. **e, f)** Cryosections of representative hiCMAs show both ventricular and atrial myosin light chain (MLC2v and MLC2a) expression with double positive cells indicating immature CMs. Nuclei stained with DAPI (blue). Scale bars: 100 µm. **g)** Electrophysiological properties of hiPSC-derived CMs from *'Amber'* or *'Ruby'* hiPSCs were compared at day 2-4 after seeding on glass coverslips, using the whole-cell patch clamp technique. Representative action potentials of an Amber-CM and Ruby-CM starting from a resting potential of -80 mV. Responses were evoked by short depolarizing stimuli (Amber: 1 ms, 400 pA; Ruby: 1 ms, 600 pA). The rectangular shape of such action potentials indicated a ventricular-like phenotype for all CMs of this study. **h)** Nine out of 17 *'Amber'*-CMs spontaneously generated action potentials. The remaining cells were quiescent, but could be activated by short depolarizing stimuli. The majority of *'Ruby'*-CMs (13 out of 17 cells) was spontaneously active**. i)** Scatter plot of resting membrane potential (RMP for quiescent CMs) and maximum diastolic potential (MDP for spontaneously active CMs). **j)** Resting membrane potentials experimentally adjusted close to -80 mV via injection of small holding currents (Amber: n = 16; Ruby: n = 12). RMP of quiescent CMs or MDP did not differ between cell lines. **k)** Repolarization fraction of action potentials from Amber-CMs and Ruby-CMs plotted versus the action potential duration at 50% repolarization level (APD50) (Amber: n = 16; Ruby: n = 12) confirmed the ventricular phenotype. **l-n)** Comparison of maximum calcium current densities (l; Amber: n = 12; Ruby: n = 10), steady state potassium current densities at +80 mV (m; Amber: n = 12; Ruby: n = 9), and HCN current densities at -140 mV (n; Amber: n = 12; Ruby: n = 6) showed no significant differences (p>0.05). Data as mean ± SEM with significance levels from Student's t-test. o) Exemplary fluorescence microscopic analysis of *'Amber'* and *'Ruby'* hiCMAs stained with Calcein AM (Ca-AM´, viability) and Ethidium Homodimer 1 (EthD-1, dead cells) collected at CP2 before and after being passed through a 26G syringe (left side). Comparison of the viability score (top right; n=2 images analysed) and the aggregate size distribution (bottom right; n=128-155 aggregates analysed; differences were not significant with One-way ANOVA with Bonferroni's multiple comparison test). Source data are provided as a Source Data file.

Figure S2

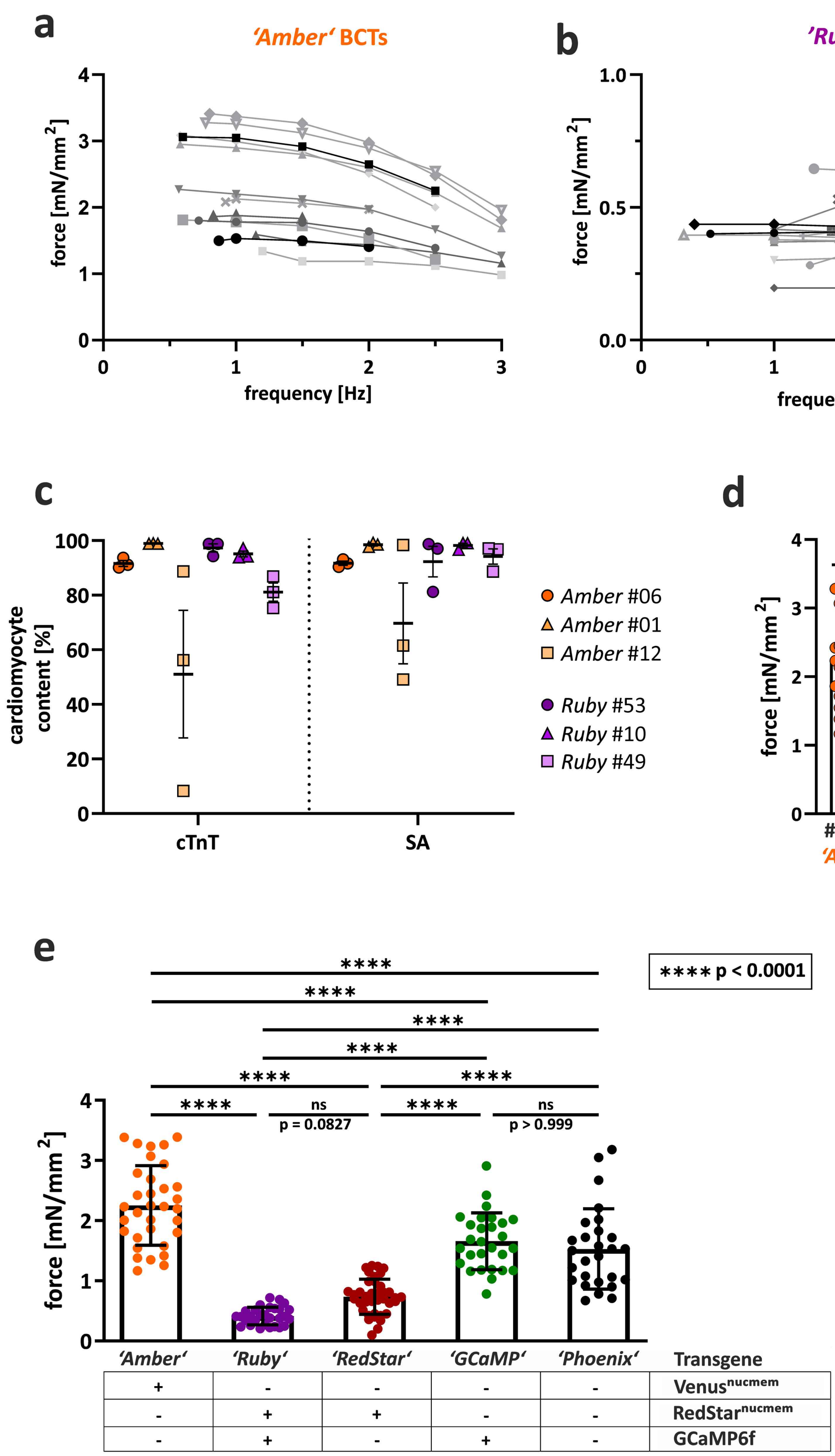

a
'Amber' BCTs
force [mN/mm2]
frequency [Hz]
b
'Ruby' BCTs
force [mN/mm2]
frequency [Hz]
c
cardiomyocyte content [%]
cTnT
SA
Amber #06
Amber #01
Amber #12
Ruby #53
Ruby #10
Ruby #49
d
p = 0.0062
force [mN/mm2]
#06 #01 #12
#53 #10 #49
'Amber' BCTs
'Ruby' BCTs
e
**** p < 0.0001
ns
p = 0.0827
p > 0.999
force [mN/mm2]
'Amber'
'Ruby'
'RedStar'
'GCaMP'
'Phoenix'
Transgene
Venusnucmem
RedStarnucmem
GCaMP6f

Figure S2: Extended characterization of contractions forces of hiPSC-derived BCTs from *'Amber'* and *'Ruby'* cell lines

a, b) Force-frequency relationship was determined for hiPSC-derived BCTs from *'Amber'* and *'Ruby'* cell lines at maximum preload by biphasic stimulation of tissues between 1 and 3 Hz for the duration of 20 sec on d14 of cultivation (matching the first time-point of *in vivo* analysis after cell transplantation). The observed negative or flat force-frequency-relationship (FFR) for *'Amber'* or *'Ruby'* BCTs, respectively, indicates an immature phenotype the tissue [21] and is consistent with the early time-point of measurement. **c)** To investigate the contractility of *'Amber'* and *'Ruby'* iPSC lines on a clonal level, two additional clonal line for *'Amber'* (#01 and #12) and *'Ruby'* (#10 and #59) were differentiated. Flow cytometry indicated a typical CM content of >90% after staining of dissociated hiCMAs for cardiac troponin T (cTnT) and α-sarcomeric actinin (SA); with lower cardiomyocyte content for *'Amber'* clone #12 and 'Ruby' clone #49. Differences between individual clones were not significant with One-way ANOVA with Bonferroni's multiple comparison test. **d)** On d14, maximum active forces of BCTs normalized to the cross-sectional area show significantly higher forces for all *'Amber'*-BCTs vs. *'Ruby'*-BCTs (2.1 ± 0.6 mN/mm^2 vs. 0.4 ± 0.2 mN/mm^2, Mean ± SD for 3 experiments with n=10-40 BCTs each; * p=0.0062 for nested t-test).

e) Comparative contraction force analysis for BCTs with different transgenes, i. e. *'Amber'* BCTs expressing Venus and the double-transgenic 'Ruby' BCTs expressing both RedStar and GCamp6f as well as BCTs from the respective single-transgenic and non-transgenic iPSC lines. '*Phoenix*' (the non-transgenic mother line of '*Amber*' and '*Ruby*'), a CaMP6f-expressing line, and an iPSC line that just expresses nuclear RedStar (both also '*Phoenix*'-derived). All other BCTs demonstrated significantly higher active forces than those derived from the two RedStar expressing lines '*Ruby*' and '*RedStar*'. Mean ± SD for 3-4 experiments with n=27-38 BCTs each; ns, not significant; **** p<0.0001 for One-way ANOVA with Bonferroni's multiple comparisons test. Source data are provided as a Source Data file.

Figure S3

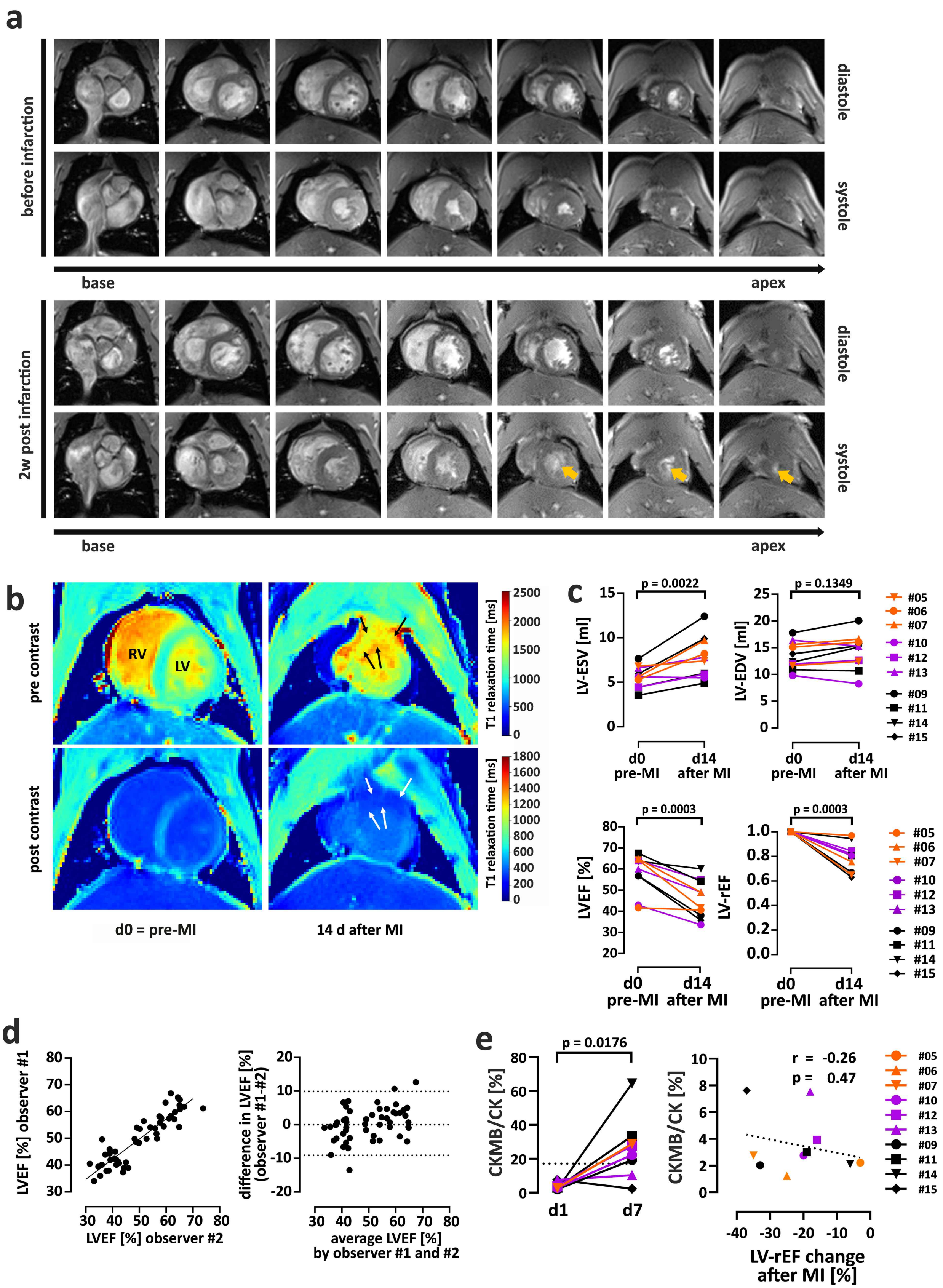

a
before infarction
diastole
systole
base
apex
2w post infarction
diastole
systole
base
apex
b
pre contrast
post contrast
RV
LV
T1 relaxation time [ms]
d0 = pre-MI
14 d after MI
c
p = 0.0022
LV-ESV [ml]
p = 0.1349
LV-EDV [ml]
d0 pre-MI
d14 after MI
#05
#06
#07
#10
#12
#13
#09
#11
#14
#15
p = 0.0003
LVEF [%]
p = 0.0003
LV-rEF
d
LVEF [%] observer #1
LVEF [%] observer #2
difference in LVEF [%] (observer #1-#2)
average LVEF [%] by observer #1 and #2
e
p = 0.0176
CKMB/CK [%]
d1
d7
r = -0.26
p = 0.47
LV-rEF change after MI [%]

Figure S3: Cardiac MRI assessment of the infarcted myocardium showed impaired function and infarct localization

Cardiac MRI was performed to measure left ventricular end-systolic volume (LV-ESV, left panel) and left ventricular end-diastolic volume (LV-EDV, right panel) pre-MI and on d14 after MI for individual animals. **a)** Short-axis images of selected slices of monkey #09 before and 2 weeks after myocardial infarction (MI) from base to apex. A significantly larger systolic volume in comparison to the non-infracted heart is clearly visible on the apical slices 2 weeks after MI (see arrows) and shows loss of systolic contractility. **b)** Short-axis view of representative MRI T1 relaxation maps of the myocardium from healthy monkey #09 before myocardial infarction (pre-MI) and 14 days after infarction demonstrate localized changes in tissue composition. Increased native (pre-contrast, top) and markedly reduced post-contrast (bottom) T1 relaxation times delineate the fibrotic scar (black and white arrows) located in the septal and apical regions of the myocardium. **c)** The increase of LV-ESV after MI was significant (7.8 ± 2.4 ml vs. 5.6 ± 1.2 ml before MI; p = 0.0022 for n=10, two-tailed paired-t-test), while LV-EDV did not change significantly (see table 1 for individual data). Consequently, LV-EF decreased as well from 58.2 ± 9.1 % to 45.7 ± 9.0 % (p=0.0003 for n=10, two-tailed paired t-test). Due to the large variance of pre-MI LVEF, relative LVEF values normalized to pre-MI values (=1.0) were used for comparative analyses. For all animals in the functional analysis, LV-rEF after MI was reduced to 0.79 ± 0.12 after MI (p=0.0003 for n=10, two-tailed paired t-test). The infarcted animals were assigned to the treatment groups A and B1 (treatment with hiCMA '*Amber'*, orange symbols), group B2 (treatment with hiCMA '*Ruby'*, purple symbols), and group C (sham, black). **d)** Good inter-observer agreement was achieved for the left ventricular ejection fraction (LVEF) calculated from LV-ESV and LV-EDV; left panel), the bias (difference between the means) was only 0.4 and the intra-class correlation coefficient (ICC) 0.89. Bland-Altman plot shows the inter-observer variability with 95% limits of agreement between -9.086 and 9.890.

e) Serum levels of clinically used infarction markers creatine kinase (CK), and creatine kinase MB isoenzyme (CK-MB) were determined 1 and 7 days after coronary artery ligation in animal groups B1, B2, and C. The CK-MB ratio (or CK-MB relative index) is reduced 1d after MI compared to d7 (p = 0.0176 with two-tailed t-test) and mean levels measured in healthy control animals unrelated to this study (dotted line, n=6). However, CK-MB ratio on d1 did not correlate significantly with the functional impairment of cardiac contractility on day 14 assessed by MRI (LV-rEF change after MI [%] = (LV-rEF d14 – LV-rEF d0)*100) for groups B1, B2, and C (Pearson correlation coefficient (r) and p value for two-tailed analysis with n=10). Source data are provided as a Source Data file.

Figure S4

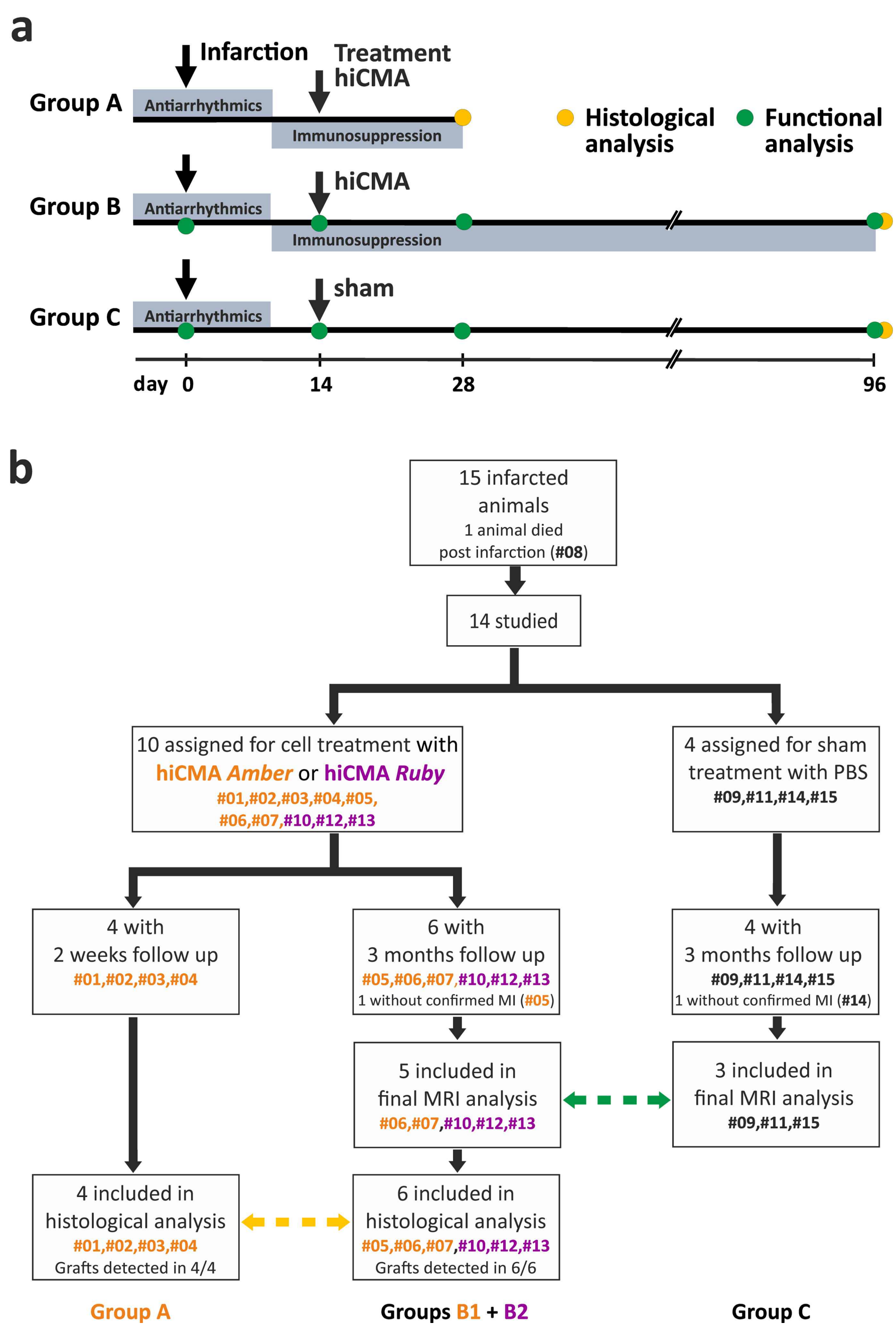

Figure S4: Animal study design

a) Time line for interventions and analyses. The simplified scheme shows i) time points of major interventions, ii) timing of medication with anti-arrhythmics and immunosuppressants, and iii) time points for functional analysis by MRI and histological analysis for the different experimental groups A, B and C.

b) Flow diagram for animal assignment to treatment groups and inclusion in MRI and histology analysis. The figure depicts the experimental flow, mortality at each stage of the study, assignment of individual animals into experimental groups, and final group sizes for MRI and histology analyses.

Figure S5

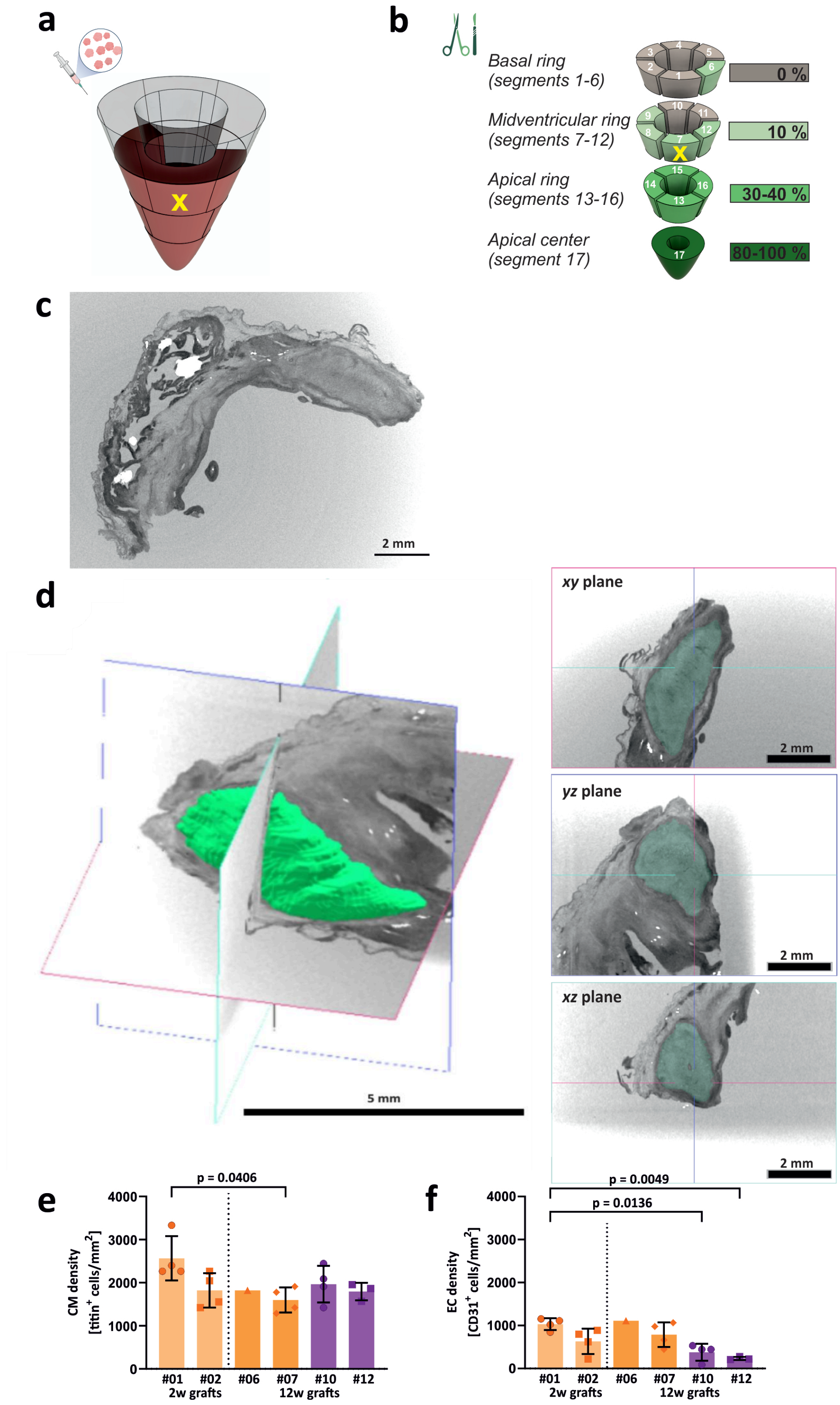

a
b
Basal ring
(segments 1-6)
0 %
Midventricular ring
(segments 7-12)
10 %
Apical ring
(segments 13-16)
30-40 %
Apical center
(segment 17)
80-100 %
c
2 mm
d
5 mm
xy plane
2 mm
yz plane
2 mm
xz plane
2 mm
e
p = 0.0406
CM density
[titin+ cells/mm2]
#01
#02
#06
#07
#10
#12
2w grafts
12w grafts
f
p = 0.0049
p = 0.0136
EC density
[CD31+ cells/mm2]
#01
#02
#06
#07
#10
#12
2w grafts
12w grafts

Figure S5: Graft localization and quantification via histomorphometry or X-ray µ-CT of paraffin-embedded tissue samples.

a) Graphical summary of injection site localization. All injections were performed under visual control after re-thoracotomy to target the left-ventricular region apical to the coronary artery ligation. Injection sites were documented for each animal in a schematic illustration of the standardized 17-segment model of the human left ventricle (the illustration was created for this work; left ventricular segment nomenclature follows the recommendations of Cerqueira et al. [19]; red: injection area; yellow X: position of ligation). **b)** Graphical summary of graft localization. All NHP heart samples were taken from defined locations and were categorized and labeled according to the 17-segment model. Yellow X marks the position of the ligation. The scheme shows the frequency of iPSC-derived graft detection in individual segments for all 10 animals injected with hiCMAs from groups A and B (% of animals with grafts in the respective segment). **c)** 3D imaging of paraffin embedded tissue samples using a laboratory X-ray µ-CT; here single plane showing complete tissue sample. **d)** Following correlative identification of the grafts and manual segmentation in 2D, 3D reconstruction for volumetric analysis showed a substantial 'Ruby' graft in animal #13 with a volume of 9.2 mm3; scale bar 5 mm. **e)** CM density was quantified via histomorphometry and was similar for both cell lines 'Amber' and 'Ruby' in all grafts investigated 3 months after injection with a mean of 1,795 ± 151 CM/mm2 (determined as number of nuclei of titin+ cells in the graft area). p=0.0406 for One-way ANOVA with Bonferroni's multiple comparisons test. **f)** Endothelial cell density was quantified via histomorphometry as number of CD31+ cells in the graft area and appeared to be lower in 'Ruby' grafts compared to 'Amber' grafts (both 3 months after injection), albeit not statistically significant. Significant differences were observed for comparison of 'Ruby' grafts 3 months after injection to 'Amber' grafts 2 weeks after injection; One-way ANOVA with Bonferroni's multiple comparisons test. Source data are provided as a Source Data file.

Figure S6

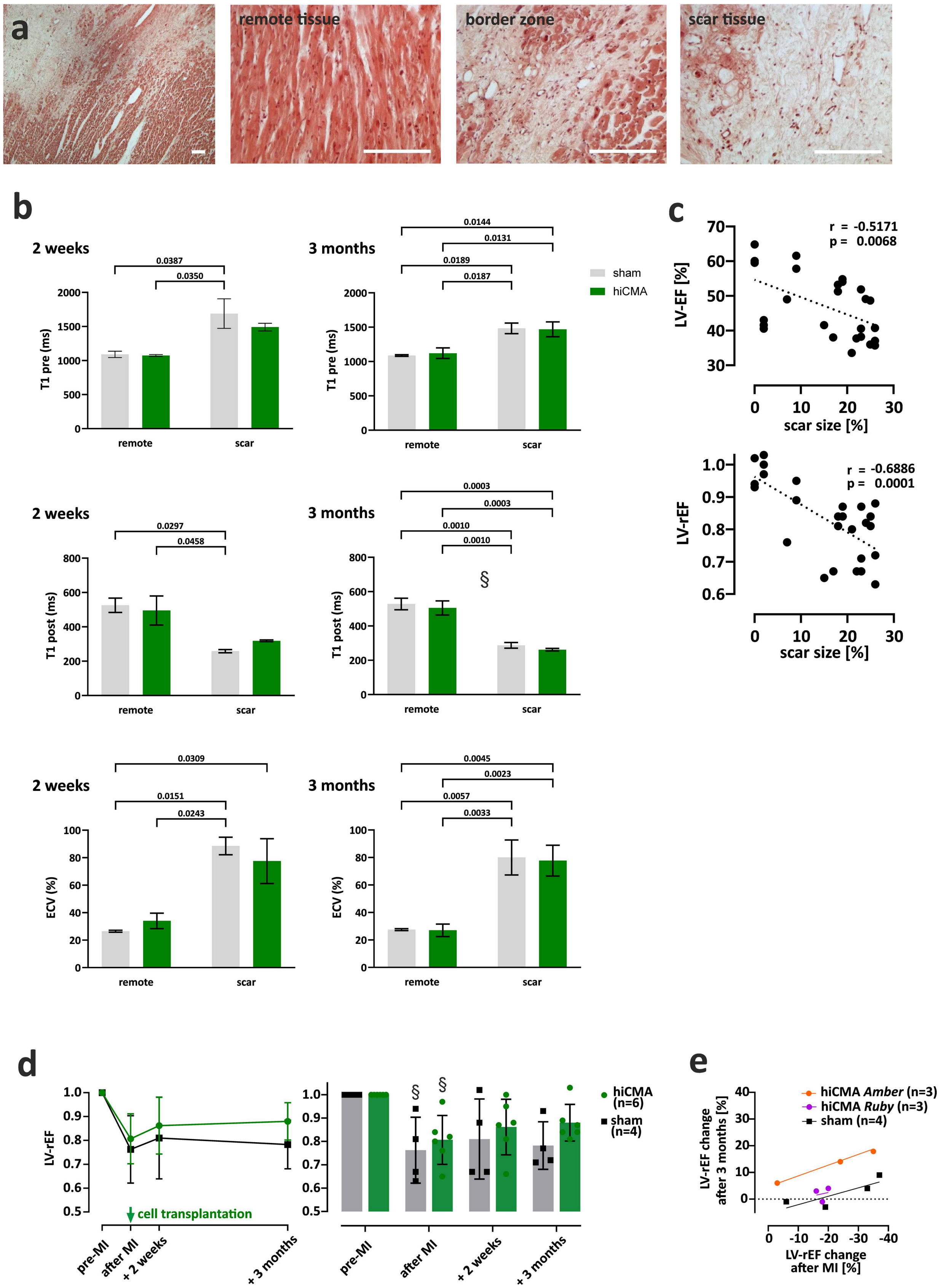
a
remote tissue
border zone
scar tissue
b
2 weeks
3 months
sham
hiCMA
T1 pre (ms)
T1 post (ms)
ECV (%)
remote
scar
0.0387
0.0350
0.0144
0.0131
0.0189
0.0187
0.0297
0.0458
0.0003
0.0003
0.0010
0.0010
§
0.0309
0.0151
0.0243
0.0045
0.0023
0.0057
0.0033
c
LV-EF [%]
r = -0.5171
p = 0.0068
scar size [%]
LV-rEF
r = -0.6886
p = 0.0001
scar size [%]
d
LV-rEF
cell transplantation
pre-MI
after MI
+ 2 weeks
+ 3 months
§
hiCMA (n=6)
sham (n=4)
e
LV-rEF change after 3 months [%]
hiCMA Amber (n=3)
hiCMA Ruby (n=3)
sham (n=4)
LV-rEF change after MI [%]

Figure S6: Extended cardiac MRI data assessment and scar tissue characterization after up to 3 months

a) Microscopic pictures after HE staining confirmed the presence of infarcted myocardium and scar tissue formation in an untreated animal (#09) after 3 months. Scale bars 200 µm. **b)** Myocardial tissue characteristics and fibrotic changes were assessed by cardiac MRI, i. e. T1-relaxation times of scar and remote myocardium prior and 15 minutes after contrast agent administration. The scar regions showed an increase in the pre-contrast (top) and a decrease in the post-contrast (middle) T1-relaxation time compared to the remote region as expected after tissue injury. No significant difference could be observed between the treated and untreated animals, neither 2 weeks nor 3 months after treatment. The scar tissue showed an increased extracellular volume (ECV) independent of the treatment (bottom), demonstrating the presence of fibrotic tissue in all animals. Adjusted p values are depicted for all significant differences ($p < 0.05$ with Two-way ANOVA and Bonferroni's multiple comparison test). **c)** Correlation analyses of scar size and LV-EF & LV-rEF, all assessed by MRI for groups B1, B2, and C (Pearson correlation coefficient (r) and p value for two-tailed analysis). **d)** Due to the large variance of pre-MI LVEF in cardiac MRI measurements, LV-rEF values normalized to pre-MI values were used to assess the outcome of individual hiCMA-treated animals. For both the sham control group C (black symbols, n= 4; #09, #11, #14, #15) and the combined treatment groups B1+B2 (green symbols, n=6; #05, #06, #07, #10, #12, #13) a significant reduction of LV-rEF was measured after MI (§, p=0.0437 and p=0.0062, respectively, compared to respective pre-MI with paired t-test). For the combined assessment of treatment groups B1+B2 a trend for increased LVEF was observed after 3 months, but did not reach statistical significance (0.88 ± 0.08 vs. 0.78 ± 0.10 for the control group; adjusted p value = 0.33 with unpaired t-test and multiple comparison with the Holm-Sidák method; left panel). The right panel displays the large variation of values within groups, most probably preventing statistical significance. Notably, the two animals with very small LV-EF changes after MI (animals #05 and #14, Fig. 3c) could be identified as outliers after 2 weeks and 3 months as well, because they did not show major changes after treatment or sham. **e)** Re-analysis of the MRI data showed that the observed functional improvement (LV-rEF change after 3 months) appears to be correlated to the initial decrease in function (LV-rEF change 2 weeks after MI) and is clearly dependent on the treatment group. A slight recovery was observed for sham treated animals after large impairment (>30% LV-rEF reduction), this was clearly outperformed after treatment with hiCMA from MHHi0001-A-11 '*Amber'* (as can be seen by the difference in elevation of the linear regression), but not after treatment with hiCMA from MHHi0001-A-5 'Ruby'. Source data are provided as a Source Data file.

Figure S7

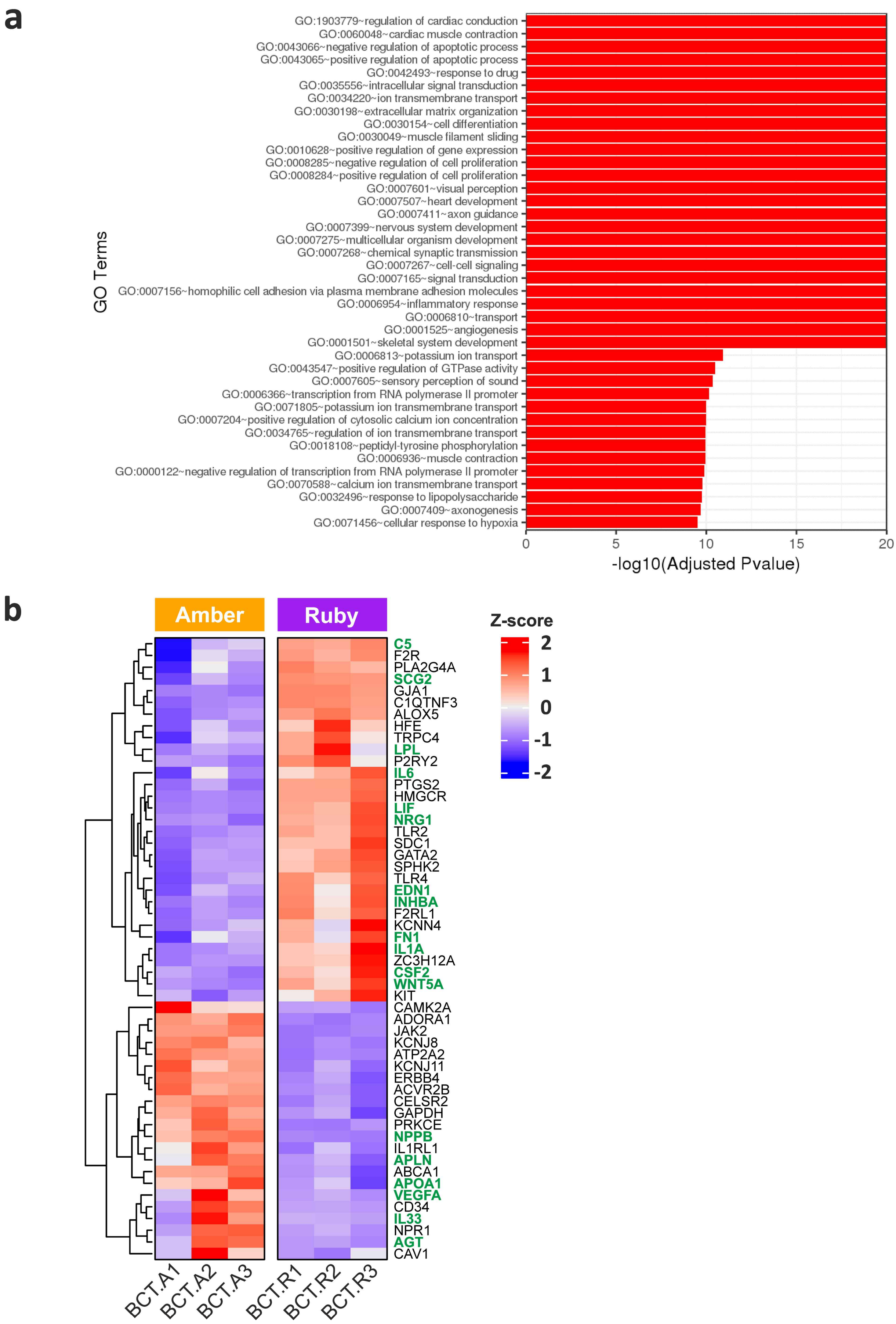
a
GO Terms
GO:1903779~regulation of cardiac conduction
GO:0060048~cardiac muscle contraction
GO:0043066~negative regulation of apoptotic process
GO:0043065~positive regulation of apoptotic process
GO:0042493~response to drug
GO:0035556~intracellular signal transduction
GO:0034220~ion transmembrane transport
GO:0030198~extracellular matrix organization
GO:0030154~cell differentiation
GO:0030049~muscle filament sliding
GO:0010628~positive regulation of gene expression
GO:0008285~negative regulation of cell proliferation
GO:0008284~positive regulation of cell proliferation
GO:0007601~visual perception
GO:0007507~heart development
GO:0007411~axon guidance
GO:0007399~nervous system development
GO:0007275~multicellular organism development
GO:0007268~chemical synaptic transmission
GO:0007267~cell-cell signaling
GO:0007165~signal transduction
GO:0007156~homophilic cell adhesion via plasma membrane adhesion molecules
GO:0006954~inflammatory response
GO:0006810~transport
GO:0001525~angiogenesis
GO:0001501~skeletal system development
GO:0006813~potassium ion transport
GO:0043547~positive regulation of GTPase activity
GO:0007605~sensory perception of sound
GO:0006366~transcription from RNA polymerase II promoter
GO:0071805~potassium ion transmembrane transport
GO:0007204~positive regulation of cytosolic calcium ion concentration
GO:0034765~regulation of ion transmembrane transport
GO:0018108~peptidyl-tyrosine phosphorylation
GO:0006936~muscle contraction
GO:0000122~negative regulation of transcription from RNA polymerase II promoter
GO:0070588~calcium ion transmembrane transport
GO:0032496~response to lipopolysaccharide
GO:0007409~axonogenesis
GO:0071456~cellular response to hypoxia
0
5
10
15
20
-log10(Adjusted Pvalue)
b
Amber
Ruby
Z-score
2
1
0
-1
-2
C5
F2R
PLA2G4A
SCG2
GJA1
C1QTNF3
ALOX5
HFE
TRPC4
LPL
P2RY2
IL6
PTGS2
HMGCR
LIF
NRG1
TLR2
SDC1
GATA2
SPHK2
TLR4
EDN1
INHBA
F2RL1
KCNN4
FN1
IL1A
ZC3H12A
CSF2
WNT5A
KIT
CAMK2A
ADORA1
JAK2
KCNJ8
ATP2A2
KCNJ11
ERBB4
ACVR2B
CELSR2
GAPDH
PRKCE
NPPB
IL1RL1
APLN
ABCA1
APOA1
VEGFA
CD34
IL33
NPR1
AGT
CAV1
BCT.A1
BCT.A2
BCT.A3
BCT.R1
BCT.R2
BCT.R3

Figure S7: a) GO functional analysis of DEGs. Significantly enriched GO terms in BCTs derived from 'Ruby' and 'Amber' hiPSC lines as analyzed with GeneSCF v.1.1-p2, clustering genes according to biological processes with goa_human as the GO list. The 40 most significantly ($P<0.05$) enriched GO terms are presented. All the adjusted statistically significant values of the terms were negative 10-base log transformed. GO:0060048 (cardiac muscle contraction) and GO:0030049 (muscle filament sliding) are among the most significantly enriched terms. DEGs, differentially expressed genes; GO, gene ontology. **b)** Differential expression of paracrine factors in 'Amber' vs. 'Ruby' hiCMAs and in BCTs derived from both lines analyzed by RNA sequencing. Expression patterns of genes that are associated with cell secretion (GO terms GO:0046903, GO:0032940, GO:0046887, GO:0050663, GO:0070278, GO:0002793) and with post-myocardial infarction responses as defined by Nguyen et al. [17] were assessed. Among those genes, 12 ones encoding for secreted factors (highlighted in green font; including IL6, LIF, EDN1, SCF2, WNT5A and others) were upregulated in 'Ruby' and 6 (i. e. NPPB, APLN, APOA1, VEFGA, IL33, and AGT) in the respective 'Amber' samples. Color scale represents row-wise Z-score-scaled TPM values.

Figure S8

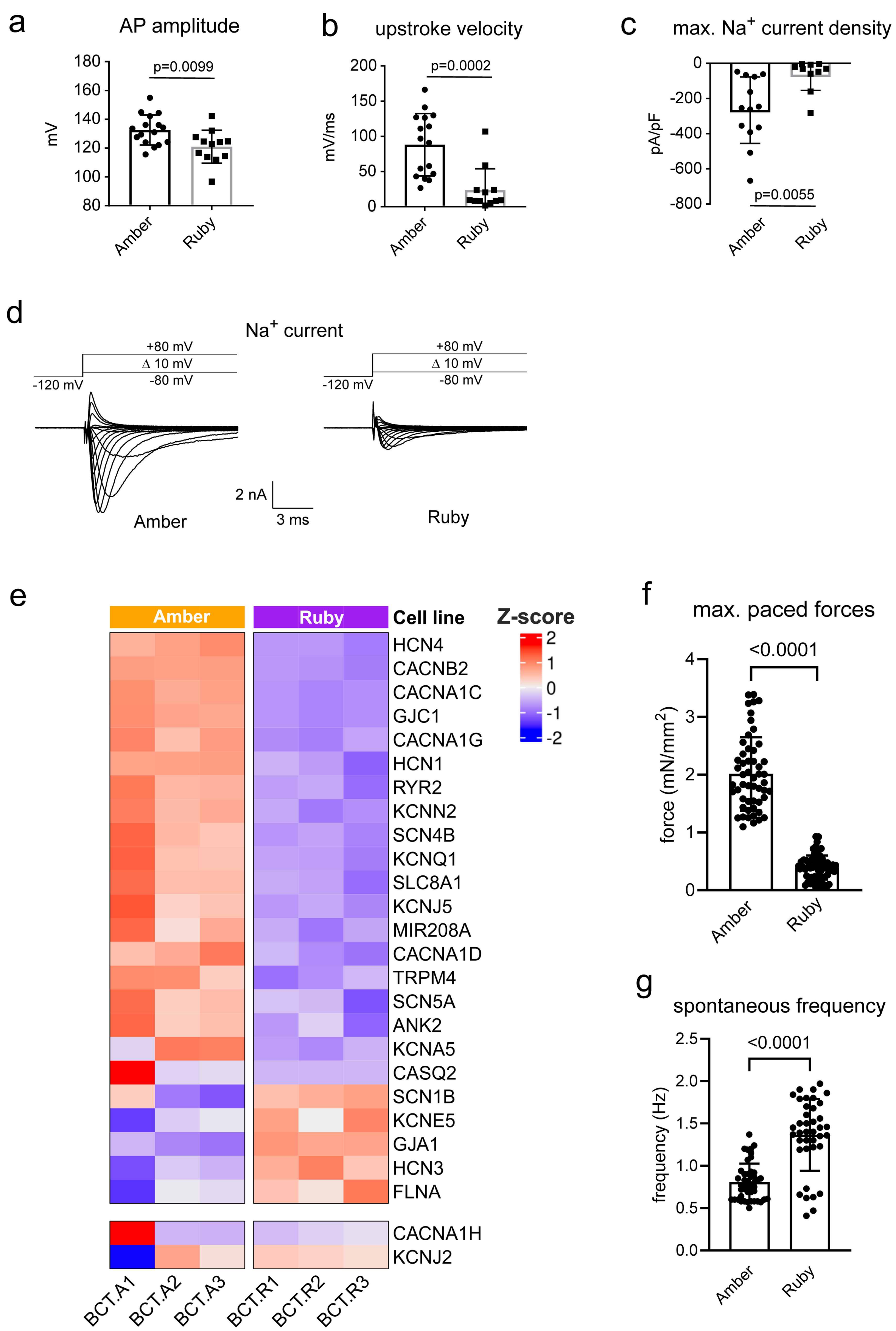
a
AP amplitude
p=0.0099
mV
Amber
Ruby
b
upstroke velocity
p=0.0002
mV/ms
Amber
Ruby
c
max. Na+ current density
p=0.0055
pA/pF
Amber
Ruby
d
Na+ current
+80 mV
Δ 10 mV
-80 mV
-120 mV
2 nA
3 ms
Amber
Ruby
e
Amber
Ruby
Cell line
Z-score
HCN4
CACNB2
CACNA1C
GJC1
CACNA1G
HCN1
RYR2
KCNN2
SCN4B
KCNQ1
SLC8A1
KCNJ5
MIR208A
CACNA1D
TRPM4
SCN5A
ANK2
KCNA5
CASQ2
SCN1B
KCNE5
GJA1
HCN3
FLNA
CACNA1H
KCNJ2
BCT.A1
BCT.A2
BCT.A3
BCT.R1
BCT.R2
BCT.R3
f
max. paced forces
<0.0001
force (mN/mm2)
Amber
Ruby
g
spontaneous frequency
<0.0001
frequency (Hz)
Amber
Ruby

Figure S8: Differential expression of cardiac conduction-associated genes and electrophysiological properties in *'Amber'* vs *'Ruby'* CMs. (a, b) Scatter plot of action potential amplitudes (a) and upstroke velocities (b) ('*Amber'*: n = 16; '*Ruby'*: n = 12). Action potentials of *'Amber'*-CMs were significantly larger due to a faster upstroke velocity and larger initial overshoot. **c, d)** Maximum sodium current densities (c; '*Amber'*: n = 13; '*Ruby'*: n = 10) and representative sodium current traces in response to a voltage step protocol as indicated (d) confirmed significantly larger sodium currents for the *'Amber'*-CMs in comparison to CMs from the *'Ruby'* cell line. Data are presented as mean ± SD with significance levels analyzed by unpaired two-tailed t-test. **e)** Comparison of gene expression profiles of '*Ruby'*- and '*Amber'*-derived BCTs for the GO term 'cell signaling involved in cardiac conduction'. SCN5A (Sodium channel protein type 5 alpha), SCN3B (Sodium Voltage-Gated Channel Beta Subunit 3), HCN4 (Hyperpolarization-activated Cyclic Nucleotide-gated channel 4), SLC8A1 (Sodium/ Calcium Exchanger 1), CACNA1H (Calcium Voltage-gated Channel Subunit alpha1 H), KCNJ2 (potassium inwardly rectifying channel subfamily J member 2). Color scale represents row-wise Z-score-scaled TPM values. Source data are provided as a Source Data file. **f, g)** Comparison of functional properties of *'Amber'* and *'Ruby'* derived BCTs depicting maximum spontaneous frequency (F; *'Amber'*: 0.81 ± 0.22 Hz; Mean ± SD for n =45; *'Ruby'*: 1.37 ± 0.42 Hz; Mean ± SD for n = 39) and maximum paced force (G; *'Amber'*: 2.01 ± 0.63 mN/mm^2; Mean ± SD for n = 53; *'Ruby'*: 2.01 ± 0.63 mN/mm^2; Mean ± SD for n = 60). Significance levels were analyzed by unpaired two-tailed t-test.

Figure S9

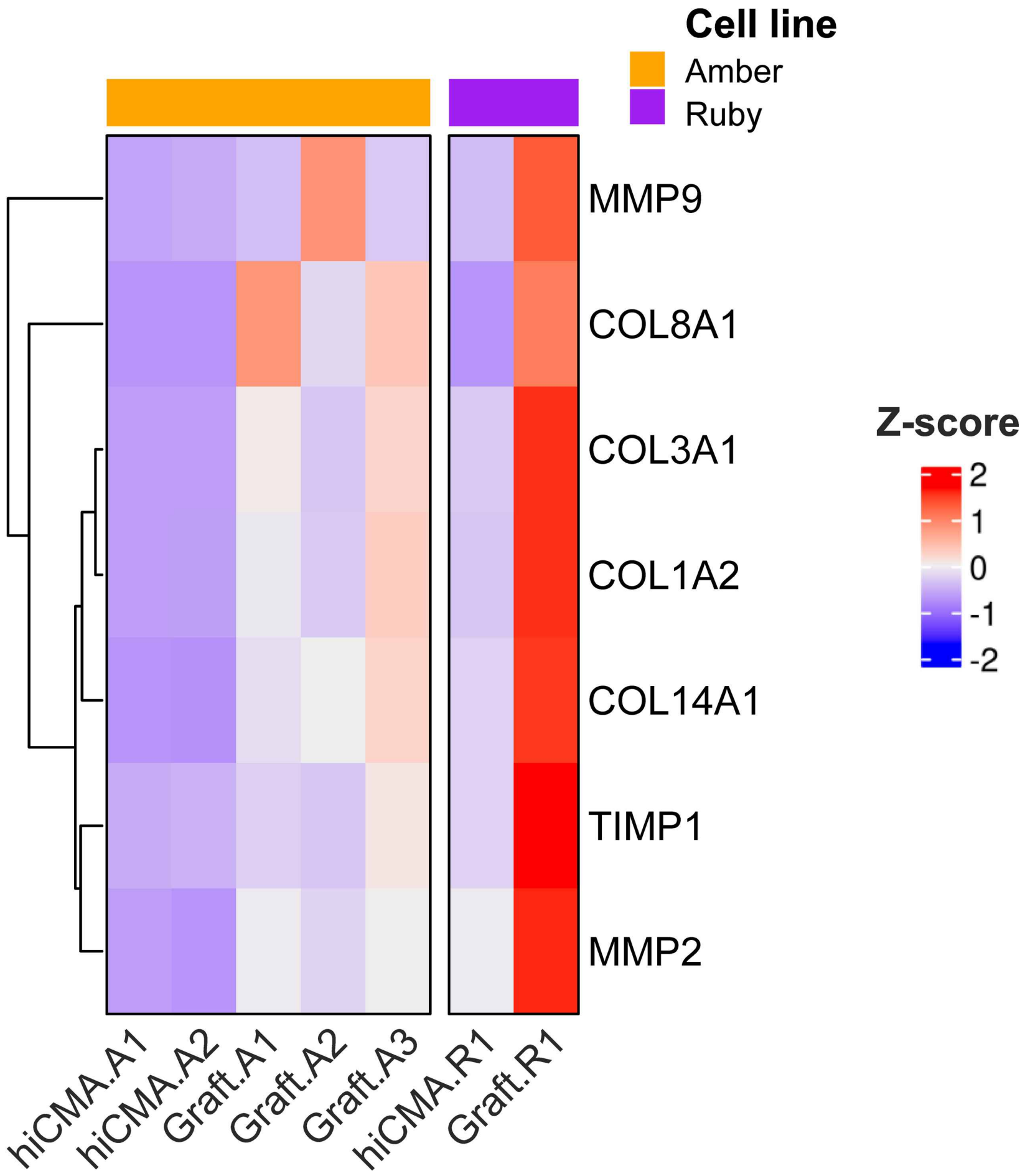

Figure S9: Differential expression of genes associated with reporter gene-induced fibrosis and impaired protein degradation. Comparison of gene expression profiles of '*Ruby*'- and '*Amber*'-derived hiCMAs and grafts show differential expression of fibrosis-related genes that have been reported previously by Chen et al. in the hearts of mice overexpressing the red fluorescent protein (RFP) DsRed, resulting in fibrosis and severe cardiac dysfunction [22]. Interestingly, we found reminiscent gene expression patterns in the *'Ruby'* graft in animal #10, potentially hinting toward a similar mechanism of RFP-mediated cardiac toxicity in RedStar expressing *'Ruby'*-CMs, -BCTs and -cardiac grafts as well. Color scale represents row-wise Z-score-scaled TPM values.

Supplementary References

1. Halloin C*, et al.* Continuous WNT Control Enables Advanced hPSC Cardiac Processing and Prognostic Surface Marker Identification in Chemically Defined Suspension Culture. *Stem Cell Reports* **13**, 775 (2019).

2. Manstein F*, et al.* High density bioprocessing of human pluripotent stem cells by metabolic control and in silico modeling. *Stem Cells Transl Med* **10**, 1063-1080 (2021).

3. Nagai T, Ibata K, Park ES, Kubota M, Mikoshiba K, Miyawaki A. A variant of yellow fluorescent protein with fast and efficient maturation for cell-biological applications. *Nat Biotechnol* **20**, 87-90 (2002).

4. Haase A*, et al.* Establishment of MHHi001-A-5, a GCaMP6f and RedStar dual reporter human iPSC line for in vitro and in vivo characterization and in situ tracing of iPSC derivatives. *Stem Cell Res* **52**, 102206 (2021).

5. Haase A, Gohring G, Martin U. Generation of non-transgenic iPS cells from human cord blood CD34(+) cells under animal component-free conditions. *Stem Cell Res* **21**, 71-73 (2017).

6. Ricci Signorini ME*, et al.* Generation of human induced pluripotent stem cell lines encoding for genetically encoded calcium indicators RCaMP1h and GCaMP6f. *Stem Cell Res* **60**, 102697 (2022).

7. Palecek J, Zweigerdt R, Olmer R, Martin U, Kirschning A, Drager G. A practical synthesis of Rho-Kinase inhibitor Y-27632 and fluoro derivatives and their evaluation in human pluripotent stem cells. *Org Biomol Chem* **9**, 5503-5510 (2011).

8. Kensah G*, et al.* Murine and human pluripotent stem cell-derived cardiac bodies form contractile myocardial tissue in vitro. *Eur Heart J* **34**, 1134-1146 (2013).

9. Tian Y, Martinez MM, Pappas D. Fluorescence correlation spectroscopy: a review of biochemical and microfluidic applications. *Appl Spectrosc* **65**, 115A-124A (2011).

10. Kriedemann N*, et al.* Standardized production of hPSC-derived cardiomyocyte aggregates in stirred spinner flasks. *Nat Protoc*, (2024).

11. Eiringhaus J*, et al.* Performance and feasibility of three different approaches for computer based semi-automated analysis of ventricular arrhythmias in telemetric long-term ECG in cynomolgus monkeys. *J Pharmacol Toxicol Methods* **124**, 107471 (2023).

12. Liu YW*, et al.* Human embryonic stem cell-derived cardiomyocytes restore function in infarcted hearts of non-human primates. *Nat Biotechnol* **36**, 597-605 (2018).

13. Moussavi A*, et al.* Comparison of cine and real-time cardiac MRI in rhesus macaques. *Sci Rep* **11**, 10713 (2021).

14. Heiberg E, Sjogren J, Ugander M, Carlsson M, Engblom H, Arheden H. Design and validation of Segment--freely available software for cardiovascular image analysis. *BMC Med Imaging* **10**, 1 (2010).

15. Wang X*, et al.* High-resolution myocardial T(1) mapping using single-shot inversion recovery fast low-angle shot MRI with radial undersampling and iterative reconstruction. *Br J Radiol* **89**, 20160255 (2016).

16. Kamp JC*, et al.* Comparative Analysis of Gene Expression in Fibroblastic Foci in Patients with Idiopathic Pulmonary Fibrosis and Pulmonary Sarcoidosis. *Cells* **11**, (2022).

17. Nguyen NT, Lindsey ML, Jin YF. Systems analysis of gene ontology and biological pathways involved in post-myocardial infarction responses. *BMC Genomics* **16 Suppl 7**, S18 (2015).

18. Abercrombie M. Estimation of nuclear population from microtome sections. *Anat Rec* **94**, 239-247 (1946).

19. Cerqueira MD*, et al.* Standardized myocardial segmentation and nomenclature for tomographic imaging of the heart. A statement for healthcare professionals from the Cardiac Imaging Committee of the Council on Clinical Cardiology of the American Heart Association. *Circulation* **105**, 539-542 (2002).

20. Boergens KM*, et al.* webKnossos: efficient online 3D data annotation for connectomics. *Nat Methods* **14**, 691-694 (2017).

21. Wiegerinck RF*, et al.* Force frequency relationship of the human ventricle increases during early postnatal development. *Pediatr Res* **65**, 414-419 (2009).

22. Chen TH*, et al.* Cardiac fibrosis in mouse expressing DsRed tetramers involves chronic autophagy and proteasome degradation insufficiency. *Oncotarget* **7**, 54274-54289 (2016).
