# Supplementary material for: Human iPSC-cardiomyocyte-aggregate cell therapy in non-human primates and correlation of heart recovery with contractile and electrophysiological cardiomyocyte properties
Source: Nat Commun. 2026 Aug 26;17:9066. doi: 10.1038/s41467-026-77103-0 (PMC13518835; doi:10.1038/s41467-026-77103-0)
Supplement: Supplementary file 2 [file 41467_2026_77103_MOESM2_ESM.pdf]

Description of Additional Supplementary Files

File name: Supplementary Video 1
Description: hiCMAs derived from the reporter iPSC line "*Amber*"
Light microscopy video representative of contractile CM-aggregates, sampled and assessed at check point 2 (CP2). Note the well-defined smooth outer appearance of each aggregate and the lack of single cells. Scale bar (lower right corner) represents 200 µm.

File name: Supplementary Video 2
Description: hiCMAs derived from the reporter iPSC line "*Ruby*"
Fluorescence microscopy video representative of contractile CM-aggregates, sampled and assessed at CP2. The expected flashing of individual aggregates over time is evoked by calcium transients visualised by the reporter line specific transgenic calcium sensor GCaMP6f. Scale bar (lower right corner) represents 200 µm.
