# Supplementary material for: Human iPSC-cardiomyocyte-aggregate cell therapy in non-human primates and correlation of heart recovery with contractile and electrophysiological cardiomyocyte properties
Source: Nat Commun. 2026 Aug 26;17:9066. doi: 10.1038/s41467-026-77103-0 (PMC13518835; doi:10.1038/s41467-026-77103-0)
Supplement: Supplementary file 3 [file 41467_2026_77103_MOESM5_ESM.pdf]

nature portfolio

Corresponding author(s): Prof. Dr. Ulrich Martin, Dr. Robert Zweigerdt

Last updated by author(s): May 28, 2026

Reporting Summary

Nature Portfolio wishes to improve the reproducibility of the work that we publish. This form provides structure for consistency and transparency in reporting. For further information on Nature Portfolio policies, see our Editorial Policies and the Editorial Policy Checklist.

Statistics

For all statistical analyses, confirm that the following items are present in the figure legend, table legend, main text, or Methods section.

n/a	Confirmed	
☐	☒	The exact sample size (*n*) for each experimental group/condition, given as a discrete number and unit of measurement
☐	☒	A statement on whether measurements were taken from distinct samples or whether the same sample was measured repeatedly
☐	☒	The statistical test(s) used AND whether they are one- or two-sided<br>*Only common tests should be described solely by name; describe more complex techniques in the Methods section.*
☒	☐	A description of all covariates tested
☐	☒	A description of any assumptions or corrections, such as tests of normality and adjustment for multiple comparisons
☐	☒	A full description of the statistical parameters including central tendency (e.g. means) or other basic estimates (e.g. regression coefficient) AND variation (e.g. standard deviation) or associated estimates of uncertainty (e.g. confidence intervals)
☒	☐	For null hypothesis testing, the test statistic (e.g. *F*, *t*, *r*) with confidence intervals, effect sizes, degrees of freedom and *P* value noted<br>*Give P values as exact values whenever suitable.*
☒	☐	For Bayesian analysis, information on the choice of priors and Markov chain Monte Carlo settings
☒	☐	For hierarchical and complex designs, identification of the appropriate level for tests and full reporting of outcomes
☐	☒	Estimates of effect sizes (e.g. Cohen's *d*, Pearson's *r*), indicating how they were calculated

Our web collection on statistics for biologists contains articles on many of the points above.

Software and code

Policy information about availability of computer code

Data collection	Ponemah version 6.20 (Data Sciences International (DSI), St. Paul, MN, USA)<br>ZEN version 3.3 or AxioVision SE64 Rel. 4.9 software (Zeiss, Jena, Germany)<br>PCLAMP software and Digidata 1550 (Molecular Devices)<br>MACSQuantify Software 3.0 (Miltenyi Biotec, Bergisch-Gladbach, Germany)<br>Custom-made bioreactor system and analysis software (Central Research Workshop, Hannover Medical School)<br>CX50 software version OS-12.676 (Phillips N.V. Amsterdam, Netherlands)<br>NovaSeq X Sequencing System Software Suite v1.1.0 (Illumina, San Diego, CA, USA)
Data analysis	Ponemah version 6.20 (Data Sciences International (DSI), St. Paul, MN, USA)<br>Segment (Version 2.0 R6435, Medviso, Lund, Sweden)<br>JPCCalc software (Dr. P. Barry, University of South Wales, Sydney, Austraia)<br>ImageJ version 1.53c (https://imagej.nih.gov/ij/)<br>webKnossos (Scalable minds UG, Potsdam, Germany)<br>Browser-based viewer (Histomography, Germany)<br>FlowJo software (Flowjo, LLC)<br>Custom-made bioreactor system and analysis software (Central Research Workshop, Hannover Medical School)<br>FastQC; Trimmomatic v.0.36; STAR aligner v.2.5.2b; Subread package v.1.5.2; GeneSCF v1.1-p2<br>R version 4.5.2 (The R Foundation for Statistical Computing)<br>GraphPad Prism Version 9 or 10 (GraphPad Software Inc., La Jolla, CA, USA)

For manuscripts utilizing custom algorithms or software that are central to the research but not yet described in published literature, software must be made available to editors and reviewers. We strongly encourage code deposition in a community repository (e.g. GitHub). See the Nature Portfolio guidelines for submitting code & software for further information.

Data

Policy information about availability of data

All manuscripts must include a data availability statement. This statement should provide the following information, where applicable:

- Accession codes, unique identifiers, or web links for publicly available datasets
- A description of any restrictions on data availability
- For clinical datasets or third party data, please ensure that the statement adheres to our policy

The authors declare that all data supporting the findings of this study are available within the article and its Supplementary Information files or in the Source Data file. RNASeq data have been deposited in the NCBI Sequence Read Archive (SRA) under the BioProject ID PRJNA1248569.

Research involving human participants, their data, or biological material

Policy information about studies with human participants or human data. See also policy information about sex, gender (identity/presentation), and sexual orientation and race, ethnicity and racism.

Reporting on sex and gender	*Use the terms sex (biological attribute) and gender (shaped by social and cultural circumstances) carefully in order to avoid confusing both terms. Indicate if findings apply to only one sex or gender; describe whether sex and gender were considered in study design; whether sex and/or gender was determined based on self-reporting or assigned and methods used. Provide in the source data disaggregated sex and gender data, where this information has been collected, and if consent has been obtained for sharing of individual-level data; provide overall numbers in this Reporting Summary. Please state if this information has not been collected. Report sex- and gender-based analyses where performed, justify reasons for lack of sex- and gender-based analysis.*
Reporting on race, ethnicity, or other socially relevant groupings	*Please specify the socially constructed or socially relevant categorization variable(s) used in your manuscript and explain why they were used. Please note that such variables should not be used as proxies for other socially constructed/relevant variables (for example, race or ethnicity should not be used as a proxy for socioeconomic status). Provide clear definitions of the relevant terms used, how they were provided (by the participants/respondents, the researchers, or third parties), and the method(s) used to classify people into the different categories (e.g. self-report, census or administrative data, social media data, etc.) Please provide details about how you controlled for confounding variables in your analyses.*
Population characteristics	*Describe the covariate-relevant population characteristics of the human research participants (e.g. age, genotypic information, past and current diagnosis and treatment categories). If you filled out the behavioural & social sciences study design questions and have nothing to add here, write "See above."*
Recruitment	*Describe how participants were recruited. Outline any potential self-selection bias or other biases that may be present and how these are likely to impact results.*
Ethics oversight	*Identify the organization(s) that approved the study protocol.*

Note that full information on the approval of the study protocol must also be provided in the manuscript.

Field-specific reporting

Please select the one below that is the best fit for your research. If you are not sure, read the appropriate sections before making your selection.

☒ Life sciences ☐ Behavioural & social sciences ☐ Ecological, evolutionary & environmental sciences

For a reference copy of the document with all sections, see nature.com/documents/nr-reporting-summary-flat.pdf

Life sciences study design

All studies must disclose on these points even when the disclosure is negative.

Sample size	Sample sizes for non-human primate experiments were initially chosen based on biometric planning for the primary end-point of the study (long-term improvement of heart function, i.e. LVEF). Based on the expected variation for MRI assessment of LVEF in cynomolgus monkeys (Sampath et al. PLOS One 2015), we determined n=5 per group to be sufficient to detect a difference of LVEF of 13.38 %p (p=0.05; power=80%; GraphPad StatMate 2.00), which was within the limits of previously reported functional improvement of EF in a similar NHP-model (Shiba et al., Nature 2016; assessed by µCT).
Data exclusions	As described in the manuscript and detailed in the animal study design (Fig. S4B), 2 animals were excluded from the final MRI analysis of the effect of hiCMA transplantation because they were found to have no confirmed MI (i.e. difference in LVEF post MI induction < 5%p).
Replication	All attemps at replication of the research findings were successful unless stated otherwise. Failure to replicate infarct-related impairment of heart function was reported for two animals; any analytical limitations due to the low number of animals were discussed in detail.
Randomization	Animals were allocated randomly to the experimental groups. For the primary end-point of the study (long-term improvement of heart

Randomization	function, i.e. LVEF), we excluded the influence of potential covariates (sex, age, weight) by including only male animals of similar age and weight (see table 1; no significant difference between groups with One-way ANOVA and Bonferroni's multiple comparison test).
Blinding	For the primary end-point of the study (long-term improvement of heart function, i.e. LVEF), two independent observers performed data analysis in a blinded fashion, with good inter-observer agreement (depicted in Fig. S3D).

Reporting for specific materials, systems and methods

We require information from authors about some types of materials, experimental systems and methods used in many studies. Here, indicate whether each material, system or method listed is relevant to your study. If you are not sure if a list item applies to your research, read the appropriate section before selecting a response.

Materials & experimental systems

n/a	Involved in the study
☐	☒ Antibodies
☐	☒ Eukaryotic cell lines
☒	☐ Palaeontology and archaeology
☐	☒ Animals and other organisms
☒	☐ Clinical data
☒	☐ Dual use research of concern
☒	☐ Plants

Methods

n/a	Involved in the study
☒	☐ ChIP-seq
☐	☒ Flow cytometry
☒	☐ MRI-based neuroimaging

Antibodies

Antibodies used

“Name” “Catalog Nr.” “Company” “Dilution used” “Application”
ACTN2 (SA) #A7811, Sigma-Aldrich, St. Louis, US; 1:800; Flow cytometry
CD31 #M0823 Dako, Agilent Santa Clara, US; 1:20; Immunofluorescence
cTnI #ab52862 Abcam, Cambridge, UK; 1:500; Immunofluorescence
cTnT #MA5-12960 Thermo Fisher Scientific, Waltham, US; 1:200; Flow cytometry
CX43 #C8093 Sigma-Aldrich, St. Louis, US; 1:1000; Immunofluorescence
GATA4 #ab124265 Abcam, Cambridge, UK; 1:1000; Immunofluorescence
GFP # R1091P Acris, OriGene, Rockville, US; 1:500; Immunofluorescence
Ki67 #M7240 Dako, Agilent, Rockville, US; 1:100; Immunofluorescence
mCherry #ab167453 Abcam, Cambridge, UK; 1:500; Immunofluorescence
MYH (MF20) #AB2147781, Hybrodoma Bank; 1:20; Flow cytometry
MLC2a #311011 Synaptic Systems, Göttingen, DE; 1:100; Immunofluorescence
MLC2v #ab79935 Abcam, Cambridge, UK; 1:100; Immunofluorescence
TTN (9 D10) #AB528491 Hybridoma Bank, University of Iowa, US; 1:200 / 1:20; Immunofluorescence
TNNT #MS-295-P Thermo Fisher Scientific, Waltham, US; 1:100; Immunofluorescence
TNNT #15513-1-AP Proteintech, Manchester, UK; 1:500; Immunofluorescence
Negative Control #X0942 mouse IgM Dako, Agilent, Santa Clara, US; Immunofluorescence
Negative Control #ab37415 rabbit IgG Abcam, Cambridge, UK; Immunofluorescence
Negative Control #X0943 mouse IgG2a mouse IgG2b Dako, Agilent, Santa Clara, US; Immunofluorescence
Negative Control #X0944 mouse IgG2b Dako, Agilent, Santa Clara, US; Immunofluorescence
Negative Control #X0931 mouse IgG1 Dako, Agilent, Santa Clara, US; Immunofluorescence
Negative Control #ab37373 Goat IgG Abcam, Cambridge, UK; Immunofluorescence
Donkey anti-mouse IgG #ab175658 Alexa Fluor 405 Abcam, Cambridge, UK; 1:2000; Immunofluorescence
Donkey anti-mouse IgG #715-605-151 Alexa Fluor 647 Dianova, Hamburg, DE; 1:300; Immunofluorescence;
Donkey anti-mouse IgG #715-165-150 Cy3 Dianova, Hamburg, DE; 1:300; Flow cytometry, Immunofluorescence,
Donkey anti-mouse IgG #715-545-151 Alexa Fluor 488 Dianova, Hamburg, DE; 1:300; Immunofluorescence
Donkey anti-mouse IgM #715-605-140 Alexa Fluor 647 Dianova, Hamburg, DE; 1:300; Flow cytometry, Immunofluorescence
Donkey anti-mouse IgM #715-485-020 DyLight 488 Dianova, Hamburg, DE; 1:200; Immunofluorescence
Donkey anti-rabbit IgG #ab175651 Alexa Fluor 405 Abcam, Cambridge, UK; 1:2000; Immunofluorescence
Donkey anti-rabbit IgG #711-505-152 DyLight 549 Dianova, Hamburg, DE; 1:300; Immunofluorescence
Donkey anti-rabbit IgG #711-605-152 Alexa Fluor 647 Dianova, Hamburg, DE; 1:300; Immunofluorescence
Donkey anti-rabbit IgG #711-545-152 Alexa Fluor 488 Dianova, Hamburg, DE; 1:300; Immunofluorescence
Donkey anti-goat IgG #705-495-147 DyLight 649 Dianova, Hamburg, DE; 1:300; Immunofluorescence

Validation

All antibodies used in the study were commercially available, validated and applicable for use with human samples, as stated by the manufacturer.

Eukaryotic cell lines

Policy information about cell lines and Sex and Gender in Research

Cell line source(s)

All iPSC cell lines analysed in this study ("Ruby" MHHi001_A-5 and "Amber" MHHi001_A-11 clones) are based on HSC_Iso4_ADCF_SeV-iPS2 "Phönix", MHHi001-A generated from a male human cord blood donation (Haase et al.

	2017_StemCell Res)
Authentication	Source cells were fully characterized and published (Haase et al. 2017_StemCell Res (Phoenix); Haase et al. 2021_ StemCell Res (Ruby) and authenticated by STR analysis. iPSC lines were registered in the Human pluripotent stem cell registry hPSCreg https://hpscreg.eu/cell-line/MHHi001-A; https://hpscreg.eu/cell-line/MHHi001-A-5; https://hpscreg.eu/cell-line/MHHi001-A-11
Mycoplasma contamination	All generated cell lines were negatively tested for mycoplasma contamination (see publications above)
Commonly misidentified lines (See ICLAC register)	Nothing to declare

Animals and other research organisms

Policy information about studies involving animals; ARRIVE guidelines recommended for reporting animal research, and Sex and Gender in Research

Laboratory animals	Cynomolgus monkeys (Macaca fascicularis), age: 3.7 - 8.6 years
Wild animals	The study did not involve wild animals.
Reporting on sex	The study included 1 female (6.8 kg) and 14 male (7.3 - 12.8 kg) animals. No sex-based analysis was performed.
Field-collected samples	The study did not involve field collected samples.
Ethics oversight	The animal work was performed at the German Primate Center under conditions according to §§7-9 of the German Animal Welfare Act, adhering to the European Union guidelines (EU directive 2010/63/EU) on the use of non-human primates for biomedical research. The experiments were approved by the Lower Saxony State Office for Consumer Protection and Food Safety.

Note that full information on the approval of the study protocol must also be provided in the manuscript.

Plants

Seed stocks	*Report on the source of all seed stocks or other plant material used. If applicable, state the seed stock centre and catalogue number. If plant specimens were collected from the field, describe the collection location, date and sampling procedures.*
Novel plant genotypes	*Describe the methods by which all novel plant genotypes were produced. This includes those generated by transgenic approaches, gene editing, chemical/radiation-based mutagenesis and hybridization. For transgenic lines, describe the transformation method, the number of independent lines analyzed and the generation upon which experiments were performed. For gene-edited lines, describe the editor used, the endogenous sequence targeted for editing, the targeting guide RNA sequence (if applicable) and how the editor was applied.*
Authentication	*Describe any authentication procedures for each seed stock used or novel genotype generated. Describe any experiments used to assess the effect of a mutation and, where applicable, how potential secondary effects (e.g. second site T-DNA insertions, mosiacism, off-target gene editing) were examined.*

Flow Cytometry

Plots

Confirm that:

- [ ] The axis labels state the marker and fluorochrome used (e.g. CD4-FITC).
- [ ] The axis scales are clearly visible. Include numbers along axes only for bottom left plot of group (a 'group' is an analysis of identical markers).
- [ ] All plots are contour plots with outliers or pseudocolor plots.
- [ ] A numerical value for number of cells or percentage (with statistics) is provided.

Methodology

Sample preparation	Samples were prepared as described in the Supplemental information.
Instrument	MACSQuant Analyzer 10 (Miltenyi Biotec, cat. no. 130-096-343)
Software	FlowJo_v10 (Flowjo, LLC)
Cell population abundance	30,000 events were measured for evaluation of the relevant cell populations. Differentiated hiPSC-CMs used in this study showed a high purity of >90% for assessed standard cardiac markers (SA, cTnT, and MF20).

Gating strategy	Flow cytometry data were analyzed employing a sequential gating strategy. First, the primary cell population was identified based on forward scatter (FSC) and side scatter (SSC) parameters to include the principal cell population based on their size and granularity, while excluding debris and cellular aggregates. Doublets were eliminated utilizing FSC-A versus FSC-H plots. Subsequently, fluorescence-based gates were implemented to delineate marker expression. Boundaries distinguishing negative and positive populations were established through the use of unstained and isotype control samples. The negative population was defined according to the fluorescence distribution of unstained cells, while isotype controls were employed to account for non-specific binding. Positive populations were subsequently gated as cells exhibiting fluorescence intensity exceeding these control-defined thresholds.

☐ Tick this box to confirm that a figure exemplifying the gating strategy is provided in the Supplementary Information.
