# Supplementary material for: Human iPSC-cardiomyocyte-aggregate cell therapy in non-human primates and correlation of heart recovery with contractile and electrophysiological cardiomyocyte properties
Source: Nat Commun. 2026 Aug 26;17:9066. doi: 10.1038/s41467-026-77103-0 (PMC13518835; doi:10.1038/s41467-026-77103-0)
Supplement: Supplementary file 4 [file 41467_2026_77103_MOESM6_ESM.pdf]

nature portfolio

Peer Review File

Human iPSC-cardiomyocyte-aggregate cell therapy in non-human primates and correlation of heart recovery with contractile and electrophysiological cardiomyocyte properties

Corresponding Author: Professor Ulrich Martin

This file contains all reviewer reports in order by version, followed by all author rebuttals in order by version.

Version 0:

Reviewer comments:

Reviewer #1

(Remarks to the Author)
In this manuscript, Gruh and co-workers report the successful engraftment of hiPSC-derived cardiomyocyte aggregates (hiCMAs) in a non-human primate MI model. This study is from a leading group that has made major past contributions to the field, particularly toward the large-scale manufacturing of hiPSCs and hiPSC-derived cardiomyocytes. Their manuscript has several strengths including its use of well-validated cell manufacturing processes, complementary in vitro and in vivo approaches, and a very challenging, resource-intensive, but translationally relevant large-animal model. That said, this reviewer has several major concerns:

1) The novelty of the present study is somewhat undermined by prior work from the Fukuda group, who have also suggested that better graft outcomes can be obtained by the transplantation of aggregates rather than single-cell suspensions of hiPSC-derived cardiomyocytes. It was therefore surprising not to see the latter group's 2024 publication in the journal Circulation (PMID 38666382) cited, as it also describes the transplantation of cardiomyocyte aggregates in infarcted cynomolgus monkeys with functional endpoints out to 12-weeks post-transplantation. In this reviewer's opinion, while there would be significant value in replicating the Fukuda group's overall findings, the present authors should present their study in context relative to that earlier work, explaining any differences in methods or interpretations.

2) Statistical methods are vague for the data depicted in Fig. 1B&C. We are told there was no change in aggregate diameter between checkpoints, but there appears to be a trend toward slightly larger aggregates over time. There are definitely more frequent outliers at checkpoint 2 (with aggregate diameters exceeding 500 um), so one wonders why these didn't clog the 26G injection needles (inner diameter = 260 um). Was there a pre-filtration step?

3) Figure 2 also raises several questions about methods and data interpretation. First and most importantly, it seems premature to draw conclusions based on observations from engineered heart tissues from only two iPSC clones. The authors want to position this as a potency assay that may predict in vivo outcomes, but there is relatively high variability between technical replicates, and we have no sense whatsoever of clone-to-clone variability. I recognize it would be very difficult to enroll additional animals at this point, but in vitro experiments should incorporate a wider range of cell lines to be meaningful here. How does the parental line behave? Might there be other clones of the Ruby cell line, either from the same or a different transfection? Given the apparent off-target effects of co-expression of RedStar and GCaMP6f, it would be informative to see in vitro data from cell lines expressing each fluorescent protein separately.

4) We are told that the engineered heart tissues were paced but not at what rate. Force measurements should be rate-matched, but the traces in panels 2D & 2G appear to have been acquired be at different rates. A full force-frequency relationship would be even better.

5) Why didn't the authors normalize force measurements in engineered heart tissues by cross-sectional area? Is it possible that Ruby cardiomyocytes just had somewhat lower viability and thus formed sparser or less healthy cardiac tissues?

6) There appears to be a surprising amount of batch-to-batch heterogeneity in the authors' cell populations. Do they have data regarding the breakdown of cardiac subtypes (e.g., MLC2v percentages, atrial vs ventricular markers, etc) by batch or for Amber vs Ruby cardiomyocytes? Much of their graft RNAseq data could be explained if Ruby iPSC-derived cardiomyocytes were more atrial-like.

7) The possibility that clone-to-clone variation significantly affects transplantation outcomes is an intriguing concept, but again it is hard to draw firm conclusions from a transplantation study that involved only two iPSC clones and very small animal numbers. Data in Fig. 6A&B are not entirely convincing on this point. First, there appear to be differences between post-MI/pre-transplant LVEF between experimental groups, which would be confounding if true. Second, Amber hiCMA data plotted in 6B reflect only two(!) data-points and those had high variability. In this reviewer's opinion, the author's claim of "iPSC clone-dependent heart recovery" (even made in the manuscript title) should be toned down a bit.

8) The authors state that "No apparent differences were observed for graft integration and marker expression of CMs derived from the two reporter cell lines" (page 11, line 253). This statement should be supported by representative images from both experimental groups stained with the same set of markers as well as quantitative data (histomorphometry). The authors' graft RNAseq data suggest that there should be some differences.

9) Gruh et al also claim that hiCMAs formed grafts "comparable" to those obtained historically with the delivery of 10-20-fold more iPSC-derived cardiomyocytes in single-cell suspensions. However, quantitative data are lacking. The manuscript does include one example of a respectably sized graft (Fig. 5B), but the authors make no attempt to actually measure graft size by histomorphometry. (By contrast, the aforementioned study from the Fukuda group reports graft size as a percentage of LV and infarct area.) It would be helpful to include quantitative measurements of graft area for each cell recipient. This would be ideally broken down by zone since nearly all provided images show graft tissue implanted in host myocardium, not scar tissue.

10) Similarly, one also wonders whether the apparently different functional outcomes between Amber vs Ruby hiCMAs may simply reflect differences in graft size. It seems entirely plausible that Ruby cardiomyocytes had lower viability and just formed smaller grafts (and perhaps less healthy cardiac tissues in Fig. 2), leading to less contractility recovery and fewer arrhythmias.

11) Why does the duration of ECG monitoring vary so much by animal in Figures 7B-D? If I understand the experimental design correctly, each animal should have been monitored continuously to either 2 weeks or 3 months post-transplantation.

12) Do the authors have any speculation as to why the onset of arrhythmias was later in their study than in previous reports? This reviewer wonders whether that simply reflects smaller graft sizes, but there may also be differences in electrotonic source-sink with aggregates versus single cells.

Specific comments:
1) In Fig. 1B, why were aggregate sizes omitted for other hiCMA batches that were apparently transplanted (#01, #02, #04)?

2) For multiple figures (for example, Fig. 1C-H), it would be better to show representative data for both the Ruby and Amber conditions. If space is limited, one set could be included in the supplement. Moreover, in a number of instances in Figs. 1, 4, and 5, it is not stated which iPSC line was used to generate the presented data.

3) Sarcomere alignment is not apparent in Fig. 2B.

4) Maybe this is just from PDF compression, but there appear to be breaks in the force trace in panel 2G.

5) Scale bars are missing in panels 2E and 2H.

6) Cardiac MR images depicted in Fig. 3B are relatively low quality, and it is not at all evident what is even being pointed to in the post-contract images. Perhaps the poor contrast in the latter is because this was a permanent infarct model? Do the authors have short axis views pre- vs post-contrast?

7) The legend for Fig. 3B indicate that the infarct examples were at 14-days post-MI, but the Supplemental Methods state that these were 3-month post-MI.

8) It would be clearer to refer to the "sham" experimental group as "vehicle" recipients. These animals underwent MI but not hiCMA transplantation, while sham usually no procedure whatsoever.

9) It would be interesting to know whether serum TnT (or other biomarkers) correlated with infarct size by either MRI or histology.

10) It would be interesting to have the authors' perspectives about how TnT levels might be used in this animal model in the future. For example, could early TnT measurements serve as inclusion-exclusion criteria (which were badly needed in the present study)?

11) What is meant by "in principle GMP-compliant conditions" (page 16, line 269)?

12) Outcomes are reported by animal in Table 1, but it would be more transparent to plot the relevant raw data, i.e. “raw” LVEF values not just normalized LVEF as currently done in Figs. 6 and S3.

13) Statistical methods should be clarified for each figure; in some cases (e.g., Fig. 1L), it is not stated whether or what statistical comparisons were performed.

Reviewer #2

(Remarks to the Author)
The authors revealed the safety and efficacy of the transplantation of human iPSC cardiomyocyte aggregates into the hearts of cynomolgus monkeys with myocardial infarction. They also showed that its effectiveness correlates with the iPSC clone-dependent contractile function of cardiomyocytes. These findings will contribute to the future development of cell transplantation therapy using human iPSC-derived cardiomyocytes. However, in some experiments, the presented data is insufficient. The authors need to adequately address the following concerns:

Major
1. In Figure 1, hiCMAs from CP1 and hiCMAs from CP2 are compared. Regarding the immunostaining microscopic images in Figure 1E-H, the authors should present microscopic images of hiCMAs from CP1 and CP2 for each antibody. Additionally, do the authors argue that key cellular and physical properties of hiCMAs are maintained in CP2 after long-term culture, as stated in Lines 141-143? Culturing for a longer period would be disadvantageous from both a cost and time perspective. If the authors suggest that hiCMAs from CP2 are more mature than those from CP1, and that using more mature hiCMAs would be better for cell transplantation and BCT formation, it should be clearly stated.

2. In Figure 2, Amber-BCT and Ruby-BCT are compared. Immunostaining for SA is performed on Amber-BCT in Figure 2B and immunostaining for cTnT is performed on Ruby-BCT in Figure 2E. The authors should present microscopic images of Amber-BCT and Ruby-BCT at the same scale, where immunostaining was performed using the same antibody for comparison. Additionally, it would be meaningful to compare the profiles of human iPSC-derived cardiomyocytes contained in Amber-BCT and Ruby-BCT through immunostaining for MLC2a/MLC2v, etc.

3. In Figure 5, the microscopic images of immunostaining using each antibody are only presented for either the Amber-treated group or the Ruby-treated group. Considering the results showing that Amber-hiCMAs and Ruby-hiCMAs had different effects on the improvement of cardiac function, the authors should present and compare the microscopic images of the grafts from each group. For example, it should be demonstrated whether there are any differences in angiogenesis between the Amber-hiCMA grafts and the Ruby-hiCMA grafts.

4. The authors concluded that Amber-hiCMAs improved cardiac function, whereas Ruby-hiCMAs did not. They noted that in monkeys with large impairment induced by myocardial infarction, a slight recovery was observed even in the sham group after three months. In the Ruby-hiCMAs-treated group, the extent of cardiac function decline after myocardial infarction was less than that in the Amber-hiCMAs-treated group. Could this be related to the lack of improvement in cardiac function in the Ruby-hiCMAs-treated group?

5. The transplantation of Ruby-hiCMAs resulted in less improvement in cardiac function and a lower incidence of arrhythmia compared to the transplantation of Amber-hiCMAs. These results could be explained if the engraftment rate in the Ruby-hiCMAs-treated group was lower than that in the Amber-hiCMAs-treated group. Were there any differences in the engraftment rates between the two groups?

6. What might be the reason for the lower frequency of arrhythmias in the Ruby-hiCMAs-treated group?

7. The authors showed that Amber-BCTs exhibited stronger contractile function than Ruby-BCTs. RNA sequencing also revealed that gene expression related to cardiac muscle contraction and muscle filament sliding was upregulated in Amber-BCTs compared to Ruby-BCTs. These findings were suggested as the reasons why Amber-hiCMAs had a greater effect on improving cardiac function than Ruby-hiCMAs. In cell transplantation therapy using human iPSC-derived cardiomyocytes, both the direct contractile function of the transplanted cardiomyocytes and paracrine effects are considered to be the mechanisms for the improvement in cardiac function. Were there any differences in the paracrine effects between the two groups?

8. Related to the above, regarding the results of RNA sequencing, the authors should present not only the heatmap of specific genes shown in Figure 6CD but also the results of the GO analysis.

9. In Table 1, #05 and #10 are annotated as “lower absolute values for MRI parameters due to analysis with real-time mode”. #05 was excluded from the analysis in Figure 6, along with #14, due to very small LVEF changes after MI. Is #10 an appropriate subject to include in the analysis?

10. The ischemia/reperfusion model is more clinically relevant than the permanent ligation model. Why did the authors choose the permanent ligation model in the present study?

Minor
1. HF, which is an abbreviation of heart failure, is used only in Line 62 and not in other sections.
2. The notation for the Figure in the text is inconsistent, with some instances using Figure and others using Fig. Please make

it consistent.
3. In the hiCMA B2 Ruby group of Figure 6B, some graphs are missing error bars indicating standard deviation and should be corrected.

Version 1:

Reviewer comments:

Reviewer #1

(Remarks to the Author)
The revised manuscript by Gruh et al is substantially improved, and this reviewer greatly appreciates the thoughtful rebuttal and interesting new data provided by the authors. The additional phenotyping of Amber versus Ruby CMs and new in vitro data with BCTs formed with other iPSC lines are particularly helpful. Findings in the revised manuscript will be of significant interest to the field, but there are a few minor remaining issues that would further strengthen clarity and impact:

1. Legends should be provided for all heat maps of gene expression (Figs. 5K, 6C-D, S7B, S8E, and S9). It is currently unclear whether the color scale in each represents Z-score, log fold-change, or something else entirely.

2. While fully understanding the authors' rationale for normalizing LVEF to the pre-infarct LVEF (LV-rEF), this approach is a bit non-standard. It would be more transparent to separately plot both LV-rEF and "raw" LVEF values (with the latter at least included in a supplemental figure). This was recommended previously, but the authors have instead just shuffled around the ordering of the data in Table 1, leaving this work to the reader and making it harder to compare between transplantation studies.

3. In Figures 2E and K, the calcium transients across various pacing frequencies are squished, making it impossible to distinguish individual transients at higher frequencies. It would be helpful if the authors could adjust the time axis and horizontally expand or stack these traces for easier visibility.

4. Arguably, one of the more interesting findings of this manuscript is the unexpectedly negative consequences of nuclear RedStar fluorescent protein expression on CM phenotype. The authors may want to consider highlighting this important "lesson learned" by adding mention of the fluorescent protein reporter issue in the abstract (space permitting).

Reviewer #2

(Remarks to the Author)
The authors have generally responded appropriately to our concerns and have revised the manuscript accordingly. However, we have the following concerns regarding the authors' response to our Question 5.

1. The authors measured the volume and cell number of Ruby-hiCMA grafts and Amber-hiCMA grafts, and demonstrated that Ruby-hiCMA grafts are not smaller than Amber-hiCMA grafts. We are convinced that the lack of improvement in cardiac function and the absence of arrhythmias in the Ruby-hiCMA-treated group were not due to poor engraftment. However, the reported graft volumes—6–8 mm^3 for Amber-hiCMA graft, and 5.6 mm^3 and 17.2 mm^3 for Ruby-hiCMA grafts—appear quite small when considering the size of the cynomolgus monkey heart. One of the key points emphasized in this study is that contractile force of hiPSC-CMs differs among cell lines and correlates with the improvement in cardiac function after transplantation. Is it appropriate to attribute the difference in cardiac functional improvement between the Amber-hiCMA-treated and Ruby-hiCMA-treated groups solely to differences in the contractile force of these relatively small grafts?

2. In this study, 50 million cardiomyocytes were transplanted as aggregates, with approximately 1.8% engraftment, resulting in the graft volumes described above. In contrast, in the study by Liu et al., 750 million cardiomyocytes were transplanted as single cells, with approximately 12% engraftment and a graft volume of 370 mm^3. While it is true that this study achieved a comparable improvement in cardiac function with less than one-tenth the number of cells, the engraftment rate reported here is lower than that in the single-cell transplantation study by Liu et al. Does this appear to contradict the authors' suggestion that aggregate transplantation improves engraftment efficiency? We acknowledge that direct comparisons are challenging due to differences in culture conditions, transplantation methods, and analytical approaches across studies. In addition, the authors state "However, due to the limited number of animals that can be tested due to ethical, regulatory and financial restrictions, our as well as the Kobyashi study have not performed a direct comparison of the aggregate approach versus single cell administration." in the Discussion. Although Kobayashi et al. did not directly compare aggregate versus single-cell transplantation in primates, their mouse study (shown in the Supplementary Data) demonstrated that aggregate transplantation resulted in approximately threefold higher engraftment than single-cell transplantation. How do the authors interpret the difference in engraftment rates between aggregate and single-cell transplantation?

3. Please carefully check for typographical errors once again. For example:

L605 Connexin 43 (Cx43; encoded by GJC1) → should this be GJA1?

L525 Kobyashi → Kobayashi

Open Access This Peer Review File is licensed under a Creative Commons Attribution 4.0 International License, which permits use, sharing, adaptation, distribution and reproduction in any medium or format, as long as you give appropriate credit to the original author(s) and the source, provide a link to the Creative Commons license, and indicate if changes were made.
In cases where reviewers are anonymous, credit should be given to 'Anonymous Referee' and the source.
The images or other third party material in this Peer Review File are included in the article's Creative Commons license, unless indicated otherwise in a credit line to the material. If material is not included in the article's Creative Commons license and your intended use is not permitted by statutory regulation or exceeds the permitted use, you will need to obtain permission directly from the copyright holder.
To view a copy of this license, visit https://creativecommons.org/licenses/by/4.0/

Rebuttal letter NCOMMS-25-25976

We thank both reviewers for their valuable comments and for giving us the opportunity to submit a revised version of our manuscript. We have addressed all remarks point by point in the following text (blue font).

Reviewers' comments:

Reviewer #1:

In this manuscript, Gruh and co-workers report the successful engraftment of hiPSC-derived cardiomyocyte aggregates (hiCMAs) in a non-human primate MI model. This study is from a leading group that has made major past contributions to the field, particularly toward the large-scale manufacturing of hiPSCs and hiPSC-derived cardiomyocytes. Their manuscript has several strengths including its use of well-validated cell manufacturing processes, complementary in vitro and in vivo approaches, and a very challenging, resource-intensive, but translationally relevant large-animal model. That said, this reviewer has several major concerns:

1) The novelty of the present study is somewhat undermined by prior work from the Fukuda group, who have also suggested that better graft outcomes can be obtained by the transplantation of aggregates rather than single-cell suspensions of hiPSC-derived cardiomyocytes. It was therefore surprising not to see the latter group's 2024 publication in the journal Circulation (PMID 38666382) cited, as it also describes the transplantation of cardiomyocyte aggregates in infarcted cynomolgus monkeys with functional endpoints out to 12-weeks post-transplantation. In this reviewer's opinion, while there would be significant value in replicating the Fukuda group's overall findings, the present authors should present their study in context relative to that earlier work, explaining any differences in methods or interpretations.

We apologize for overlooking this very important recent paper. According to the reviewer's request the study is now contextualized in the introduction and discussion of our manuscript.

2) Statistical methods are vague for the data depicted in Fig. 1B&C. We are told there was no change in aggregate diameter between checkpoints, but there appears to be a trend toward slightly larger aggregates over time. There are definitely more frequent outliers at checkpoint 2 (with aggregate diameters exceeding 500 um), so one wonders why these didn't clog the 26G injection needles (inner diameter = 260 um). Was there a pre-filtration step?

We thank the reviewer for this relevant and detailed notion and have updated Figures 1B&C accordingly.
The aggregate diameter analysis of our Check Points 1/2 (CP1/ 2) samples is as exact as possible in frame of the available sample size and the applied method, which is an established approach that we have efficiently applied in numerous well-cited publications before [1, 2, 3, 4]. We have included details of the statistical methods in the figure legends.
Notably, for full transparency, we show in the **revised Fig. 1B** the diameter of all individual aggregates (represented by dots) assessed in our assay including the respective minor outliers. This in mind, we like to emphasize that – across all samples shown in figure 1B - there are not substantially more outliers present at CP2 compared to CP1. Only one sample

(indicated as #5) is somewhat more heterogeneous, but the absolute number of outliers (i. e. with aggregate diameters exceeding 500 um) is actually very limited within the overall sample size tested.

However, regarding the reviewer's question on the potential clogging of the applied 26G injection needle (inner diameter = 260 µm) due to the presence of larger size aggregates, we would like to note that this issue has been considered when initiating the study by performing the following experiment:

Aggregates of CP2 samples were pushed through the 26G injection needle and their appearance and viability were analyzed (via our recently published method in [4]) before and after transition through the needle. In brief: our observations suggest the aggregates are not rigid but have rather flexible morphological properties, i. e. they are slightly longitudinally deformed when pushed through the injection needle. Consequently, the procedure is not disrupting aggregate integrity and cell viability is not compromised. The longitudinal shape (resulting from the passage through the needle) is transient, i. e. the aggregates are turning back to their original round morphology shortly after the passage. These results (shown in **new Fig. S1O**) and are in agreement with the notion, that the surgeons injecting the aggregates suspension into the myocardium, did not observe substantial clogging issues throughout the study.

Finally, we like to note that no pre-filtration step of the applied CP2 aggregates was performed; this is apparently not required (vis-à-vis the above discussion) and notably simplifies process steps, logistics and regulatory compliance alongside the envisioned clinical translation.

3) Figure 2 also raises several questions about methods and data interpretation. First and most importantly, it seems premature to draw conclusions based on observations from engineered heart tissues from only two iPSC clones. The authors want to position this as a potency assay that may predict in vivo outcomes, but there is relatively high variability between technical replicates, and we have no sense whatsoever of clone-to-clone variability. I recognize it would be very difficult to enroll additional animals at this point, but in vitro experiments should incorporate a wider range of cell lines to be meaningful here. How does the parental line behave? Might there be other clones of the Ruby cell line, either from the same or a different transfection? Given the apparent off-target effects of co-expression of RedStar and GCaMP6f, it would be informative to see in vitro data from cell lines expressing each fluorescent protein separately.

In response to these important questions and suggestions, we have performed comprehensive additional comparative analyses of BCTs generated from CMs that were derived from seven additional iPSC clones/ lines:

First, we analyzed two additional, biologically independent clones generated in parallel to (and thus representing equivalent properties of) both Amber and Ruby, respectively (thus 4 clones in total). These investigations revealed highly similar results with significantly higher forces for all 'Amber'- vs. all 'Ruby' clones-derived BCTs (see **new Figures S2C & D**).

Second, we compared our force measurement results with three additional iPSC lines that is: i) Phoenix (non-transgenic maternal iPSC line of Amber and Ruby), ii) an only GCaMP6f transgene expressing line and iii) an only nuclear RedStar (a variant of the red fluorescent protein DsRed) transgene expressing line.

Confirming our initial data, these analyses revealed relatively low active forces in BCTs generated from the 2 additional Ruby clones (expressing both GCaMP6f and nuclear RedStar) and the new cell line expressing just nuclear RedStar. On the other hand, BCTs generated

from: the two additional Amber clones (expressing nuclear Venus; a GFP variant), Phoenix, and the GCaMP6f only reporter line, all showed significantly higher forces compared to the nuclear RedStar expressing Ruby clones and the RedStar only line (**see new Fig S2E**).

Together, these comprehensive analyses strongly suggest that the observed functional differences between Amber and Ruby are, unexpectedly, caused by the expression of the nuclear RedStar, rather than resulting from clonal genetic or epigenetic aberrations introduced as "off-target effects" of the applied TALEN machinery or introduced during iPSC single cell cloning. The expression of nuclear RedStar in iPSC-derived CMs apparently leads to a dysregulated expression of various cardiac genes resulting into impaired contractile function (**revised Fig. S2E & K**) and modulated electrophysiological properties (see **new Figure S8**), and of secreted factors associated with post myocardial infarction response (**new Fig. S7B**).

Interestingly, Chen et al. showed distinct gene expression changes in a mouse model of cardiac RFP-overexpression leading to fibrosis, dysregulated protein degradation, and consequently serious functional impairment of the heart [5]. Notably, we found some related gene expression changes in the 'Ruby' graft in animal #10 (see **new Fig. S9**), suggesting a similar mechanism of RFP aggregation-mediated cardiac toxicity resulting from the nuclear RedStar expression in 'Ruby'-CMs/ -hiCMAs/ -grafts.

4) We are told that the engineered heart tissues were paced but not at what rate. Force measurements should be rate-matched, but the traces in panels 2D & 2G appear to have been acquired be at different rates. A full force-frequency relationship would be even better.

According to the reviewer's request we have now replaced the former graphs in Figure 2 D&G by graphs showing examples of original traces for spontaneous contractions and after pacing at 1, 2, and 3 Hz as well (now **revised Fig. 2E & 2K**); a full analysis of the force-frequency relationships for both cell lines *Amber* and *Ruby* was added to the supplemental material (see **new Fig. S2A & B**).

5) Why didn't the authors normalize force measurements in engineered heart tissues by cross-sectional area? Is it possible that Ruby cardiomyocytes just had somewhat lower viability and thus formed sparser or less healthy cardiac tissues?

In reply to this request, we have now amended and replaced the former graphs in Figure 2 K & L by graphs showing force measurements normalized by cross-sectional area (now **revised Figure 2 L&M**). We have performed this normalization for all other BCT data throughout the revised manuscript.

6) There appears to be a surprising amount of batch-to-batch heterogeneity in the authors' cell populations. Do they have data regarding the breakdown of cardiac subtypes (e.g., MLC2v percentages, atrial vs ventricular markers, etc.) by batch or for Amber vs Ruby cardiomyocytes? Much of their graft RNAseq data could be explained if Ruby iPSC-derived cardiomyocytes were more atrial-like.

The reviewer raises an insightful question regarding the breakdown of cardiomyocyte subtypes resulting from our cardiac differentiation/ production process.

The quantitative assessment of cardiac aggregate diameters at CP1/ CP2 as well as the flow cytometry analysis of dissociated cells for cTnT and SA at the protein level, actually indicated high batch-to-batch homogeneity irrespective of both reporter iPSC lines tested (see **revised**

Fig. 1B, C, D and our reply to the reviewer's related question #2) above); no apparent differences were detected by IF staining of aggregate sections as well (**revised Fig. 1 E-J and new Fig. S1**).

However, inspired by this reviewer, we have now conducted patch clamp analyses of individual CMs derived from dissociated aggregates (**new Fig. S1G-N**). This revealed a highly enriched ventricular-like immature CM phenotype for derivatives from both cell lines Amber and Ruby; confirming our previous differentiation results on numerous independent iPSC and ESC lines [1, 4, 6]. Although the presence of a minor proportion of atrial-like CM phenotypes is not excluded, the patch clamp data argue against a substantially different cellular composition of Amber vs. Ruby CM populations as a mechanism underlying the observed differences in force development in the BCT assay.

The patch clamp data rather suggests that Ruby-CMs display substantially reduced sodium currents, significantly reduced upstroke velocity and a significantly reduced Action Potential (AP) amplitude compared to Amber-CMs (**new Fig. S8A-D**); these phenotypic data are in agreement with the differential gene expression analysis revealing reduced expression of key sodium current channels in Ruby- versus Amber-derived BCTs (**new Fig. S8E**; see also reply to question #6 or reviewer #2). Together with the difference in sarcomere gene expression patterns (Fig. 6C, D), our new datasets reveal a rational explanation for the differential functional performance of respective CM populations generated in our study (**new Fig. S8F, G**).

7) The possibility that clone-to-clone variation significantly affects transplantation outcomes is an intriguing concept, but again it is hard to draw firm conclusions from a transplantation study that involved only two iPSC clones and very small animal numbers. Data in Fig. 6A&B are not entirely convincing on this point. First, there appear to be differences between post-MI/pre-transplant LVEF between experimental groups, which would be confounding if true. Second, Amber hiCMA data plotted in 6B reflect only two(!) data-points and those had high variability. In this reviewer's opinion, the author's claim of "iPSC clone-dependent heart recovery" (even made in the manuscript title) should be toned down a bit.

In full agreement with reviewer's notion (and as already discussed in our first manuscript draft) the relatively small numbers of animals typically available for NHP studies lead to additional challenges (beyond the general problems in animal experimentation) for meaningful functional analyses of heart recovery after experimentally induced MI.

Already for pre-MI LVEF values we observed some variation, most likely due to inter-individual differences in animal age, weight etc. However, the extremely low pre-MI values for animals #05 and #10 (**revised Table 1**) can be explained by technical reasons: these two animals were evaluated using the Real-Time approach due to distorted ECG signals, while standard CINE MRI was used for assessing all other animals.

While induction of MI was successful in most animals with a loss of contractile myocardium in left ventricular and septal areas near the apex, there was indeed a substantial degree of variance in LVEF after MI (**revised Table 1**). These differences most likely reflect different sizes of ischemic and resulting infarcted areas due to inter-individual differences in coronary artery anatomy [7] and the positioning of the experimental coronary artery ligation.

To account for these limitations, we decided to determine heart function as LVEF relative to the pre-MI LVEF (LV-rEF), as this normalization increased the comparability of results in the relatively small group of animals.

However, despite expressing heart function as LV-rEF, the higher average LV-rEF after MI in the Ruby vs Amber animals is still most likely just reflecting the limitations of the low number of animals that could be included on our study for regulatory and practical reasons.
We agree with the reviewer that differences between post-MI/ pre-transplant LVEF affected the extent of recovery as was demonstrated in Fig. S6E for n=3-4 animals per group. Therefore, we cannot completely rule out the possibility that the therapeutic effect is not apparent in the Ruby-treated animals due to the less pronounced reduction in heart function post MI.
However, despite these limitations, the obvious positive effect on LVEF recovery after Amber-hiCMA transplantation is in stark contrast to the lack of recovery after the injection of Ruby-hiCMAs, which is essentially identical to the no-cell control animals despite the substantial engraftment of Ruby-hiCMAs shown in the revised manuscript (**new Fig. 5C-E, new Fig. S5 D, E**).
Our *in vitro* data clearly show significant functional differences concerning contractility and force development of Amber- vs. Ruby-derivatives. Combined with our extended cellular and molecular data on the reporter transgene effects in CMs and BCTs (as discussed in detail in reply to the reviewer's question #3 above), we consider the observed transgene-dependent differences as the most likely reason for the lack of functional improvement after the transplantation of Ruby-derived CMs in contrast to the observed heart recovery Amber-hiCMA transplantation. According to the reviewer's suggestion we have therefore toned down this claim in the modified title.

8) The authors state that "No apparent differences were observed for graft integration and marker expression of CMs derived from the two reporter cell lines" (page 11, line 253). This statement should be supported by representative images from both experimental groups stained with the same set of markers as well as quantitative data (histomorphometry). The authors' graft RNAseq data suggest that there should be some differences.

In reply to reviewer's notion, we have amended the characterization of cultured hiCMAs via IF for classical cardiac markers including sarcomeric actinin, cardiac troponin T, GATA4, MLC2v and MLC2a; these images confirmed the high similarity of Amber- vs. Ruby-derived CMs based on the analysis of hiCMA sections (see **amended Fig. 1C-J, new Fig. S1**).
Regarding the characterization of human grafts in primate hearts, we apologize that, in the originally submitted manuscript, no relevant information was included in the legends of figures 4 and 5 specifying the source of grafted cells shown in each individual sub-figure; we have now included this lacking information in the respective legends of the revised manuscript.
Moreover, additional **new images** were included showing human troponin I and sarcomeric actinin staining for Ruby grafts in the **amended Fig. 5**.
Notably, no apparent differences in terms of graft structure and the expression of the analyzed cardiac marker proteins could be detected via immunostaining for troponin T, Titin, GATA4, MLC2v, MLC2a, sarcomeric actinin or connexin 43 (Figure 5).
However, as anticipated by the reviewer's insightful comments, our extended RNASeq data analyses revealed striking differences in gene expression patterns between Amber- and Ruby-BCTs and for the corresponding hiCMA-derived myocardial grafts as well. While the mRNA expression of genes belonging to the GO terms of cardiac muscle contraction and muscle filament sliding in Amber grafts was very similar to native heart muscle, the analyzed Ruby grafts showed a substantially different expression pattern (**revised Fig. 6D**).

Even more clear differential gene expression patterns were observed for Ruby- vs. Amber-derived BCTs; these patterns provide molecular hints toward the reasons for lower forces observed in Ruby BCT supporting our hypothesis that the different results on heart function recovery after cell transplantation indeed reflect the functional properties of Amber- vs. Ruby-derived CMs.

9) Gruh et al also claim that hiCMAs formed grafts “comparable” to those obtained historically with the delivery of 10-20-fold more iPSC-derived cardiomyocytes in single-cell suspensions. However, quantitative data are lacking. The manuscript does include one example of a respectably sized graft (Fig. 5B), but the authors make no attempt to actually measure graft size by histomorphometry. (By contrast, the aforementioned study from the Fukuda group reports graft size as a percentage of LV and infarct area.) It would be helpful to include quantitative measurements of graft area for each cell recipient. This would be ideally broken down by zone since nearly all provided images show graft tissue implanted in host myocardium, not scar tissue.

Given the reviewer’s important request, we have now applied classic histomorphometry of 2D images after IF and 3D imaging of paraffin embedded tissue samples using a laboratory X-ray µ-CT to quantify exemplary grafts. Tissue sections of hearts were stained for human-specific cardiac troponin I and positive areas were either quantified on 2D images and/ or used as a basis for the segmentation of grafts visible on µ-CT images prior to the 3D reconstruction and volumetric analysis (**see new/ amended Figs. 5B-E and S5C&D**).

Analyzing heart samples at 3 months from both Amber- and Ruby-hiCMA injected animals, we calculated human graft volumes of about 6-8 mm^3 for animal #07 (Amber recipient) and comparable dimension of about 5.6 mm^3 and 17.2 mm^3 for Ruby recipient animals #12 and #13, respectively.
When assessing the human CMs density on respective sections, again similar results for grafts from both cell lines were identified revealing a mean of 1,795 ± 151 CM/mm^2 as determined by the number of nuclei of titin-positive cells in the graft area (**new Fig. S5E**). Based on these data, the estimated number of engrafted hiPSC-CMs were ~650,000 for the ‘*Amber’* grafts in animal #07, compared to 502,769 (± 56,956) and 1,539,729 (±174,428) human CMs for *‘Ruby’* grafts in animals #12 and #13, respectively. This corresponds to an average of about 880,000 human CMs/ per cynomolgus heart and a graft tissue density of about 90,000 human CMs/ mm^3. Regarding the cell engraftment efficiency, roughly 1.8% of the 50 mio injected cells were recovered.
Due our sample collection strategy (defined when initiating the study), unfortunately, entire hearts were not available for compiling statistically verified data e. g. on the graft size relative to the left ventricle area or to the infarct area. Moreover, our practical experience revealed that reliable graft quantification and meaningful comparison to published data in the field pose a great challenge, given the different methods of sample collection, analysis and quantification, in individual studies.

Liu et al. [8] published a relatively high graft volume of 370 mm^3 and a high average amount of 90.3 mio human cells/ per heart (calculated from 4 animals assessed), which corresponds to about ~12% compared to the 750 mio injected single cells. Interestingly, a graft cell density of about 242,000 nuclei/ mm^3 can be calculated from these data, which is about 2.7fold higher (!) compared to our observations and may indicate a highly immature, potentially proliferative

status of the grafted cells in the Liu et al. report. Moreover, the grafts' dimensions in that study were calculated based on defining the tissue wet weight, which, depending on the applied experimental procedures, may induce substantial systematic experimental variance. However, notably, our functional heart recovery resulting from Amber-CMs applications seems equivalent to Liu et al. regarding the LVEF data, irrespective of the divergent graft size assessment technologies and results.

In the recent study by Kobayashi et al [9] injecting 60 mio cells (in the highest dose tested) as aggregated CMs (i.e. comparable to our approach), the human graft sections in their Figure 4a seem to have highly equivalent dimensions compared to our results in Fig. 5. Unfortunately, in that study from the Fukuda group, we did not find information on absolute graft sizes (i.e. in mm^3 and CM numbers) but only relative graft areas in % of scar area or left ventricular wall area, respectively; but the functional improvement in the study was also comparable to our results.

Finally, we like to highlight to this reviewer that we have mapped the localization of detected human grafts in individual NHP heart samples for each animal to a 17-segment model of the human left ventricle [9], noting that the "*Grafts were located in particular outside the infarct area and mostly in apical segments of the left ventricle including the apex itself*" (see p. 12, lines 6-7 of the amended manuscript; **new Fig. 5, S5A, B**).

10) Similarly, one also wonders whether the apparently different functional outcomes between Amber vs Ruby hiCMAs may simply reflect differences in graft size. It seems entirely plausible that Ruby cardiomyocytes had lower viability and just formed smaller grafts (and perhaps less healthy cardiac tissues in Fig. 2), leading to less contractility recovery and fewer arrhythmias.

As discussed in detail in reply to the reviewer's question #9 above, we now provide quantitative examples of human grafts for both Amber- and Ruby-hiCMA injected hearts, revealing comparable graft sizes irrespective of the reporter cell line used. This clearly argues against smaller Ruby graft sizes as the underlying reason for the lack of heart function recovery and the lack of arrhythmias as well. Please see images of the graft quantification in **new Figs. 5B-E** and **S5C&D** and the respective paragraphs in "Results" and "Discussion".

11) Why does the duration of ECG monitoring vary so much by animal in Figures 7B-D? If I understand the experimental design correctly, each animal should have been monitored continuously to either 2 weeks or 3 months post-transplantation.

The reason for different duration of ECG monitoring includes technical issues, particularly premature battery discharge. We have now integrated this information into legend of Fig. 7: "white: no data acquired due to technical issues, including premature end of recording due to reduced battery lifespan" (see p. 50, line 9-10).

12) Do the authors have any speculation as to why the onset of arrhythmias was later in their study than in previous reports? This reviewer wonders whether that simply reflects smaller graft sizes, but there may also be differences in electrotonic source-sink with aggregates versus single cells.

As already discussed in our reply to the reviewer's comment #10 above, our new data in the amended manuscript reveal equivalent graft size in Amber- vs Ruby-treated animals, arguing against a size-dependent reason for the lack of arrythmias in Ruby-CM injected hearts.
However, as suggested by the reviewer, we have compared our data concerning the appearance of cell-injection related arrhythmia with several other studies [8, 10, 11, 12, 13]. The published occurrence of arrhythmias, their type, frequency, and duration vary substantially between the different studies. In addition, some studies did not apply antiarrhythmic drugs or delivered just an initial bolus of lidocaine, whereas others applied amiodarone. While some studies reported an immediate onset of arrhythmia after MI or after cell injection (at least in individual animals), Chong et al. [10] and Liu et al. [8] also reported (individual) animals with a delayed appearance of arrhythmias.
In our case, the delayed onset of arrythmia is most likely due to the administration of the anti-arrhythmic drug amiodarone, which was administered for 5 days before and 9 days after cell administration. This drug has been applied only in one other study by Chong et al. Although highly variable, a reduced antiarrhythmic effect of amiodarone can persist for up to 6 months [14] (discussed also in the revised manuscript p. 23, line 24-27).
Our RNAseq analysis suggested a lower expression of the gap-junction marker Cx43 in Amber- vs. Ruby-derivatives, which may suggest a delayed onset of functional cell-cell connections between engrafted Amber-CMs with the host tissue, tempting us to speculate that this maybe another mechanism underlying the delayed onset of graft-induced arrythmias observed in our study.

Specific comments:

1) In Fig. 1B, why were aggregate sizes omitted for other hiCMA batches that were apparently transplanted (#01, #02, #04)?

We apologize for not having included aggregate sizes for animals #01 and #02. Animal #04 received the same cells than animal #3, which was also not clarified. The requested information has now been included in the revised manuscript, only one value is not available (time-point CP1 for animal #01) and has been labeled as such in the **amended Fig. 1B**.

2) For multiple figures (for example, Fig. 1C-H), it would be better to show representative data for both the Ruby and Amber conditions. If space is limited, one set could be included in the supplement. Moreover, in a number of instances in Figs. 1, 4, and 5, it is not stated which iPSC line was used to generate the presented data.

Figure 1 was revised according to the reviewer's comment. Ruby and Amber data are now shown side by side. Immunostainings of aggregates at CP1 are shown in the **new Fig. S1A-F** for both cell lines. For figures 4 and 5, legends have been complemented with the requested information. Additional images for Ruby grafts were included in the **amended Fig. 5**.

3) Sarcomere alignment is not apparent in Fig. 2B.

We have now modified the image in the **amended Fig. 2B** to include a magnification of the staining as inset with visible cross striations.

4) Maybe this is just from PDF compression, but there appear to be breaks in the force trace in panel 2G.

We apologize for this technical problem. We have now replaced the force traces in the **revised Figure 2** by new graphs showing exemplary force traces at different pacing frequencies and provide additional graphs with a full force-frequency relationship analysis in the **new Fig. S2A, B.**

5) Scale bars are missing in panels 2E and 2H.

We have now integrated new images into **revised Figure 2** to allow direct comparison of Amber-BCTs with Ruby-BCTs. On all images, there are now scale bars included.

6) Cardiac MR images depicted in Fig. 3B are relatively low quality, and it is not at all evident what is even being pointed to in the post-contract images. Perhaps the poor contrast in the latter is because this was a permanent infarct model? Do the authors have short axis views pre- vs post-contrast?

We have now replaced the images in the **amended Fig. 3B** with new ones with higher resolution and adjusted color scale. As described in the legend, pre-contrast (above) and post-contrast (below) maps of T1-relaxation time [15] (see color scale) clearly visualize the fibrotic scar region (white arrows) in the septum and the apex of the myocardium. Additional images of short axis views were added to the **new Fig. S3**.

7) The legend for Fig. 3B indicates that the infarct examples were at 14-days post-MI, but the Supplemental Methods state that these were 3-month post-MI.

In the long-term survivors (groups B1/B2 and C, see also Fig. 3D), MRIs were conducted 2 and 12 weeks after cell tx / sham treatment. **Revised Fig. 3B** shows MRI images 2 weeks after cell Tx.

8) It would be clearer to refer to the “sham” experimental group as “vehicle” recipients. These animals underwent MI but not hiCMA transplantation, while sham usually no procedure whatsoever.

According to literature, a sham group in experiments refers to a control group that undergoes a procedure or intervention designed to mimic the active treatment as closely as possible, but without delivering the therapeutic component under investigation. In surgical or procedural trials, a sham intervention may involve all steps of the procedure except the critical therapeutic element allocation [16, 17, 18, 19, 20]. We have clarified in **revised Fig. 3D** (table and legend) that the sham treatment comprised the injection of the vehicle PBS.

9) It would be interesting to know whether serum TnT (or other biomarkers) correlated with infarct size by either MRI or histology.

As depicted in **revised Figure 3**, the best correlation of functional impairment (changes of LV-rEF) as determined by MRI at day 14 was observed with TnT serum levels measured at day 1 post MI. Serum levels of CK, CKMB and also CKMB/CK (now in Fig. S3E) correlated less good than TnT serum levels (**revised Fig. 3F**). The **new Fig. 3G** shows that TnT serum levels correlated with scar size as well.

10) It would be interesting to have the authors' perspectives about how TnT levels might be used in this animal model in the future. For example, could early TnT measurements serve as inclusion-exclusion criteria (which were badly needed in the present study)?

We fully agree with this reviewer's comment and have incorporated an additional sentence in the discussion: "*Thus, early cTnT measurements could serve as inclusion-exclusion criteria in future NHP experiments.*" (see p. 22, lines 17-18 in the amended manuscript)

11) What is meant by "in principle GMP-compliant conditions" (page 16, line 269)?

We thank the reviewer for this comment and appreciate the opportunity to clarify this point. With this phrase we intend to convey that - while the reagents used in the present protocol are not GMP-grade - equivalent GMP-quality reagents and GMP-compliant process strategies are available for all critical components and process steps. Thus, the overall workflow could be transferred to a full-GMP setting in principle, provided that appropriate GMP-grade substitutes are implemented. A more detailed discussion of GMP considerations and reagents substitution has also been provided in our recent publication Kriedemann et al. [4].

12) Outcomes are reported by animal in Table 1, but it would be more transparent to plot the relevant raw data, i. e. "raw" LVEF values not just normalized LVEF as currently done in Figs. 6 and S3.

Due to technical reasons (as detailed above in our answers to this reviewer's question #7) we decided to determine heart function as LVEF relative to the pre-MI LVEF (LV-rEF), as this normalization increased the comparability of results in the relative small group of animals available.
To provide a transparent overview of the data "raw" LVEF values and LV-rEF values are now reported side-by-side in **revised Table 1** to show pre- and post-MI variation. Raw values for LVEF as well as LV-ESV and LV-EDV are also given in the Fig. S3C, D (previous Fig. S1B). However, for any correlation analysis of comparison between animals and groups, LV-rEF was used (Fig. 3F, Fig. S3E, Fig. 6A, B and Fig. S6C-E).

13) Statistical methods should be clarified for each figure; in some cases (e.g., Fig. 1L), it is not stated whether or what statistical comparisons were performed.

We have included the requested information about statistical methods used in the respective legends throughout the manuscript and the supplemental material.

Reviewer #2:

The authors revealed the safety and efficacy of the transplantation of human iPSC cardiomyocyte aggregates into the hearts of cynomolgus monkeys with myocardial infarction. They also showed that its effectiveness correlates with the iPSC clone-dependent contractile function of cardiomyocytes. These findings will contribute to the future development of cell transplantation therapy using human iPSC-derived cardiomyocytes. However, in some experiments, the presented data is insufficient. The authors need to adequately address the following concerns:

Major

1. In Figure 1, hiCMAs from CP1 and hiCMAs from CP2 are compared. Regarding the immunostaining microscopic images in Figure 1E-H, the authors should present microscopic images of hiCMAs from CP1 and CP2 for each antibody. Additionally, do the authors argue that key cellular and physical properties of hiCMAs are maintained in CP2 after long-term culture, as stated in Lines 141-143? Culturing for a longer period would be disadvantageous from both a cost and time perspective. If the authors suggest that hiCMAs from CP2 are more mature than those from CP1, and that using more mature hiCMAs would be better for cell transplantation and BCT formation, it should be clearly stated.

We have now adapted **Fig. 1** according to the reviewer's request. Using the same antibodies, additional IF staining was performed allowing for the direct comparison of hiCMAs derived from Amber versus Ruby side by side. Because of space restrictions in the main figure, we have focused on the CP2 comparison in the **amended Fig. 1** given that the hiCMAs were collected at CP2 and applied for functional studies at this stage; corresponding immunostainings of CP1 hiCMAs for both reporter lines are now shown in the **new/amended Fig. S1**.

Regarding the extended cultivation of hiCMAs, we agree with the reviewer that prolonged culture is both time- and cost-intensive. However, in our current workflow, maintaining hiCMAs in culture until their functional testing deems more feasible than cryopreservation as no sufficiently robust cryopreservation protocols for cell aggregates have been available at the initiation of our study. Moreover, this limitation is generally not sufficiently resolved in the cell therapy field at present and remains a future challenge.

In our current study, however, the extended culture period represents a practical approach for bridging the gap between the "cell product generation" and its application to our complex animal model, equivalent to the future transition from the cell production site to the bed site.

This in mind, we would like to emphasize that we do not claim that CP2 hiCMAs are more mature than CP1 nor that the extended cultivation per se is beneficial for hiCMA transplantation to the heart or for BCT formation; if this impression arose, it was unintentional. Our statement in the former manuscript version was solely referring to the maintenance of key cellular and physical hiCMA properties during extended culture, but not to cells' functional maturation.

2. In Figure 2, Amber-BCT and Ruby-BCT are compared. Immunostaining for SA is performed on Amber-BCT in Figure 2B and immunostaining for cTnT is performed on Ruby-BCT in Figure 2E. The authors should present microscopic images of Amber-BCT and Ruby-BCT at the same scale, where immunostaining was performed using the same antibody for comparison. Additionally, it would be meaningful to compare the profiles of human iPSC-derived cardiomyocytes contained in Amber-BCT and Ruby-BCT through immunostaining for MLC2a/MLC2v, etc..

Thanks to reviewers' suggestions, additional images showing immunostaining for Titin have been included in the **amended Fig. 2** to enable a direct comparison of BCTs derived from Amber vs Ruby CMs. Just like for the comparison of hiCMAs from Amber vs Ruby in **revised Fig. 1 and Fig. S1**, immunostaining did not reveal apparent differences in expression profiles. Therefore, we chose a more thorough comparison of the profiles of human iPSC-derived CMs contained in Amber-BCT and Ruby-BCT via RNA-Seq (Fig. 6C, D; **new Fig. S7B** and **new Fig. S8E**) and patch clamp analysis (**new Fig. S1G-N** and **new Fig. S8A-D**).

3. In Figure 5, the microscopic images of immunostaining using each antibody are only presented for either the Amber-treated group or the Ruby-treated group. Considering the results showing that Amber-hiCMAs and Ruby-hiCMAs had different effects on the improvement of cardiac function, the authors should present and compare the microscopic images of the grafts from each group. For example, it should be demonstrated whether there are any differences in angiogenesis between the Amber-hiCMA grafts and the Ruby-hiCMA grafts.

We apologize that in the initially submitted manuscript version no information was included in the legends of Fig. 5 that specifies the source cells of the grafts shown in each individual sub-figure. We have now included the lacking information in the respective legends.
Additional images were included that show human troponin I and sarcomeric actinin stainings of Ruby grafts in the **amended Fig. 5**. No differences in terms of graft structure or expression of the analyzed cardiac markers proteins could be detected via immunostaining for cardiac troponin T, MLC2v, MLC2a, sarcomeric actinin or connexin 43.
Concerning differences in angiogenesis, host-derived $CD31^{+}$ were detected in Amber grafts already after 2 weeks (Fig. 4I) and in Ruby grafts after 3 months (Fig. 5J, no earlier data available for Ruby). A quantification of $CD31^{+}$ cells in the graft area was included as **new Fig. S5F** and indeed showed a tendency for a lower endothelial density in two Ruby grafts compared to age-matched Amber grafts.

4. The authors concluded that Amber-hiCMAs improved cardiac function, whereas Ruby-hiCMAs did not. They noted that in monkeys with large impairment induced by myocardial infarction, a slight recovery was observed even in the sham group after three months. In the Ruby-hiCMAs-treated group, the extent of cardiac function decline after myocardial infarction was less than that in the Amber-hiCMAs-treated group. Could this be related to the lack of improvement in cardiac function in the Ruby-hiCMAs-treated group?

As already discussed in response to question #7 of reviewer #1, the small numbers of animals typically included in NHP studies (due to apparent financial, regulatory and ethical restrictions) has led to additional challenges for demonstrating functional heart recovery after induced MI.
Already for pre-MI LVEF values some variations were observed, most likely due to the expected inter-individual differences in animals' age, weight etc.. However, the extremely low pre-MI values for animals #05 and #10 (**revised Table 1**) can be explained by technical reasons: these two animals were evaluated using the Real-Time approach due to distorted ECG signals, while standard CINE MRI was used for assessing all other animals.
While induction of MI was successful in most animals with a loss of contractile myocardium in left ventricular and septal areas near the apex, there was indeed a substantial degree of variance in LVEF after MI (**revised Table 1**). These differences most likely reflect different

sizes of ischemic and resulting infarct areas due to inter-individual differences in coronary artery anatomy [7] and positioning of the coronary artery ligation as well.
To account for these limitations, we decided to determine heart function as LVEF relative to the pre-MI LVEF (LV-rEF), as this normalization increased the comparability of results in the relatively small group of animals.
However, despite expressing heart function as LV-rEF, the higher average LV-rEF after MI in the Ruby vs Amber animals is still most likely just reflecting the given limitations of the low number of animals that could be included on our study.
In line with the reviewer's questions, we cannot fully rule out the possibility that the therapeutic effect is not apparent in the Ruby-treated animals due to the less pronounced reduction in heart function. However, since our *in vitro* data clearly indicate significant functional differences concerning contractility and force development of Amber- and Ruby-derived CMs, which is now further substantiated by cellular and molecular data in the amended manuscript, we consider that the lack of functional improvement after transplantation of Ruby-CMs is indeed an iPSC-line/ reporter transgene-dependent effect. Vice-versa this substantiates the assumption that Amber-derived grafts do support heart recovery by their specific, contractile functionality.

5. The transplantation of Ruby-hiCMAs resulted in less improvement in cardiac function and a lower incidence of arrhythmia compared to the transplantation of Amber-hiCMAs. These results could be explained if the engraftment rate in the Ruby-hiCMAs-treated group was lower than that in the Amber-hiCMAs-treated group. Were there any differences in the engraftment rates between the two groups?

Thank you for raising this important topic. Although we could only exemplarily quantify the engraftment of human CMs in some animals (please see detailed reply to question #9 of reviewer #1 above), our new histomorphometry data in the amended manuscript revealed the formation of equivalent grafts from both reporter iPSC lines (**see new/ amended Figs. 5B-E and S5C&D**). As discussed in the detailed response to question #3 of reviewer #1, our data also strongly suggest that the observed functional differences between Amber and Ruby are caused by the expression of the nuclear RedStar in Ruby-CMs. These findings clearly argue against a smaller Ruby graft size as an underlying reason for both the lack of heart function recovery and the lack of arrhythmia after the injection of Ruby-derived iCMAs (also described and discussed in respective paragraphs in "Results" and the "Discussion").

6. What might be the reason for the lower frequency of arrhythmias in the Ruby-hiCMAs-treated group?

There is a long-term debate ongoing in the field about the origin of engraftment-related arrhythmias after transplantation of pluripotent stem cell-derived cardiomyocytes. In particular, the potential contamination of iPSC-CM preparations with pacemaker-like cell phenotypes has been discussed as well as the general immaturity of differentiated CMs.
In our amended study, patch clamp analyses revealed that both Amber- and Ruby-derived CMs predominantly represent typical immature ventricular-like characteristics. The immature action potential (AP) patterns are in line with the observed heterogenous expression of MLC2v/ MLC2a, which has been described for fetal CMs during native heart development [21].
A recent study by Marchiano et al. [22] notably suggests that the immature phenotype of hPSC-derived ventricular-like CMs – rather than contaminating pacemaker-like cells - is causing the

engraftment-related arrhythmias. It seems that the depolarization-associated, ion channel-encoding genes HCN4, SLC8A1, and CACNA1H, which have been knocked-out by Marchiano et al. in hESCs-CMs to overcome graft-associated arrhythmias in a pig model, notably all show low expression levels in Ruby-CM-derived BCTs, while the hyperpolarization-associated gene KCNJ2 (overexpressed by Marchiano et al.) is variably expressed between Amber- and Ruby-BCT samples (**new Fig. S8E**).
Notably, since also gap junctions and thus Connexins likely play a decisive role in the induction of arrhythmia by the grafted cardiomyocytes, we have analyzed gap junction proteins in Amber- and Ruby-BCTs. We observed upregulation of Connexin 43 (Cx43; GJC1) in Ruby BCTs, while Connexin 45 (GJA1) is downregulated; expression of Connexin 40 (GJA5, not shown) is variable between samples (**new Figure S8E**).
Together, this suggest that transplanted Ruby-hiCMAs may actually couple to the host myocardium (potentially even more efficient than Amber-hiCMAs due to their upregulated Cx43) but apparently do not induce engraftment arrhythmias due to their low expression of depolarization-associated genes HCN4, SLC8A1, CACNA1H as well as the dominant sodium channel-encoding genes SCN5A, SCN4B and others (**new Figure S8E**).
In contrast, Amber-hiCMAs can apparently transmit their autonomous depolarization activity (associated with their developmental immaturity) to the host myocardium despite their reduced Cx43 expression (relative to Ruby-CMs); thus, the reduced Cx43 expression in Amber-derivatives may suggest that a relevant electrophysiological connection to the host myocardium requires time to establish. In addition to the administration of the anti-arrhythmic drug amiodarone in our animal model, this may also explain the relative late onset of arrhythmogenicity starting not before 2 weeks after the transplantation of Amber-hiCMAs as we have noted in reply to the reviewer's questions above.

7. The authors showed that Amber-BCTs exhibited stronger contractile function than Ruby-BCTs. RNA sequencing also revealed that gene expression related to cardiac muscle contraction and muscle filament sliding was upregulated in Amber-BCTs compared to Ruby-BCTs. These findings were suggested as the reasons why Amber-hiCMAs had a greater effect on improving cardiac function than Ruby-hiCMAs. In cell transplantation therapy using human iPSC-derived cardiomyocytes, both the direct contractile function of the transplanted cardiomyocytes and paracrine effects are considered to be the mechanisms for the improvement in cardiac function. Were there any differences in the paracrine effects between the two groups?

According to the reviewer's suggestion we have now analyzed potential differential expression of paracrine factors in Amber vs. Ruby hiCMAs and in BCTs derived from both lines. Respective results are now expressed in Results (p. 15/16 of the amended manuscript) as follows:
"To explore potential paracrine effects of the grafts, we have analyzed the differential expression of candidate factors in 'Amber' vs. 'Ruby' hiCMAs and in BCTs derived from both lines based on a list of 613 genes that have been associated with myocardial infarction. Differential expression in 'Amber' vs. 'Ruby' derivatives is shown in the heatmap in ***Fig S6C*** *whereby 12 genes encoding for secreted factors (highlighted in green letters; including IL6, LIF, EDN1, SCF2, WNT5A and others) were upregulated in 'Ruby' and 6 (i.e. NPPB, APLN, APOA1, VEFGA, IL33, and AGT) in the respective 'Amber' samples.*

Since numerous of these factors such as Vascular endothelial growth factor (VEGF)-A, Apelin (APLN), Apolipoprotein A1 (APOA1), Interleukin 33 (IL-33) have been associated with cardio-

protection or heart function improvement, we cannot exclude paracrine effects resulting from our hiCMA injection."

8. Related to the above, regarding the results of RNA sequencing, the authors should present not only the heatmap of specific genes shown in Figure 6CD but also the results of the GO analysis.

The results of the GO analysis has now been included in the **new Figure S7A**.

9. In Table 1, #05 and #10 are annotated as "lower absolute values for MRI parameters due to analysis with real-time mode". #05 was excluded from the analysis in Figure 6, along with #14, due to very small LVEF changes after MI. Is #10 an appropriate subject to include in the analysis?

The analysis of MRI data using the real-time mode generally yields lower values for the LVEF. Therefore, direct comparison of corresponding data points of animals #5 and #10 and data points of the other animals is not possible. Besides the varying infarct size, also this reason prompted us to show relative (LV-rEF) values instead of absolute LVEF values in **revised figure 6B** (please also see our detailed discussion of this topic in reply to question #7, Reviewer #1 above).
Animals #5 and #14 were excluded from the analysis because they showed minor changes of LVEF after MI (-1.1%pts for #5 and -3.6%pts for #14) indicating induction of small infarct areas, only. In contrast, the change in LVEF in animal #10 was -9.3%pts, indicating an MI of substantial size.

10. The ischemia/reperfusion model is more clinically relevant than the permanent ligation model. Why did the authors choose the permanent ligation model in the present study?

We agree with the reviewer that the ischemia/reperfusion model indeed is a better reflection of the clinical situation of human patients and we had considered this option before the initiation of our study. However, we chose the permanent ligation model for a number of reasons:
This approach was recently successfully used in non-human primates to investigate the effect of transplantation of stem and progenitor cells or cardiomyocytes [23], and enabled us to induce a technically well controlled, permanent damage with relatively large infarction sizes to serve as a 'worst-case scenario' in our proof-of-concept study on heart regeneration.
To be fully transparent, practical reasons played a role as well. First, the regulating authorities were concerned about the overall duration of the procedures and thus anaesthesia for the monkeys. While common protocols for ischemia/ reperfusion describe an artery occlusion time of 3h, we were able to perform the complete surgery (including left-lateral thoracotomy) for permanent ligation in 1h or less. Second, at that time, a fully equipped catheter lab needed for an endovascular approach was not available at the German Primate Centre in Göttingen, again favouring open chest surgery and calling for a faster procedure.

Minor
1. HF, which is an abbreviation of heart failure, is used only in Line 62 and not in other sections.

HF is now used as abbreviation throughout the manuscript.

2. The notation for the Figure in the text is inconsistent, with some instances using Figure and others using Fig. Please make it consistent.

The notation of "Figure" in the text is now used throughout the text in the abbreviated form "Fig."

3. In the hiCMA B2 Ruby group of Figure 6B, some graphs are missing error bars indicating standard deviation and should be corrected.

Figure 6B has been revised and missing error bars have now been added.

References:

1. Kempf H*, et al.* Bulk cell density and Wnt/TGFbeta signalling regulate mesendodermal patterning of human pluripotent stem cells. *Nature communications* **7**, 13602 (2016).

2. Kempf H, Kropp C, Olmer R, Martin U, Zweigerdt R. Cardiac differentiation of human pluripotent stem cells in scalable suspension culture. *Nat Protoc* **10**, 1345–1361 (2015).

3. Halloin C*, et al.* Continuous WNT Control Enables Advanced hPSC Cardiac Processing and Prognostic Surface Marker Identification in Chemically Defined Suspension Culture. *Stem Cell Reports* **13**, 775 (2019).

4. Kriedemann N*, et al.* Standardized production of hPSC-derived cardiomyocyte aggregates in stirred spinner flasks. *Nat Protoc*, (2024).

5. Chen TH*, et al.* Cardiac fibrosis in mouse expressing DsRed tetramers involves chronic autophagy and proteasome degradation insufficiency. *Oncotarget* **7**, 54274–54289 (2016).

6. de la Roche J*, et al.* Comparing human iPSC-cardiomyocytes versus HEK293T cells unveils disease-causing effects of Brugada mutation A735V of Na(V)1.5 sodium channels. *Sci Rep* **9**, 11173 (2019).

7. Teofilovski-Parapid G, Kreclovic G. Coronary artery distribution in Macaca fascicularis (Cynomolgus). *Lab Anim* **32**, 200–205 (1998).

8. Liu YW*, et al.* Human embryonic stem cell-derived cardiomyocytes restore function in infarcted hearts of non-human primates. *Nat Biotechnol* **36**, 597–605 (2018).

9. Kobayashi H*, et al.* Regeneration of Nonhuman Primate Hearts With Human Induced Pluripotent Stem Cell-Derived Cardiac Spheroids. *Circulation* **150**, 611–621 (2024).

10. Cerqueira MD*, et al.* Standardized myocardial segmentation and nomenclature for tomographic imaging of the heart. A statement for healthcare professionals from the Cardiac

Imaging Committee of the Council on Clinical Cardiology of the American Heart Association. *Circulation* **105**, 539–542 (2002).

11. Chong JJ*, et al.* Human embryonic-stem-cell-derived cardiomyocytes regenerate non-human primate hearts. *Nature* **510**, 273–277 (2014).

12. Guan X*, et al.* Human induced pluripotent stem cell-derived cardiomyocytes improve recovery from myocardial infarction in non-human primates. *Stem Cell Res Ther* **16**, 581 (2025).

13. Shiba Y*, et al.* Allogeneic transplantation of iPS cell-derived cardiomyocytes regenerates primate hearts. *Nature* **538**, 388–391 (2016).

14. Drug Label Information: AMIODARONE HYDROCHLORIDE. In: *Dailymed*). National Institutes of Health, National Library of Medicine.

15. Freedman MD, Somberg JC. Pharmacology and pharmacokinetics of amiodarone. *J Clin Pharmacol* **31**, 1061–1069 (1991).

16. Galpern WR*, et al.* Sham neurosurgical procedures in clinical trials for neurodegenerative diseases: scientific and ethical considerations. *Lancet Neurol* **11**, 643–650 (2012).

17. Horng S, Miller FG. Ethical framework for the use of sham procedures in clinical trials. *Crit Care Med* **31**, S126–130 (2003).

18. Albin RL. Sham surgery controls: intracerebral grafting of fetal tissue for Parkinson's disease and proposed criteria for use of sham surgery controls. *J Med Ethics* **28**, 322–325 (2002).

19. Tambone V*, et al.* A Proposed Road Map for the Ethical Evaluation of Sham (Placebo) Surgery. *Ann Surg* **265**, 658–661 (2017).

20. Hohenschurz-Schmidt D*, et al.* Blinding and sham control methods in trials of physical, psychological, and self-management interventions for pain (article II): a meta-analysis relating methods to trial results. *Pain* **164**, 509–533 (2023).

21. Small EM, Krieg PA. Molecular regulation of cardiac chamber-specific gene expression. *Trends Cardiovasc Med* **14**, 13–18 (2004).

22. Marchiano S*, et al.* Gene editing to prevent ventricular arrhythmias associated with cardiomyocyte cell therapy. *Cell Stem Cell* **30**, 396–414 e399 (2023).

1. Halloin C*, et al.* Continuous WNT Control Enables Advanced hPSC Cardiac Processing and Prognostic Surface Marker Identification in Chemically Defined Suspension Culture. *Stem Cell Reports* **13**, 775 (2019).

2. Kempf H, Kropp C, Olmer R, Martin U, Zweigerdt R. Cardiac differentiation of human pluripotent stem cells in scalable suspension culture. *Nat Protoc* **10**, 1345-1361 (2015).

3. Kempf H*, et al.* Bulk cell density and Wnt/TGFbeta signalling regulate mesendodermal patterning of human pluripotent stem cells. *Nature communications* **7**, 13602 (2016).

4. Kriedemann N*, et al.* Standardized production of hPSC-derived cardiomyocyte aggregates in stirred spinner flasks. *Nat Protoc*, (2024).

5. Chen TH*, et al.* Cardiac fibrosis in mouse expressing DsRed tetramers involves chronic autophagy and proteasome degradation insufficiency. *Oncotarget* **7**, 54274-54289 (2016).

6. de la Roche J*, et al.* Comparing human iPSC-cardiomyocytes versus HEK293T cells unveils disease-causing effects of Brugada mutation A735V of Na(V)1.5 sodium channels. *Sci Rep* **9**, 11173 (2019).

7. Teofilovski-Parapid G, Kreclovic G. Coronary artery distribution in Macaca fascicularis (Cynomolgus). *Lab Anim* **32**, 200-205 (1998).

8. Liu YW*, et al.* Human embryonic stem cell-derived cardiomyocytes restore function in infarcted hearts of non-human primates. *Nat Biotechnol* **36**, 597-605 (2018).

9. Cerqueira MD*, et al.* Standardized myocardial segmentation and nomenclature for tomographic imaging of the heart. A statement for healthcare professionals from the Cardiac Imaging Committee of the Council on Clinical Cardiology of the American Heart Association. *Circulation* **105**, 539-542 (2002).

10. Chong JJ*, et al.* Human embryonic-stem-cell-derived cardiomyocytes regenerate non-human primate hearts. *Nature* **510**, 273-277 (2014).

11. Guan X*, et al.* Human induced pluripotent stem cell-derived cardiomyocytes improve recovery from myocardial infarction in non-human primates. *Stem Cell Res Ther* **16**, 581 (2025).

12. Kobayashi H*, et al.* Regeneration of Nonhuman Primate Hearts With Human Induced Pluripotent Stem Cell-Derived Cardiac Spheroids. *Circulation* **150**, 611-621 (2024).

13. Shiba Y*, et al.* Allogeneic transplantation of iPS cell-derived cardiomyocytes regenerates primate hearts. *Nature* **538**, 388-391 (2016).

14. Freedman MD, Somberg JC. Pharmacology and pharmacokinetics of amiodarone. *J Clin Pharmacol* **31**, 1061-1069 (1991).

15. Haaf P, Garg P, Messroghli DR, Broadbent DA, Greenwood JP, Plein S. Cardiac T1 Mapping and Extracellular Volume (ECV) in clinical practice: a comprehensive review. *J Cardiovasc Magn Reson* **18**, 89 (2016).

16. Albin RL. Sham surgery controls: intracerebral grafting of fetal tissue for Parkinson's disease and proposed criteria for use of sham surgery controls. *J Med Ethics* **28**, 322-325 (2002).

17. Galpern WR*, et al.* Sham neurosurgical procedures in clinical trials for neurodegenerative diseases: scientific and ethical considerations. *Lancet Neurol* **11**, 643-650 (2012).

18. Hohenschurz-Schmidt D*, et al.* Blinding and sham control methods in trials of physical, psychological, and self-management interventions for pain (article II): a meta-analysis relating methods to trial results. *Pain* **164**, 509-533 (2023).

19. Horng S, Miller FG. Ethical framework for the use of sham procedures in clinical trials. *Crit Care Med* **31**, S126-130 (2003).

20. Tambone V*, et al.* A Proposed Road Map for the Ethical Evaluation of Sham (Placebo) Surgery. *Ann Surg* **265**, 658-661 (2017).

21. Small EM, Krieg PA. Molecular regulation of cardiac chamber-specific gene expression. *Trends Cardiovasc Med* **14**, 13-18 (2004).

22. Marchiano S*, et al.* Gene editing to prevent ventricular arrhythmias associated with cardiomyocyte cell therapy. *Cell Stem Cell* **30**, 396-414.e399 (2023).

23. Zhu K*, et al.* Lack of Remuscularization Following Transplantation of Human Embryonic Stem Cell-Derived Cardiovascular Progenitor Cells in Infarcted Nonhuman Primates. *Circ Res* **122**, 958-969 (2018).

Reviewer #1 (Remarks to the Author):

The revised manuscript by Gruh et al is substantially improved, and this reviewer greatly appreciates the thoughtful rebuttal and interesting new data provided by the authors. The additional phenotyping of Amber versus Ruby CMs and new in vitro data with BCTs formed with other iPSC lines are particularly helpful. Findings in the revised manuscript will be of significant interest to the field, but there are a few minor remaining issues that would further strengthen clarity and impact:

We are thankful for the positive feedback to our revised manuscript and the helpful suggestions provided earlier which helped us to strengthen our study. We have addressed the remaining minor issues as follows:

1. Legends should be provided for all heat maps of gene expression (Figs. 5K, 6C-D, S7B, S8E, and S9). It is currently unclear whether the color scale in each represents Z-score, log fold-change, or something else entirely.

Indeed, the colour scale represents row-wise Z-score-scaled TPM values We have added the missing information to the respective figure legends.

2. While fully understanding the authors' rationale for normalizing LVEF to the pre-infarct LVEF (LV-rEF), this approach is a bit non-standard. It would be more transparent to separately plot both LV-rEF and "raw" LVEF values (with the latter at least included in a supplemental figure). This was recommended previously, but the authors have instead just shuffled around the ordering of the data in Table 1, leaving this work to the reader and making it harder to compare between transplantation studies.

We appreciate the reviewer's understanding of our reasoning. To facilitate the comparison for the reader, we now display the raw LVEF values in Figure 3c and provide a side-by-side comparison with the normalized LV-rEF in the supplemental figure S3c. We have added the following statement to the manuscript: "Due to the large variance of pre-MI LVEF, relative LVEF values normalized to pre-MI values (Fig. S3c) were used for comparative analyses."

3. In Figures 2E and K, the calcium transients across various pacing frequencies are squished, making it impossible to distinguish individual transients at higher frequencies. It would be helpful if the authors could adjust the time axis and horizontally expand or stack these traces for easier visibility.

Within the Figures 2e and 2k, original traces from force measurements for BCTs are displayed. It is true that the individual peaks cannot be discerned at higher pacing frequencies. However, this is a well-known phenomenon indicating that at supra-physiological frequencies, the tissue is no longer able to fully relax before the next pacing impulse, thus leading to "squished" force readings.

4. Arguably, one of the more interesting findings of this manuscript is the unexpectedly negative consequences of nuclear RedStar fluorescent protein expression on CM phenotype. The authors may want to consider highlighting this important "lesson learned" by adding mention of the fluorescent protein reporter issue in the abstract (space permitting).

In reply to reviewers` valuable notion, we have modulated the abstract as follows:

Abstract

This study demonstrates the successful production and injection of human iPSC cardiomyocyte aggregates (hiCMAs) into infarcted cynomolgus monkey hearts, resulting in substantial, structured human grafts three months after Tx. Transient graft-induced arrhythmias decreased over time. Both the arrhythmogenicity and the substantial heart function recovery in vivo notably seemed to correlate with iPSC clone-dependent contractile and electrophysiological CM properties in vitro. Overexpression of a red fluorescent reporter protein led to a dysregulated conduction and contraction machinery in yet engraftment competent CMs, providing an important tool to mechanistically understand and improve iPSC-based heart repair in preclinical models.

We demonstrate the logistically important, temporal uncoupling of CM production from transplantation (Tx). CMs aggregate Tx yielded results comparable to the reported Tx of 10-20fold higher numbers of dissociated hESC-CMs and suggests a higher degree of cell/ tissue maturation in cardiac grafts. Our study promotes reduced cell production costs, highlights the need for an in vitro potency assay, and shows a pragmatic new avenue for the clinical translation of hiPSC-based heart repair.

Reviewer #2 (Remarks to the Author):

The authors have generally responded appropriately to our concerns and have revised the manuscript accordingly. However, we have the following concerns regarding the authors' response to our Question 5.

1. The authors measured the volume and cell number of Ruby-hiCMA grafts and Amber-hiCMA grafts, and demonstrated that Ruby-hiCMA grafts are not smaller than Amber-hiCMA grafts. We are convinced that the lack of improvement in cardiac function and the absence of arrhythmias in the Ruby-hiCMA-treated group were not due to poor engraftment. However, the reported graft volumes—6–8 mm^3 for Amber-hiCMA graft, and 5.6 mm^3 and 17.2 mm^3 for Ruby-hiCMA grafts—appear quite small when considering the size of the cynomolgus monkey heart. One of the key points emphasized in this study is that contractile force of hiPSC-CMs differs among cell lines and correlates with the improvement in cardiac function after transplantation. Is it appropriate to attribute the difference in cardiac functional improvement between the Amber-hiCMA-treated and Ruby-hiCMA-treated groups solely to differences in the contractile force of these relatively small grafts?

We highly appreciate reviewers' insightful comments and questions, further stimulating a critical discussion of our data compared to the state in the art, as follows:

In the last decades, central question in the field of cardiac cell therapy development included: 1) investigating potential mechanism(s) underlying functional improvement after cells grafting and 2) relevant control experiments promoting proper interpretation of functional improvement of the heart.

Ideally, donor cell engraftment would replace the massive, disease-induced CMs loss in the heart including the replacement of scar tissue typically associated with ischemia-reperfusion induced heart failure. At present, it deems save to state that none of the currently published PoC studies have achieved this "ultimate" aim.

Typically, engrafted cells are integrated into the functional, perfused cardiac tissue, neither replacing the scar tissue nor representing an overall cardiac cell mass equivalent to the (experimentally induced) loss of functional cardiac tissue in respective animal models.

However, our study, as well as previous studies in the field including the ground breaking work by Liu et al. (1) and Kobyashi et al. (2), suggest a measurable positive input of the grafted CMs to heart function as compared to damaged control hearts typically treated with cell-free saline injections, only. This, however, raises the question whether saline-injection controls represent a fair, relevant comparison.

This in mind, our paper provides an important input to the field. Our findings that poorly functioning hiPSC-CMs (although they do form stable grafts for at least 3 month) do not show a tendency of functional cardiac improvement, suggest that these cells represent a highly relevant control, which is superior to both saline injections as well as the potential application of a non-related cell lineages (e.g. bone marrow-derivatives extensively tested in the clinic in recent decades (3)). Thus, although the limited size of our human CM grafts and their location mainly in the healthy myocardium of the primate hearts (unlikely replacing scarred tissue) is not intuitive for explaining their positive impact on heart function, our relevant comparison of functional vs. non-functional CMs promotes the rational that proper CMs (rather than any cell engraftment) indeed have the potential to improve the function of damaged hearts, although further investigations regarding the underlying mechanism(s) are required in future.

2. In this study, 50 million cardiomyocytes were transplanted as aggregates, with approximately 1.8% engraftment, resulting in the graft volumes described above. In contrast, in the study by Liu et al., 750 million cardiomyocytes were transplanted as single cells, with approximately 12% engraftment and a graft volume of 370 mm³. While it is true that this study achieved a comparable improvement in cardiac function with less than one-tenth the number of cells, the engraftment rate reported here is lower than that in the single-cell transplantation study by Liu et al. Does this appear to contradict the authors' suggestion that aggregate transplantation improves engraftment efficiency? We acknowledge that direct comparisons are challenging due to differences in culture conditions, transplantation methods, and analytical approaches across studies. In addition, the authors state "However, due to the limited number of animals that can be tested due to ethical, regulatory and financial restrictions, our as well as the Kobyashi study have not performed a direct comparison of the aggregate approach versus single cell administration." in the Discussion. Although Kobayashi et al. did not directly compare aggregate versus single-cell transplantation in primates, their mouse study (shown in the Supplementary Data) demonstrated that aggregate transplantation resulted in approximately threefold higher engraftment than single-cell transplantation. How do the authors interpret the difference in engraftment rates between aggregate and single-cell transplantation?

The reviewer is raising formidable questions. Reviewers detailed insights strongly suggests her/ his intimate knowledge of the experimental heterogeneity between individual studies, including the particular challenges underlying the quantification and cross-study comparison of methodologies and results regarding the stereoscopy and quantification of 3D graft dimensions and related cell numbers analysis.

As described in our revised manuscript, we have performed substantial efforts to quantify of grafts and discuss these results in comparison to prior studies. In consideration of the particular challenges related to such quantification approaches, and of the influence of CM retention, survival and proliferation on long term engraftment, we are sceptical about the comparability of the collected data and the numbers of engrafted cells.

For example, one may expect that the injection of the massive cell quantity of 750 million cardiomyocytes per macaque heart (1) will induce a substantial and potentially detrimental congestion of the injected hearts. Remarkably, based on our own experimental evidence from our recent production process generating 1.2 billion CMs in a stirred tank bioreactor (see figure below, (4)), this cell number represents a volume of about 3-5 ml of sheer cell mass!

Based on our extensive hands-on experience on injecting just about 50-70 million CMs, the application of 750 million CMs to the relatively small macaque heart is an outstanding, difficult-to-control practical challenge. Given these (exemplified) fundamental differences in the experimental procedures, we don't think that the study data by Liu et al, represent a clear contradiction of our results, but rather represent a hardly comparable outcome of different experimental approaches.

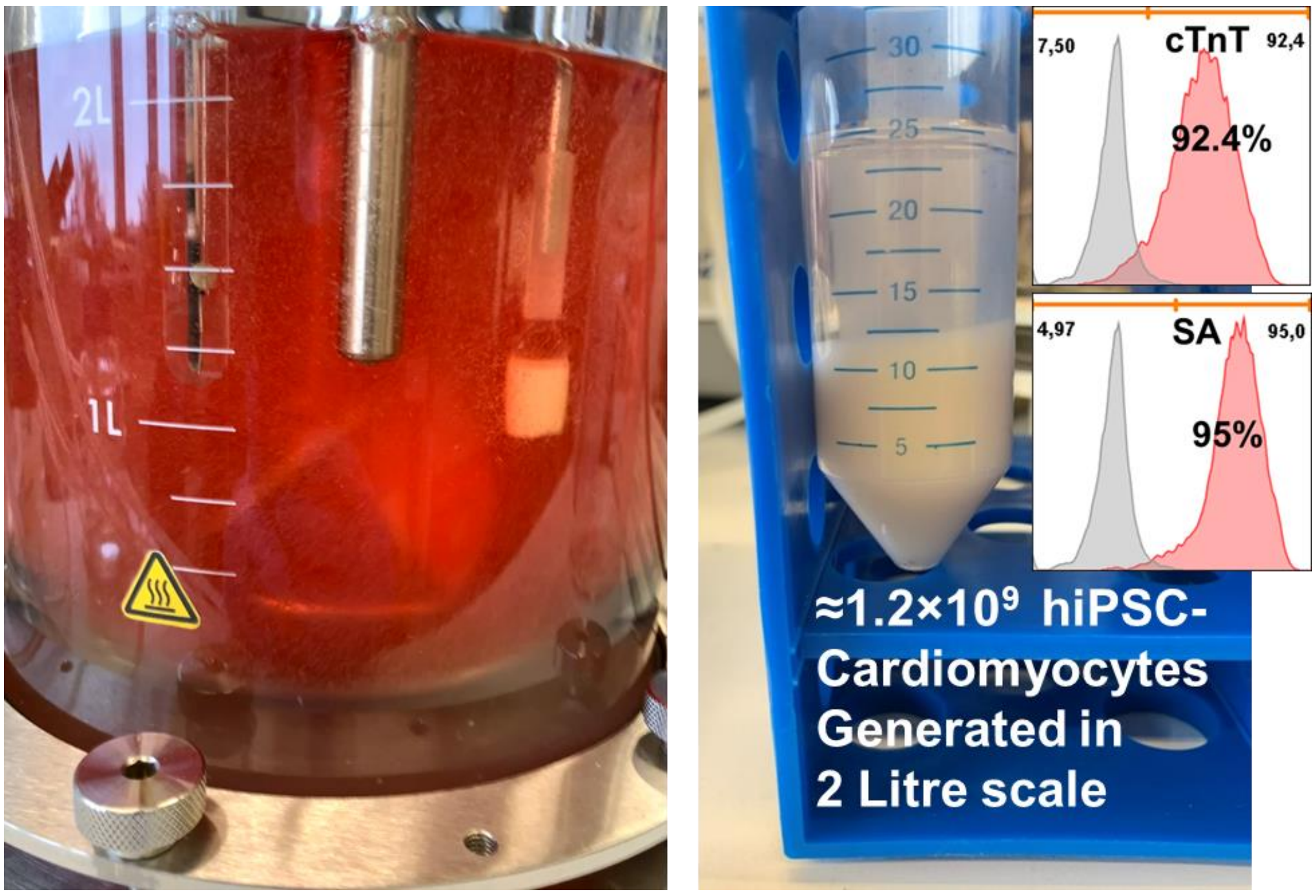

We are also thankful to the reviewer for pointing to the supplemental data of the study by Kobyashi et al. (2), i.e. demonstrating in their mouse study, that aggregate transplantation resulted in approximately threefold higher engraftment than single-cell transplantation. Although these findings generally support our working hypothesis that aggregates promote better cell engraftment (retention, survival etc.), which is also supported by our recent collaborative work in a porcine model by Demkes et al. (5), we would like to respectfully refrain from (further expanding) speculative interpretation of engraftment rates between aggregates and single-cell transplantation, given the inherent heterogeneity between the different animal models (mice, pigs, primates).

Ultimately, our data as well as the related studies in the field do suggest safety of the hPSC-CM transplantation approach and, despite all limitations, suggests a trend towards functional improvement of damaged hearts, thus suggesting that careful transition to the clinical translation in patients deems feasible, as curing patients is the ultimate goal of innovative cell therapy development.

References:

1. Liu, Y. W., Chen, B., Yang, X., Fugate, J. A., Kalucki, F. A., Futakuchi-Tsuchida, A., Couture, L., Vogel, K. W., Astley, C. A., Baldessari, A., Ogle, J., Don, C. W., Steinberg, Z. L., Seslar, S. P., Tuck, S. A., Tsuchida, H., Naumova, A. V., Dupras, S. K., Lyu, M. S., ..., and Murry, C. E. 2018. Human embryonic stem cell-derived cardiomyocytes restore function in infarcted hearts of non-human primates. *Nat Biotechnol* 36, no. 7:597. DOI: 10.1038/nbt.4162.
2. Kobayashi, H., Tohyama, S., Ichimura, H., Ohashi, N., Chino, S., Soma, Y., Tani, H., Tanaka, Y., Yang, X., Shiba, N., Kadota, S., Haga, K., Moriwaki, T., Morita-Umei, Y., Umei, T. C., Sekine, O., Kishino, Y., Kanazawa, H., Kawagishi, H., ..., and Fukuda, K. 2024. Regeneration of Nonhuman Primate Hearts With Human Induced Pluripotent Stem Cell-Derived Cardiac Spheroids. *Circulation* 150, no. 8:611. DOI: 10.1161/CIRCULATIONAHA.123.064876.
3. Menasche, P. 2018. Cell therapy trials for heart regeneration - lessons learned and future directions. *Nat Rev Cardiol* 15, no. 11:659. DOI: 10.1038/s41569-018-0013-0.
4. Kriedemann, N., Triebert, W., Teske, J., Mertens, M., Franke, A., Ullmann, K., Manstein, F., Drakhlis, L., Haase, A., Halloin, C., Martin, U., and Zweigerdt, R. 2024. Standardized production

of hPSC-derived cardiomyocyte aggregates in stirred spinner flasks. *Nat Protoc*. DOI: 10.1038/s41596-024-00976-2.

5. Demkes, E., Cervera-Barea, A., Ebner-Peking, P., Wolf, M., Hochmann, S., Scheren, A. S., Bijsterveld, M., van Oostveen, C. M., Jansen, M., Visser, J., Triebert, W., Halloin, C., Dobbe, J. G. G., de Vos, J., Schurz, M., Danmayr, J., Aalders, M. C. G., Boink, G. J. J., Neef, K., Strunk, D., Zweigerdt, R., de Jager, S. C. A., and Sluijter, J. P. G. 2025. Human Cardiac Microtissues Display Improved Engraftment and Survival in a Porcine Model of Myocardial Infarction. *J Cardiovasc Transl Res* 18, no. 3:512. DOI: 10.1007/s12265-025-10596-0.
